# Supplementary material for: Reduced adult stem cell fate specification led to eye reduction in cave planarians
Source: Nat Commun. 2025 Jan 2;16:304. doi: 10.1038/s41467-024-54478-6 (PMC11696554; doi:10.1038/s41467-024-54478-6)
Supplement: Supplementary file 1 — Supplementary Information [file 41467_2024_54478_MOESM1_ESM.pdf]

Supplementary Figures

Figure S1. Triclad phylogeny based on four gene markers (COI, EF, 28S, and 18S rDNA type I and II) places *G. multidiverticulata* as closely related with others surface species of the genus *Girardia*. Maximum Likelihood and Bayesian Inference trees inferred from concatenated dataset. Values at nodes correspond to posterior probability (Bayesian) and bootstrap values (Maximum Likelihood). Scale bar represents the number of substitutions per site.

Figure S2. Eye-body allometry in cave and surface planarians. a) Schematic representation of pharynx anatomy and the landmarks used for length measurements in fixed DAPI-labeled animals. Scale bar, 100 µm. **b)** Simple linear regressions demonstrating cave planarian morphotypes and the surface planarian *G. dorotocephala* present similar pharynx length-body size allometric scaling in fixed animals (*Gm n*=28; *Gm*(d) *n*=19; *Gd n*=29). **c)** No significant difference in pharynx lengths between the fixed cave and surface planarians in small (1-3.5 mm in length - *Gm n*=14; *Gm*(d) *n*=11; *Gd n*=20) and large (3.5-7 mm in length - *Gm n*=14; *Gm*(d) *n*=8; *Gd n*=9) animals. Kruskal-Wallis followed by Tukey's multiple comparisons test**. d)** A significant difference was observed in the numbers of photoreceptor cells in cave planarians versus surface planarians in small (1-5.9 mm in length - *Gm n*=50; *Gm*(d) *n*=52; *Gd n*=32; *Dj n*=22; *Sm n*=20) and large (6-14 mm in length - *Gm n* = 36; *Gm*(d) n=34; *Gd* n = 24; *Dj* n=20; *Sm* n=16) planarians. *G. multidiverticulata* morphotypes present no differences in eye-body allometric scaling between each other. **e)** Similar overall brain lengths between cave and surface planarians in small (1-5.9 mm in length - *Gm n*=25; *Gm*(d) *n*=27; *Gd n*=16; *Dj n*=11; *Sm n*=10) and large (6-14 mm in length - *Gm n*=18; *Gm*(d) *n*=16; *Gd n*=13; *Dj n*=7; *Sm n*=6) animals. **f)** Overall similar pharynx lengths obtained through live images between the cave and surface planarians in small (0-5.9 mm in length - *Gm n*=25; *Gm*(d) *n*=26; *Gd n*=16; *Dj n*=13; *Sm n*=10) and large (6-14 mm in length - *Gm n*=18; *Gm*(d) *n*=17; *Gd n*=13; *Dj n*=5; *Sm n*=6) animals. Abbreviation, *Gm*, *G. multidiverticulata* non-discernible eye; *Gm*(d) *G. multidiverticulata* discernible eyes, *Gd*, *G. dorotocephala*; *Dj*, *Dugesia japonica*; *Sm*, *Schmidtea mediterranea*. Mean ± SD. For **d**, **e**, **f**, One-way ANOVA followed by Tukey's multiple comparisons test. Mean ± SD. R, coefficient of determination. $^{**}p < 0.01$, $^{****}p < 0.0001$; ns, not shown or not significant.

Figure S3. Schematic view of the behavior essay. a) Light-response behaviors were assessed in two different arenas: (i) the test arena contained 12 different light intensity bins that formed a gradient, from dark to light, and (ii) the control arena contained only one monochromatic shade of gray, i.e., a single intensity of light. Animals were placed at the start area in the middle of the arenas. **b)** Using a video camera fixed to an enclosed chamber to avoid the influence of external light variations, animals were recorded for five minutes. Created in BioRender. Saad, L. (2024) BioRender.com/f28z409. **c)** Image of the experiment with optical filters placed on top of the iPad.

Figure S4. Differences in gene expression between cave planarian morphotypes and between cave and surface planarians. a) *tyrosinase* expression in both cave morphotypes. Scale bars, 50 µm. Representative images from n=5 animals. **b)** Presence of normal eyes in *rx3* (*n*=10) and *pax6A* (*n*=12) RNAi animals in *G. multidiverticulata* (non-discernible eyes). Scale bar, 50 µm. **c)** *pax6A* expression in nervous system cells but not in the eyes *G. multidiverticulata* (non-discernible eyes). Representative images from n=5 animals. **d)** Schematic representation of single-eye RNA sequencing. Eyes from both morphotypes of *Girardia multidiverticulata*, as well as *Girardia dorotocephala*, *Schmidtea mediterranea*, and *Dugesia japonica* were surgically isolated. RNA from each individual eye was extracted and libraries for each sample were prepared, resulting in a total of 12 libraries for *G. multidiverticulata* (discernible), 12 for *G. multidiverticulata* (non-discernible), 11 for *G. dorotocephala*, 11 for *S. mediterranea*, and 13 for *D. japonica*. The transcriptome for each species eye was assembled and orthologs for 162 genes reported to be expressed in the *S. mediterranea* eye were predicted by multiple sequence alignment between the four species. Pair-wise comparisons between each cave and surface species were performed,

p-values were calculated by the default DESeq2 Wald test. Final data analyses include violin plots displaying the average expression levels of eye-related genes based on log_2 normalized counts and a heatmap showing differential gene expression comparing the single-eye data between each cave and surface species, as well as between the two cave morphotypes, using pair-wise DESeq2 Wald tests. Created in BioRender. Saad, L. (2024) BioRender.com/f28z409. **e)** Principal components of gene expression analysis between cave and surface species (*Gm n* = 12; *Gd n* = 11; *Dj n* = 13; *Sm n* = 11).

Figure S5. Violin plots of log_2-normalized counts of all eye-related genes of surface and cave planarians grouped by predicted functions, with genes described in Lapan & Reddien (2012). Each dot represents the expression in a single-eye. In all graphs the species order is: *Girardia multidiverticulata* non-discernible eyes (dark blue), *Girardia multidiverticulata* discernible eyes (light blue), *Girardia dorotocephala* (orange), *Dugesia japonica* (beige), and *Schmidtea mediterranea* (purple). (*Gm n* = 12; *Gd n* = 11; *Dj n* = 13; *Sm n* = 11). * Marks the genes showing padj < 0.05 in the DESeq2 Wald test pair-wise between surface and cave planarian. Complete statistical results presented in Supplementary Table 3.

Figure S6. Reduced eye cell production in cave planarians a) Max z-projection of EdU incorporation in 4, (*Gm n*=5, *Gd n*=5), 8 (*Gm n*=4, *Gd n*=4), and 12 (*Gm n*=6, *Gd n*=5) days after soaking in surface and cave planarians. Whole animal (left panels) representing 4 days post-labeling. Images are from the main Figure 6a; they are shown here to label the locations for zoom-ins at right. Scale bar, 200 µm. Right panels displaying the 100 x 100 µm region used for counting the cell numbers in the whole z-projection in red. Scale bar, 50 µm. **b)** During homeostasis *G. dorotocephala* and *G. multidiverticulata* displayed similar overall numbers of EdU incorporation at 4 (*Gm n*=5, *Gd n*=5), 8 (*Gm n*=4, *Gd n*=4), and 12 (*Gm n*=6, *Gd n*=5) days after EdU delivery in the different counting regions. **c)** The two cave morphotypes presented no differences in eye cell incorporation (*Gm n*=18, *n*=9, *n*=16, *Gm*(d) *n*=13, *n*=12, *n*=13, for 4, 8 and 12 days respectively). Intervals were compared with a Mann-Whitney two-tailed t test **d)** Max z-projection of the head showing *pkd1l-2* expression in *G. dorotocephala* (*n*=12) and *G. multidiverticulata* (*n*=12*)*. EdU^+; $pkd1l\text{-}2^+$ double-positive cells were counted from the maximal extension of the brain until the end of the animal head. **e)** Max z-projection of whole animal *piwi-1* FISH images in *G. dorotocephala* and *G. multidiverticulata*. This image is from main Figure 6a, it is shown here to label the locations for zoom ins. Scale bar, 500 µm. The region where these cells were quantified is highlighted and showed in detail in upper right panels (Scale bar, 50 µm). Lower right panels show the three-dimensional reconstruction confocal whole-mount FISH z-stacks using Imaris software. Shown is the segmentation of $DAPI^+$ cells (gray) and $piwi\text{-}1^+$ cells (magenta) (Scale bar, 5 µm). **f)** Percentage of $piwi\text{-}1^+$ cells are similar between *G. dorotocephala* (*n*=5) and *G. multidiverticulata* (*n*=6). **g)** *agat-3* expression shown in a visual plane, demonstrating co-localization with DAPI, used as reference for counting. Representative images from *n*=10 animals for each species. Scale bar, 50 µm. *Gm*, *G. multidiverticulata* non-discernible eye; *Gm*(d) *G. multidiverticulata* discernible eyes, *Gd*, *G. dorotocephala*. Intervals were compared with a Student's two-tailed t test, ns, not significant. Mean ± SD

Figure S7. Irradiation experiments. a) Left panels: *FoxA* expression in *G. dorotocephala* and *G. multidiverticulata* showing progenitors located anteriorly to the pharynx, but not in other regions close to the head. Right panels: *FoxA* expression depletion after irradiation in the prepharyngeal, counted region. Panels with DAPI labeling represent only one visual plane. Panels with only *FoxA* labeling are max z-projections, red boxes represent the region used for counts. Green box shows the detail of pharyngeal $FoxA^+$ cells. The pharynx is marked in blue. Scale bar, 50 µm. **b)** Neoblast depletion after irradiation in *G. dorotocephala* (two left panels) and *G. multidiverticulata* (two right panels); $piwi\text{-}1^{+}$ cells are scattered throughout the body of planarians (left panels for each species) and are absent in irradiated animals (right panels for each species). Scale bar, 50 µm. **c)** Live images of animals 0, 8, and 12 days after irradiation, demonstrating that eyes are still present. Scale bar, 1 mm. *Gm*, *G. multidiverticulata* non-discernible eye;

Gm(d) *G. multidiverticulata* discernible eyes, *Gd*, *G. dorotocephala*. Intervals were compared with a Student's two-tailed t test. test, ****p < 0.0001; **p < 0.01 ns, not significant.

Supplementary Tables

Table S1. List of Dugesiidae and outgroup species used for phylogenetic analyses (see Fig. S1) with respective accession numbers.

Table S2. *Girardia multidiverticulata* sequences generated in this study and used in the phylogenetic analyses.

Table S3. Results of the differential gene expression analysis in planarian eye-related gene orthologs, showing the enriched genes for each comparison. Genes were annotated by alignment with eye-related genes previously reported (Lapan & Reddien 2012). Genes used was considering padj < 0.05, and either up-regulated (log2FoldChange > 1) or down-regulated (log2FoldChange < -1). DESeq2 Wald test pair-wise comparison between: **(a)** *G. multidiverticulata* non-discernible eyes and *G. multidiverticulata* discernible eyes; **(b)** *G. dorotocephala* and *G. multidiverticulata* morphotypes; **(c)** *D. japonica* and *G. multidiverticulata* morphotypes; **(d)** *S. mediterranea* and *G. multidiverticulata* morphotypes.

Table S4. Genes and sequences that were used in fluorescence *in situ* hybridization, together with primers used for cloning.

Table S5. Sequence of each probe, including initiator and spacer sequences used in in situ Hybridization Chain Reaction.

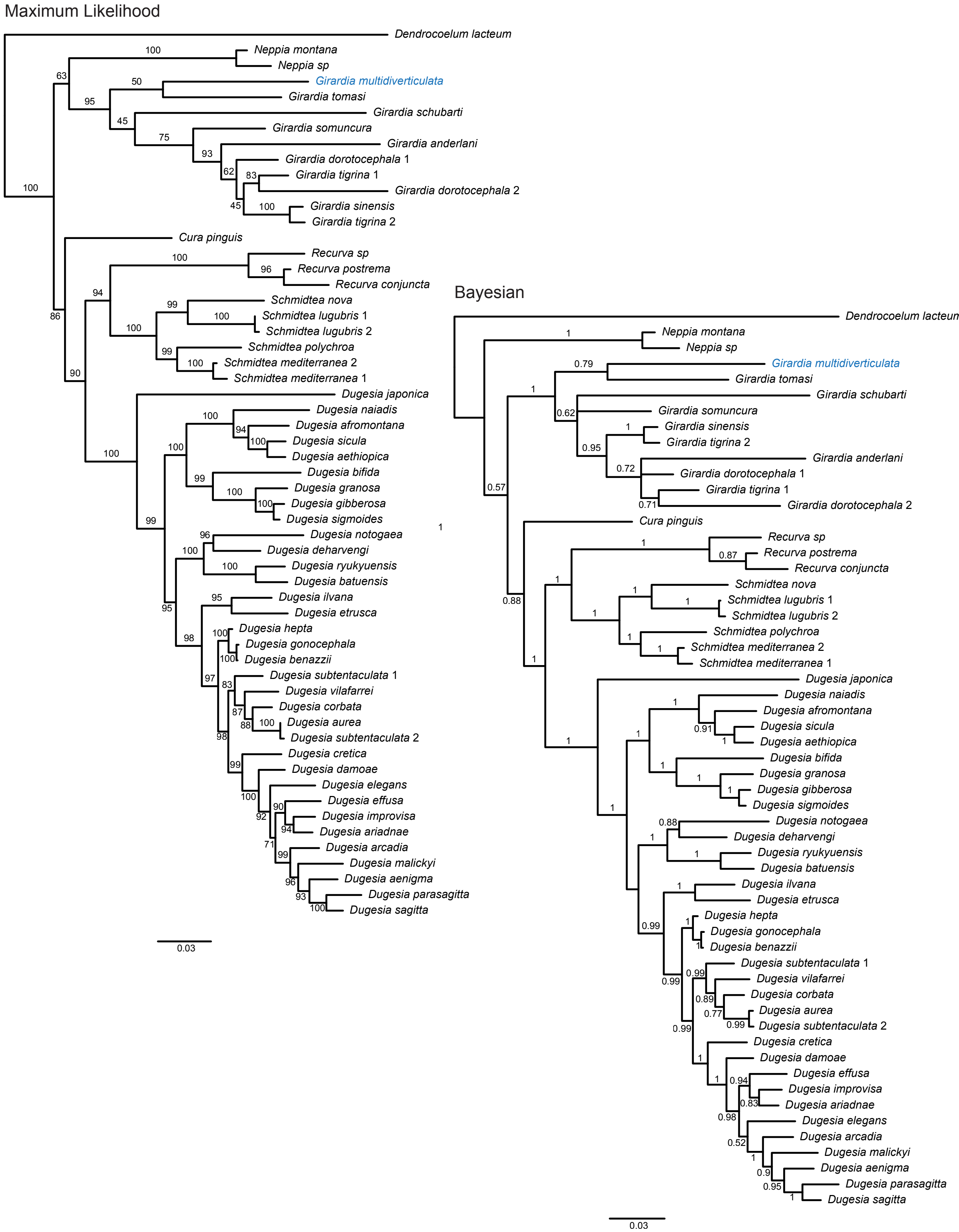
Maximum Likelihood
Dendrocoelum lacteum
Neppia montana
Neppia sp
Girardia multidiverticulata
Girardia tomasi
Girardia schubarti
Girardia somuncura
Girardia anderlani
Girardia dorotocephala 1
Girardia tigrina 1
Girardia dorotocephala 2
Girardia sinensis
Girardia tigrina 2
Cura pinguis
Recurva sp
Recurva postrema
Recurva conjuncta
Schmidtea nova
Schmidtea lugubris 1
Schmidtea lugubris 2
Schmidtea polychroa
Schmidtea mediterranea 2
Schmidtea mediterranea 1
Dugesia japonica
Dugesia naiadis
Dugesia afromontana
Dugesia sicula
Dugesia aethiopica
Dugesia bifida
Dugesia granosa
Dugesia gibberosa
Dugesia sigmoides
Dugesia notogaea
Dugesia deharvengi
Dugesia ryukyuensis
Dugesia batuensis
Dugesia ilvana
Dugesia etrusca
Dugesia hepta
Dugesia gonocephala
Dugesia benazzii
Dugesia subtentaculata 1
Dugesia vilafarrei
Dugesia corbata
Dugesia aurea
Dugesia subtentaculata 2
Dugesia cretica
Dugesia damoae
Dugesia elegans
Dugesia effusa
Dugesia improvisa
Dugesia ariadnae
Dugesia arcadia
Dugesia malickyi
Dugesia aenigma
Dugesia parasagitta
Dugesia sagitta
0.03
Bayesian
Dendrocoelum lacteun
Neppia montana
Neppia sp
Girardia multidiverticulata
Girardia tomasi
Girardia schubarti
Girardia somuncura
Girardia sinensis
Girardia tigrina 2
Girardia anderlani
Girardia dorotocephala 1
Girardia tigrina 1
Girardia dorotocephala 2
Cura pinguis
Recurva sp
Recurva postrema
Recurva conjuncta
Schmidtea nova
Schmidtea lugubris 1
Schmidtea lugubris 2
Schmidtea polychroa
Schmidtea mediterranea 2
Schmidtea mediterranea 1
Dugesia japonica
Dugesia naiadis
Dugesia afromontana
Dugesia sicula
Dugesia aethiopica
Dugesia bifida
Dugesia granosa
Dugesia gibberosa
Dugesia sigmoides
Dugesia notogaea
Dugesia deharvengi
Dugesia ryukyuensis
Dugesia batuensis
Dugesia ilvana
Dugesia etrusca
Dugesia hepta
Dugesia gonocephala
Dugesia benazzii
Dugesia subtentaculata 1
Dugesia vilafarrei
Dugesia corbata
Dugesia aurea
Dugesia subtentaculata 2
Dugesia cretica
Dugesia damoae
Dugesia effusa
Dugesia improvisa
Dugesia ariadnae
Dugesia elegans
Dugesia arcadia
Dugesia malickyi
Dugesia aenigma
Dugesia parasagitta
Dugesia sagitta
0.03

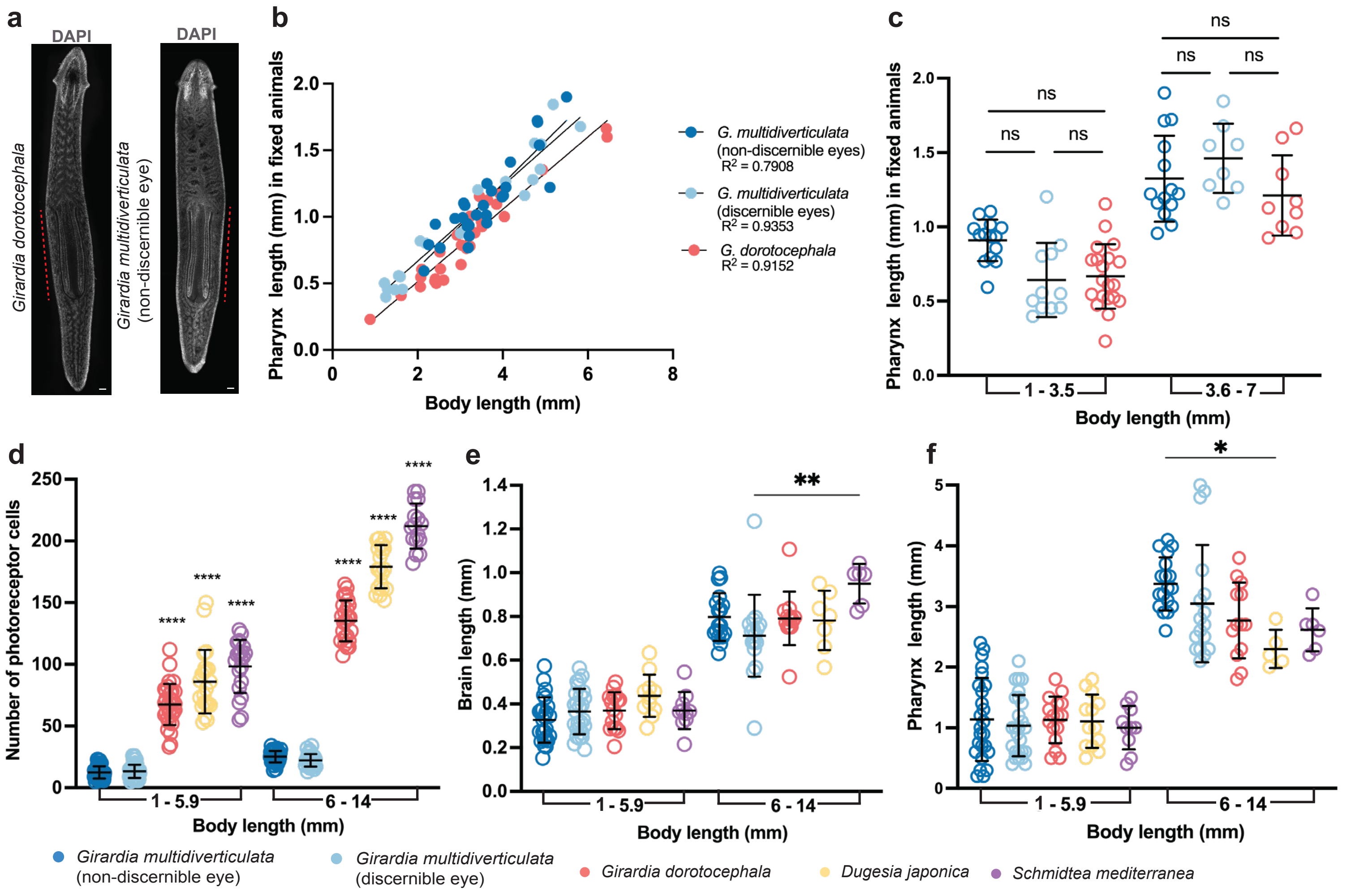
a
DAPI
DAPI
Girardia dorotocephala
Girardia multidiverticulata
(non-discernible eye)
b
Pharynx length (mm) in fixed animals
Body length (mm)
G. multidiverticulata
(non-discernible eyes)
R2 = 0.7908
G. multidiverticulata
(discernible eyes)
R2 = 0.9353
G. dorotocephala
R2 = 0.9152
c
ns
ns
ns
ns
ns
ns
Pharynx length (mm) in fixed animals
1 - 3.5
3.6 - 7
Body length (mm)
d
Number of photoreceptor cells
****
****
****
****
****
****
1 - 5.9
6 - 14
Body length (mm)
e
Brain length (mm)
**
1 - 5.9
6 - 14
Body length (mm)
f
Pharynx length (mm)
*
1 - 5.9
6 - 14
Body length (mm)
Girardia multidiverticulata
(non-discernible eye)
Girardia multidiverticulata
(discernible eye)
Girardia dorotocephala
Dugesia japonica
Schmidtea mediterranea

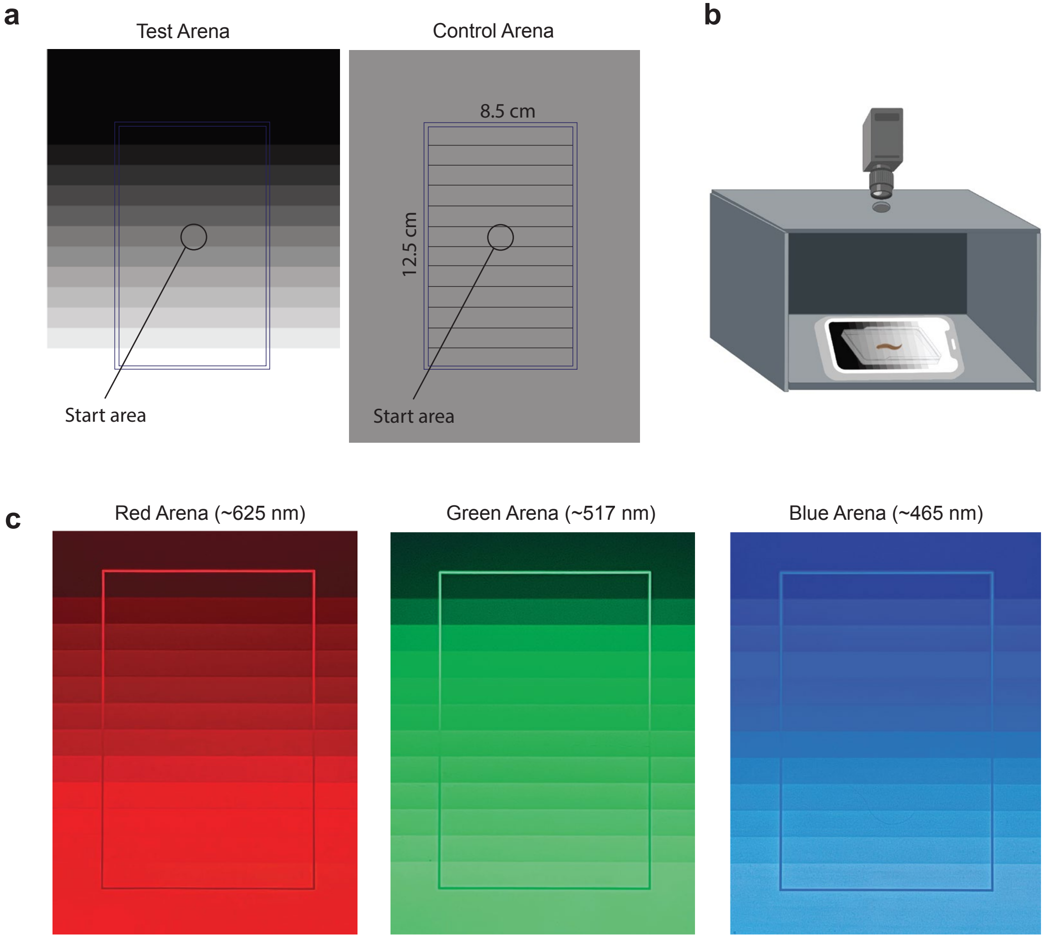
a
Test Arena
Control Arena
8.5 cm
12.5 cm
Start area
Start area
b
c
Red Arena (~625 nm)
Green Arena (~517 nm)
Blue Arena (~465 nm)

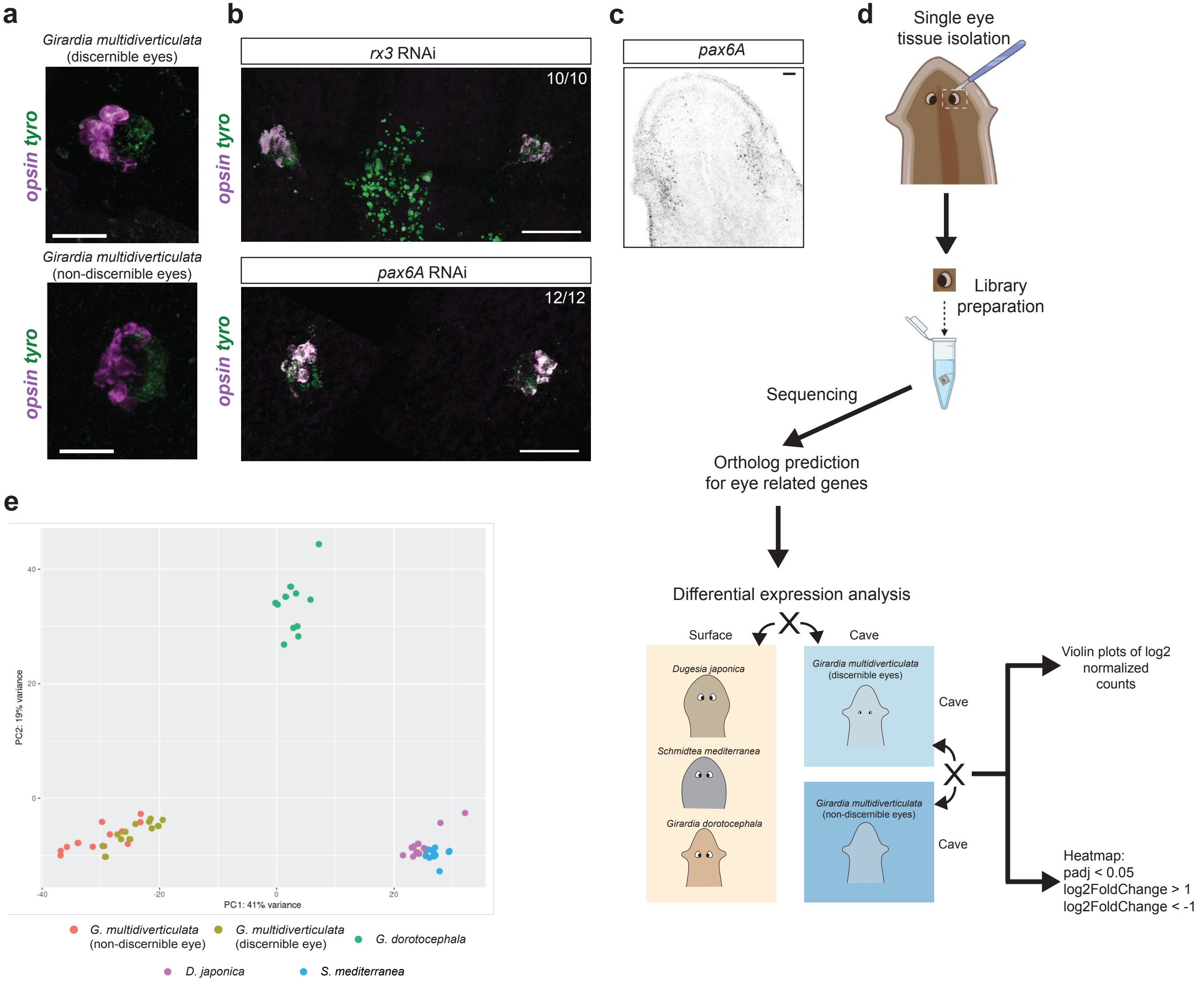
a
Girardia multidiverticulata (discernible eyes)
opsin tyro
Girardia multidiverticulata (non-discernible eyes)
opsin tyro
b
rx3 RNAi
10/10
opsin tyro
pax6A RNAi
12/12
opsin tyro
c
pax6A
d
Single eye tissue isolation
Library preparation
Sequencing
Ortholog prediction for eye related genes
Differential expression analysis
Surface
Cave
Dugesia japonica
Schmidtea mediterranea
Girardia dorotocephala
Girardia multidiverticulata (discernible eyes)
Cave
Girardia multidiverticulata (non-discernible eyes)
Cave
Violin plots of log2 normalized counts
Heatmap:
padj < 0.05
log2FoldChange > 1
log2FoldChange < -1
e
PC2: 19% variance
PC1: 41% variance
40
20
0
-40
-20
0
20
G. multidiverticulata (non-discernible eye)
G. multidiverticulata (discernible eye)
G. dorotocephala
D. japonica
S. mediterranea

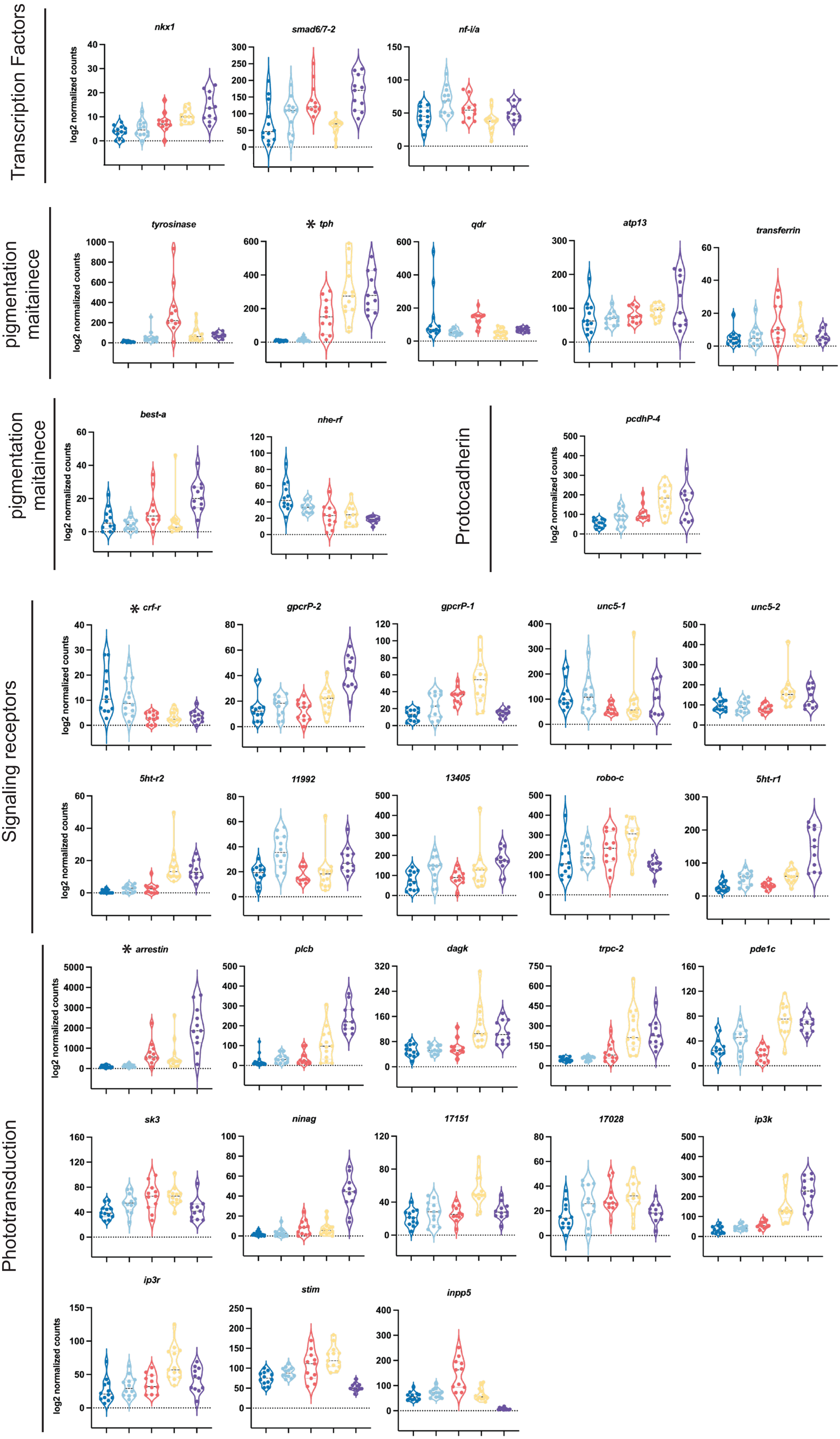
Transcription Factors
nkx1
smad6/7-2
nf-i/a
melanin synthesis
pigmentation
maitainece
tyrosinase
* tph
qdr
atp13
transferrin
melanin synthesis
pigmentation
maitainece
best-a
nhe-rf
Protocadherin
pcdhP-4
Signaling receptors
* crf-r
gpcrP-2
gpcrP-1
unc5-1
unc5-2
5ht-r2
11992
13405
robo-c
5ht-r1
Phototransduction
* arrestin
plcb
dagk
trpc-2
pde1c
sk3
ninag
17151
17028
ip3k
ip3r
stim
inpp5
log2 normalized counts

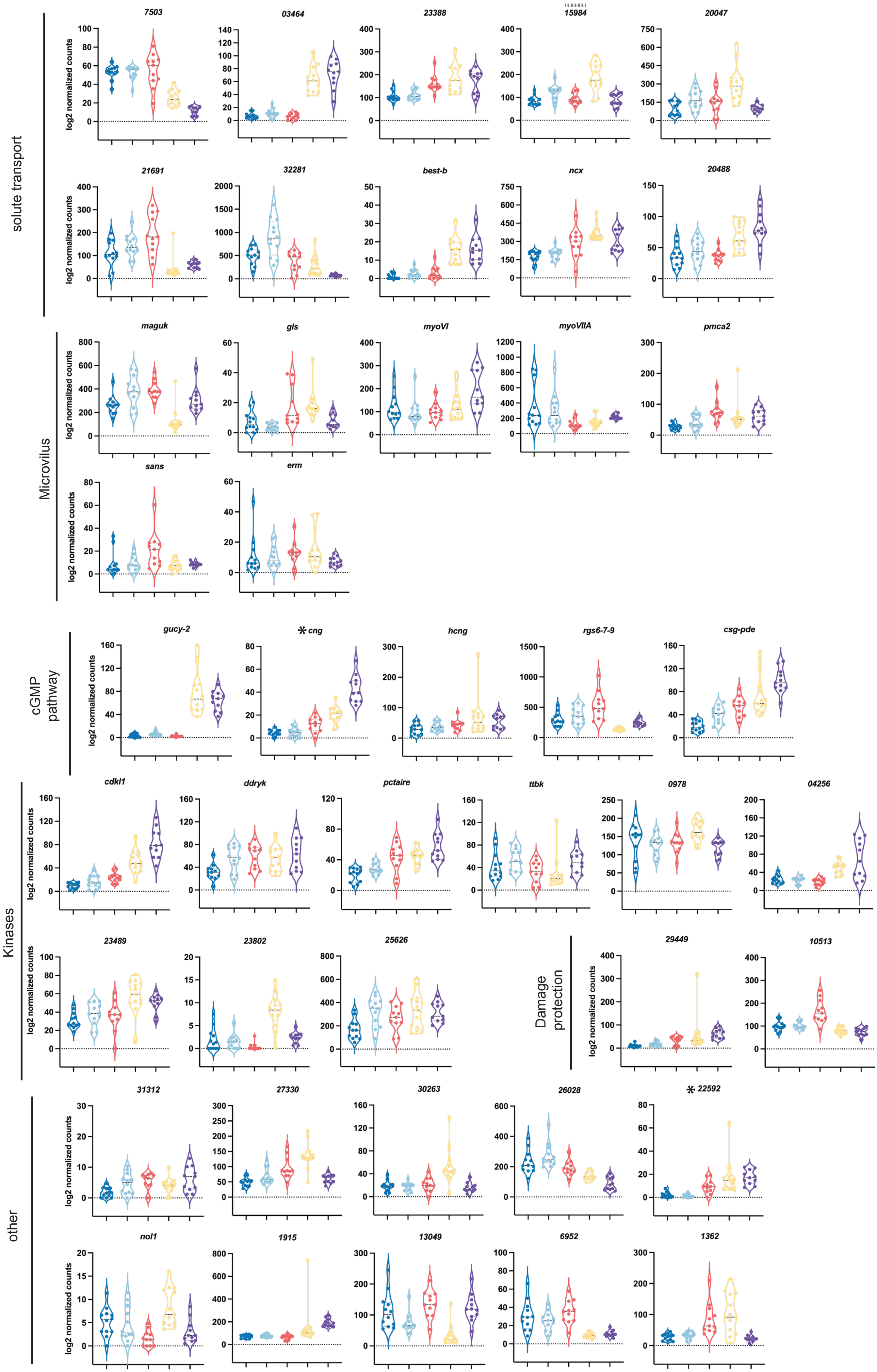
solute transport
7503
03464
23388
15984
20047
21691
32281
best-b
ncx
20488
Microvilus
maguk
gls
myoVI
myoVIIA
pmca2
sans
erm
cGMP pathway
gucy-2
*cng
hcng
rgs6-7-9
csg-pde
Kinases
cdkl1
ddryk
pctaire
ttbk
0978
04256
23489
23802
25626
Damage protection
29449
10513
other
31312
27330
30263
26028
*22592
nol1
1915
13049
6952
1362
log2 normalized counts

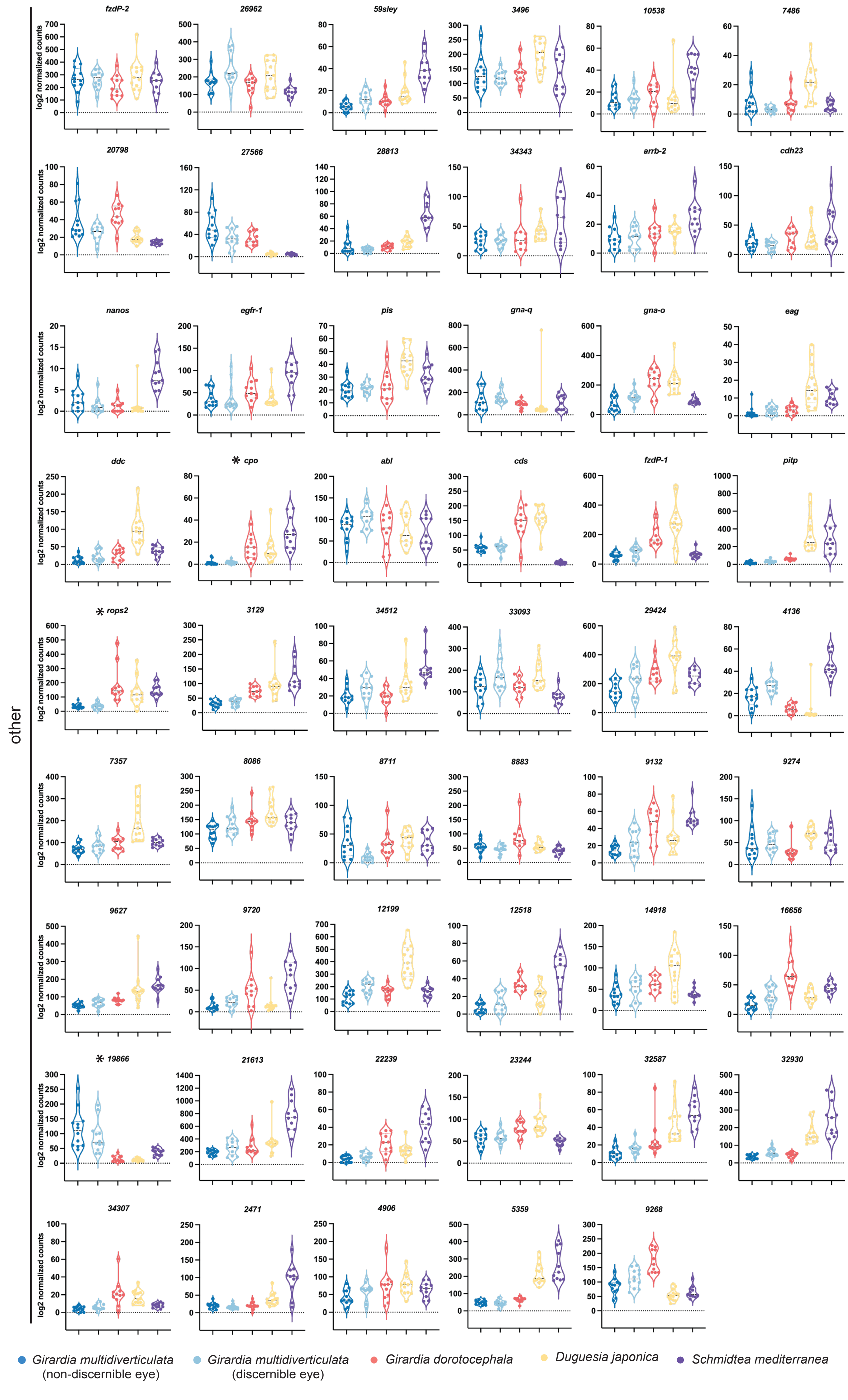

other
log2 normalized counts
fzdP-2
26962
59sley
3496
10538
7486
20798
27566
28813
34343
arrb-2
cdh23
nanos
egfr-1
pis
gna-q
gna-o
eag
ddc
* cpo
abl
cds
fzdP-1
pitp
* rops2
3129
34512
33093
29424
4136
7357
8086
8711
8883
9132
9274
9627
9720
12199
12518
14918
16656
* 19866
21613
22239
23244
32587
32930
34307
2471
4906
5359
9268
Girardia multidiverticulata (non-discernible eye)
Girardia multidiverticulata (discernible eye)
Girardia dorotocephala
Duguesia japonica
Schmidtea mediterranea

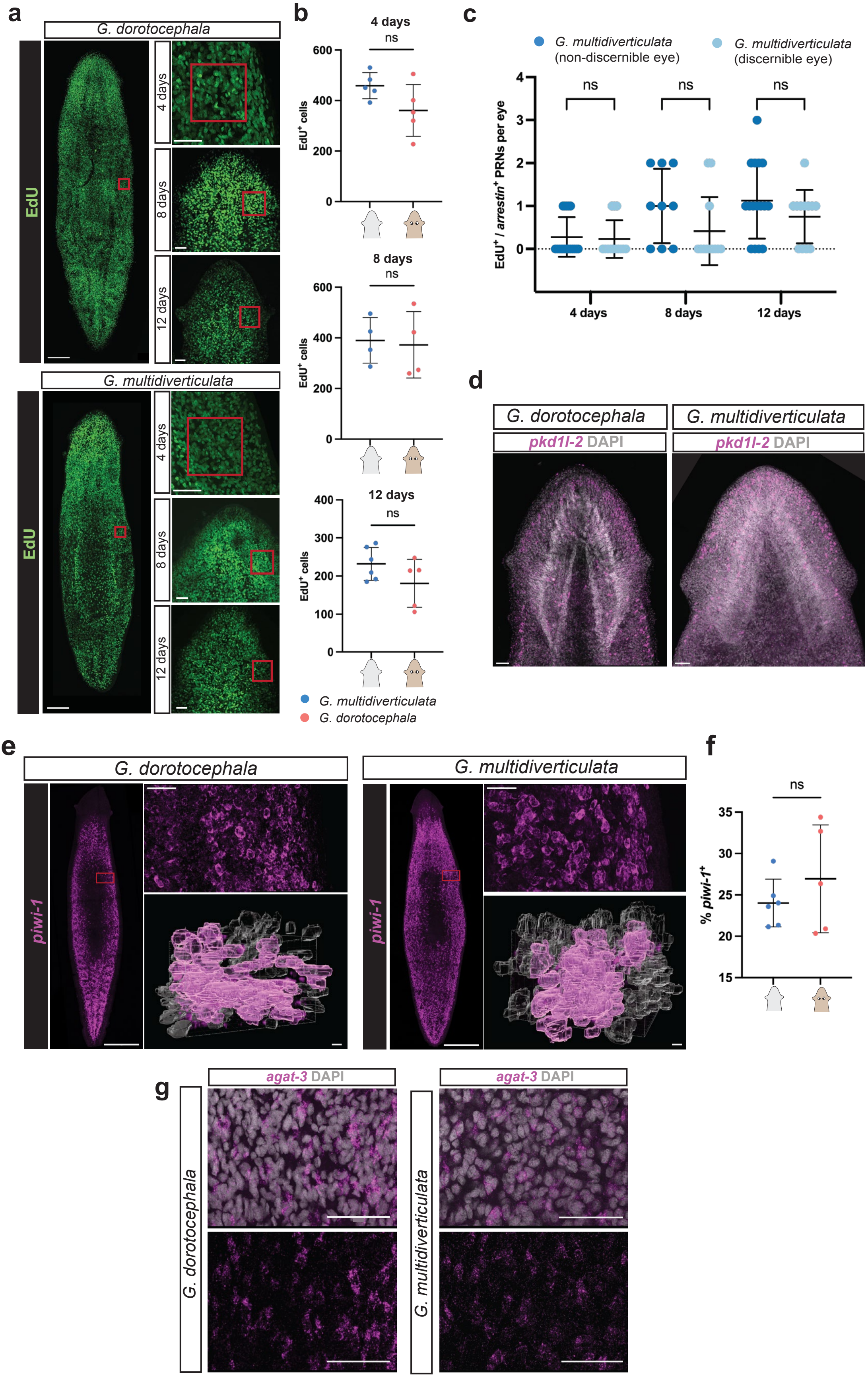

a
G. dorotocephala
EdU
4 days
8 days
12 days
G. multidiverticulata
b
4 days
ns
EdU+ cells
600
400
200
0
8 days
12 days
300
100
G. multidiverticulata
G. dorotocephala
c
G. multidiverticulata (non-discernible eye)
G. multidiverticulata (discernible eye)
EdU+ / arrestin+ PRNs per eye
4
3
2
1
0
4 days
8 days
12 days
d
pkd1l-2 DAPI
e
piwi-1
f
% piwi-1+
35
30
25
20
15
g
agat-3 DAPI

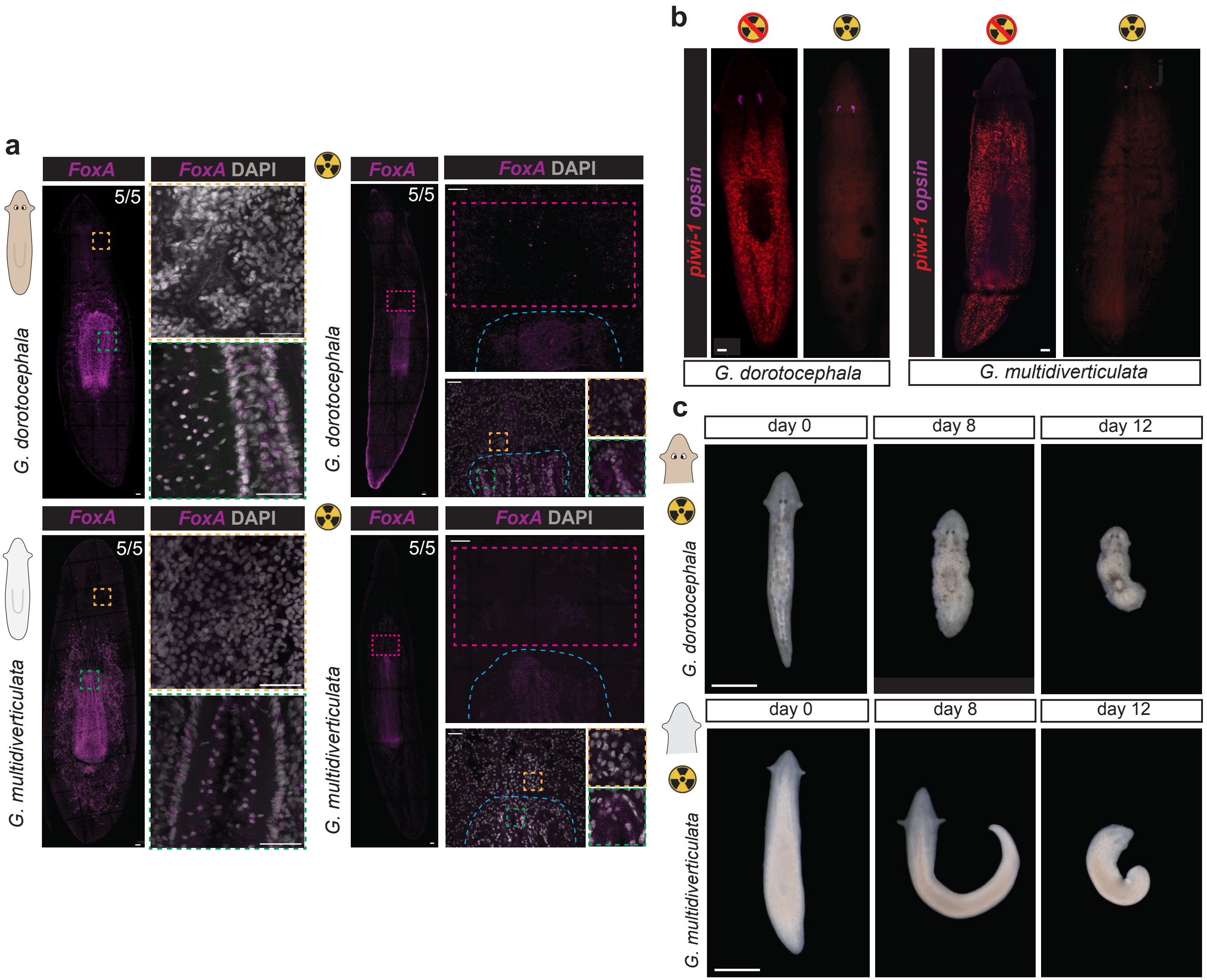

a
FoxA
FoxA DAPI
5/5
G. dorotocephala
G. multidiverticulata
b
piwi-1 opsin
G. dorotocephala
G. multidiverticulata
c
day 0
day 8
day 12
G. dorotocephala
G. multidiverticulata

Table S1. List of Dugesiidae and outgroup species used for phylogenetic analyses with respective accession numbers.

Species	GenBank accession number				
	18S		28S	CO1	EF
	Type I	Type II			
Family Dugesiidae Ball, 1974					
Genus *Girardia* Ball, 1974					
Girardia anderlani		DQ666013	DQ665972	DQ666038	
Girardia dorotocephala 2				AF178314	
Girardia dorotocephala 1				KM200929	
Girardia schubarti		DQ666015	DQ665976	DQ666041	KJ599691
Girardia tigrina 1	AF013157	AF013156	DQ665977	AF178316	AJ250913
Girardia tigrina 2				KM200930	
Girardia sinensis				KP091892	
Girardia somuncura				MW271869	
Girardia tomasi				MW271863	
Genus *Cura* Strand, 1942					
Cura pinguis	AF033043		DQ665963	AF178309	KJ599684
Genus *Schmidtea* Ball, 1974					
Schmidtea mediterranea 1	DJARR18SB	DMU31085	MG457267	AF178322	KJ599709
Schmidtea mediterranea 2	DMU31084			JF837055	
Schmidtea polychroa	AF013152	AF013154	DQ665993	AF287057	AJ250914
Schmitdea lugubris 1			MG457269	MG457275	
Schmidtea lugubris 2				AF290019	
Schmidtea nova			MG457273	MG457277	
Genus *Duguesia* Girard, 1850					
Duguesia aenigma	KF308698			KC006964	
Duguesia aethiopica	KY498822		KY498806	KY498845	
Duguesia afromontana	KY498823		KY498807	KY498846	
Duguesia arcadia	KF308694			KF308723	
Duguesia ariadnae				JN376142	
Duguesia aurea			MK712523	MK712632	
Duguesia batuensis			KF907823	KF907819	
Duguesia benazzii			MK712509	FJ646933	
Duguesia bifida	KY498829		KY498813	KY498851	
Duguesia corbata			MK712525	MK712637	
Duguesia cretica	KF308697			JN376141	
Duguesia damoae				KC006978	

Duguesia deharvengi			KF907824	KF907820	
Duguesia effusa				KC006980	
Duguesia elegans	KF308695			KC006984	
Duguesia etrusca				AF178310	
Duguesia gibberosa	KY498842		KY498819	KY498857	
Duguesia gonocephala		DQ666002	DQ665965	DQ666033	
Duguesia granosa	KY498833		KY498816		
Duguesia hepta			MK712512	FJ646943	
Duguesia ilvana				FJ646944	
Duguesia improvisa	KF308696			KC006986	
Duguesia japonica	AF013153	DJA18SRNAA	DQ665966	DQ666034	D49924
Duguesia malickyi				KF308748	
Duguesia naiadis				KF308755	
Duguesia notogaea	KJ599713		KJ599720	FJ646945	KJ599687
Duguesia parasagitta				KF308738	
Duguesia ryukyuensis		AF050433	DQ665968	AB618488	KJ599688
Duguesia sagitta				KC006996	
Duguesia sicula	KF308693		DQ665969	KC536630	KJ599689
Duguesia sigmoides			KY498811	KY498849	
Duguesia subtentaculata 1		AF013155	MK712531	DQ666036	KJ599690
Duguesia subtentaculata 2				FJ646949	
Duguesia vilafarrei			MK712511	MK712648	
Genus *Neppia* Ball, 1974					
Neppia montana	AF050432			AF178319	
Neppia sp.	DQ665999		DQ665982	DQ666046	KJ599695
Genus *Recurva* Sluys, 2013					
Recurva postrema	KF308691		MG457274	KF308763	
Recurva sp				KF308764	
Recurva conjuncta	KF308692				
Outgroup					
Dendrocoelum lacteum	AJ312271		DQ665967	AF178312	

Table S2. ***Girardia multidiverticulata*** **sequences generated in this study and used in the phylogenetic analyses.**

Gene	Sequence
18S1	GCCACAAGATAACTGTGGTAATTCTAGAGCTAATATTTAACAGAATGCCGTGACTC<br>ACGAAGCGGCGGATTTATTAGATCAAAATCAACCTGCTGAAAGGCGGTTTTTTGA<br>TGACTCTGGATAACTTTCTGATCGTACGACTTAGTGTTGACGACATATCTCTTGAA<br>ATGGCTGACCTATCAACTTTCGATGGTAAGATCAAAGCTTACCATGGTTGTAACG<br>GGTAACGGGGAATCAGTGTTCGATTCCGGAGAGGGAGCCTGAGAAACGGCTACC<br>ACATCCACGGAAGGCAGCAGGCGCGCAAATTACCCAATACCGGTTCGGTGAGGT<br>AGTGACAAAAAATAACAATATGGGCCCTAGTGGTTTCATAATTGCAATGAGAACAT<br>TTTAAATACTTTATCGAGTATCAATTGGAGGGCAAGTCTGGTGCCAGCAGCCGCG<br>GTAATTCCAGCTCCAATAGCGTATATTAAAGTTGTTGCAGTTAAAAAGCTCGTAGT<br>TGAAATTGAGGAAAAAGATGGTAAATCTACGATTGAGCTATCTTGTCCTTCGACGT<br>CGAGTATATCTTTGATGCTCTTTAATGGGTGTTGATGACCGACAAGTTTACTTTGA<br>AAAAATTAGAGTGCTTAAAGCAGGCTTATGCTTGCATATTGTTGCATGGAATAATA<br>AAATAGGACTTCGGTTTTATTTTGTTGGTTTTCGAAACTGAAGTAATGATTAAAAGA<br>GACTGCCGGGGGCATATGTATGCTGGTGCTAGAGGTGAAATTCTTAGATCATCAG<br>CAGACAAACAAC
18S2	AAACCGCGGATGGCTCATTATAACAGCTATGATTTGAGAGATTTAATCTTTTTGCC<br>ACAAGATAACTGTGGTAATTCTAGAGCTAATATTTAACAGAATGCCGTGACTCACG<br>AAGCGGCGGATTTATTAGATCAAAATCAACCTGCTGAAAGGCGGTTTTTTGATGA<br>CTCTGGATAACTTTACTGATCGTACGACAGTGTTGACGACATATCTCTTGAAATGG<br>CTGACCTATCAACTTTCGATGGTAAGATCAAAGCTTACCATGGTTGTAACGGGTAA<br>CGGGGAATCAGTGTTCGATTCCGGAGAGGGAGCCTGAGAAACGGCTACCACATC<br>CACGGAAGGCAGCAGGCGCGCAAATTACCCAATACCGGTTCGGTGAGGTAGTGA<br>CAAAAAATAACAATATGGGCCCTAGTGGTTTCATAATTGCAATGAGAACATTTTAA<br>ATACTTTATCGAGTATCAATTGGAGGGCAAGTCTGGTGCCAGCAGCCGCGGTAAT<br>TCCAGCTCCAATAGCGTATATTAAAGTTGTTGCAGTTAAAAAGCTCGTAGTTGAAA<br>TTGAGGAAAAAGATGGTTTGAAATCTACGATTGATAGCTATTTGTCCTTCGTCGAG<br>TATATCTTTGATCTCTTTAATGGGTGTTGAGGATGACCGACAAGTTTACTTTGAAA<br>AAATTAGAGTGCTTAAAGCAGGCTTATGCTTGCATATTGTTGCATGGAATAATAAA<br>ATAGGACTTCGGTTTTATTTTGTTGGTTTTCGAAACTGAAGTAATGATTAAAAGAG<br>ACTGCCGGGGGCATATGTATGCTGGTGCTAGAGGTGAAATTCTTAGATCATCAGC<br>AGACAAACAACTGCGAAAGCATTTGCCAAGAATGTTTTCATTAATCAAGAACGAAA<br>GTCAGAGGATCGAAGACGATCAGATACCGTCCTAGTTCTGACCATAAACTATGCC<br>AACTGACAGTTCGCGAAGGTAATTCAAATCTCCTTGCGAGAAGTCACCGGGAAAC<br>CAAGTCTATGGGTTCCGGGGGAAGTATGGTTGCAAAGCTGAAACTTAAAGGAATT<br>GACGGAAGGGCACCACCAGGAGTGGAGCCTGCGGCTTAATTTGACTCAACACGG<br>GAAATCTCACCCGGTCCGGACACTGTGAGGATTGACAGATTGATAGCTCTTTCTT<br>GATTCGGTGGTTGGTGGTGCATGGCCGTTCGTAGTTGGTGGAGCGATTTGTCTG<br>GTTTATTCGTTAACGAACGAGACTCACCTGCTAAATAGTACATTTGTGTTAAATCG<br>CAAATGCTGACTTAGAGGAATAAATAGCGTTTAGCTAAATGAAATGGAGCAATAAC<br>AGGTCTGTGATGCCCTTAGATGTCCGGGGCCGCACGCGCGCTACAATGGCAGTA<br>CCAGCGAGTTTTATTCCTAGCTAGAAATGGCCGGGTAACCTGCTGAATCACTGTC<br>GTGTCAGGAATAGTGGATTGAAATTATCCCACTTGAACGAGGAATTCCTAGTAAG<br>CGCAAGTCACTAGCTTGCGCTGATTACGTCCCTGCCCTTTGTACACACCGCCCGT<br>CGCTACTACCGATTGACCCCTTGATGCGGTCATTGTCTTATTTACCGACCCCCTAC<br>TTAA

28S	TACTCCAAATTCACTTGATTGTGATATTGTCCAGAGAGGGTGTTAGACCCGTTGGA<br>GTATGCGAGACTTTTATTGATGCTTCCTATGAGTCGGATTGTTTGAGAATGCAGTC<br>CAAATTGGGTGGTAAACTCCATCTAAGGCTAAATACTTGTGTGAGTCCGATAGCA<br>AACAAGTACCGTGAGGGAAAGTTGAAAAGAACTTTGAAGAGAGAGTTAAATAGTA<br>CGTGAAATCGCTTAGAGGTAAACGAGTGGAGCCGAAGCTGATATATGGAATTCAG<br>TTGTTTGTATTGTTTTCAAATTCGGAAACAGATTTTTTTTGAACTGACCGGATTTGA<br>CTTTTATTTTACAATCAATGCACTTTCCATATATCAAAACTGCGACCAATAGTACGA<br>TTTCTCTGTCGTTGGGGGAAGGTGACCAGTTCTTCGGAATCGGTTTTATAGCCCT<br>TTGCACGGTTAAATAGTGTATTGGTAATTGCGATTTGCAAATTTTGATATTATCAAT<br>GTTGATGATTTGAATTCGAGCTTTTTCGAGATCGGGTTTGAATTGTTGATGTAGTA<br>GATTTTCAAAGTGTAAATCTAAAGTCTGCAGTATATGTAGGTTACCTACTCGTCCC<br>GTCTTGTAACACGGACCAAGGAGTTTTATATGATCGCAAGTCAAAGGGCTATACG<br>AAACCCCATGGCGAAATTAATGTAAAGGTTTGCTTCGGTGAACTGTGATGTGACC<br>TTGTTCGGTAACGAGTGCAACATCAACCCGTCTGAAGGTGTTCGCATCTGGGCG<br>GAGTTTGAGCGATCGTGTAAAGACCCGAAAGATGGTGAACTATGCTTGTGCAGGT<br>TGAAGTCAGAGGAAACTCTGATGGAAGACCGCCGCGGTTCTGACGTGCAAATCG<br>ATCGTCGGACATGAGTATAGGGGCGAAAGACTAATCGAACCATCTAGTAGCTGGT<br>TCCTTCCGAAGTTTCCCTCAGGATAGCTGGCGTTTATTTCCGTAGTTGAACCTGGT<br>AAAGCGAATGATTAGAAGTATTGGGGGCGAAATGCTCTCAACTTATTCTCAAACTT<br>TAAATGGGTTCGAAGTGCGCCTCTTTTTGTTGGAGGTGTACATCGAATACAAATG<br>CCTAGTGGGCCATTTTTGGTAAGCAGAACTGGCGATGTGGGATGAACCAAACATC<br>CGGTTAAGGTGCCTAACATCACGCTCATGAGAAACCACAAAAGGTGTTGGTTGAT<br>AAAGACAGCAGGACGGTGGACATGGAAGTCGTCATCCGCTAAGGAGTGTGTAAC<br>AACTCACCTGCCGAATCAACCAGCCCTGAAAATGGATGGCGCTGTAGCGTGGGA<br>CCTATACCGGATCGGTATTGTAAGCGAGTAAACAATACCGTGTAG
COI	TGGTTGTTGTTTCCGTCTGGTTTAGGGTCAGGGTGGACTTTATATCCGCCTTTGG<br>ATCTTGCTATTTTTTCGTTACATCTTGCTGGGGTTAGGTCAATTTTGGGAGCTATA<br>AATTTTATAACAACAGTTTTGTTTGTGTGATCTATAATGATTACTGCGTTTTTGTTG<br>GTGTTGGCTTTACCGGTGTTGGCTGCGGGGTTGACAATGTTATTGACTGATCGGA<br>ATTTTAATACGACCTTTTTTGATCCTAGAGGTGGTGGAGATCCCATTTTGTTTCAG<br>CACATATTTTGGTTTTTTGGACATCCTGAGGTTTATATTTTGATTTTACCAGGGTTT<br>GGAATGATATCTCAGATTGTGATTTATTATAGTGGGAAGTGTTTTTCGTTTGGTCA<br>TTTGGGGATGGTTTTTGCTATGGTGAGGATTGGGTTTTTGGGTTTTATTGTGTGGG<br>CTCATCACATGTATACAGTAGGTATGGATTTTGATTCTCGGGCTTACTTTACGGGG<br>GTGACTATGATTATAGGAGTGCCGACCGGTATAAAGGTTTTCAGGTGATTGGCGA<br>CATTGTATGGTGGTAGTTGAGATTTGTTGTCTATTTCTTTGTTGTGGGGTTTGGGG<br>TTTGTATATTTG
EF	GATTTCATCAAAAACATGATTACCGGCACCTCCCAAGCCGATTGTGCTGTCCTCAT<br>TGTTGCTGCTGGTGTCGGTGAATTCGAAGCCGGTATTAGCAAGAACGGTCAAACC<br>CGAGAACACGCCTTGCTCGCTTACACTCTCGGTGTCAAACAGATGATTGTCGGTA<br>TCAACAAAATCGATTCAGCTGAACCACCATACAGTGAAGCTCGATACAACGAAAT<br>CAAGAAGGAAGTTTCCGGTTATATCAAGAAAGTTGGTTACAATCCCGATGCCGTT<br>GCTTTCGTGCCAATCTCCGGATGGTGCGGTGACAACATGGTGTCCCCCAGCACC<br>AACATGCCATGGTTTAAGGGATGGACCATCAAGAGAAAGAATGGAACAACAAAGA<br>AAGACGAAAGCCTTAATGGTACCACTCTCCTCGAAGCAATTGATGCCATCGACCC<br>ACCAGCAAGACCAACCGATAAGCCACTCCGTCTGCCTCTGCAAGACGTCTACAAG<br>ATCGGTGGTATTGGAACAGTGCCTGTTGGTCGAGTTGAAACCGGTATTCTTAAAC<br>CTGGAATGGTTGTCTCTTTCGCTCCTCACGGTTTGACTACTGAAGTTAAATCCGTC<br>GAAATG

Table S3a. Results of the differential gene expression analysis in planarian eye-related gene orthologs. DESeq2 Wald test pair-wise comparison between *G. multidiverticulata* non-discernible eyes and *G. multidiverticulata* discernible eyes.

gene	baseMean	log2FoldChange	lfcSE	stat	pvalue	padj
gir_mul_tyrosinase	27,8884105	-2,43897026402606	0,433610197	-5,62479914	1,86E-08	2,41E-05
gir_mul_cgs-pde	23,68026708	-1,13644421422913	0,253685816	-4,47973101	7,47E-06	0,001613077
gir_mul_11992	21,59106122	-1,12798393275862	0,273204632	-4,12871452	3,65E-05	0,004972759
gir_mul_32930	38,40833848	-0,908727438584708	0,231485568	-3,92563323	8,65E-05	0,006852252
gir_mul_08711	17,45938549	1,80900535075061	0,463286025	3,904726783	9,43E-05	0,007185871
gir_mul_12199	124,8429713	-0,89452233723452	0,231372301	-3,86616001	0,000110562	0,007954352
gir_mul_59sley	7,233482309	-1,38950044839832	0,387521221	-3,58561124	0,00033629	0,015306553
gir_mul_25626	195,6131572	-1,02504204068578	0,28414477	-3,60746405	0,000309204	0,015306553
gir_mul_15984	80,87413023	-0,594431477586589	0,167423531	-3,55046555	0,00038455	0,016327635
gir_mul_16656	18,97676746	-1,14190339580359	0,332459147	-3,43471794	0,000593171	0,020826519
gir_mul_5ht-r1	32,87165575	-0,950680366472501	0,275888426	-3,44588709	0,000569188	0,020826519
gir_mul_32281	548,3123203	-0,919662356118758	0,275576665	-3,33722871	0,000846183	0,024083666
gir_mul_qdr	70,0873597	1,13307158238502	0,348936036	3,247218584	0,001165388	0,028779622
gir_mul_29449	11,02201627	-1,07280772419692	0,340477311	-3,15089343	0,001627719	0,037251596
gir_mul_ddryk	34,57056555	-0,890490781408689	0,28668838	-3,10612792	0,001895547	0,039946388
gir_mul_nf-i-a	44,62761259	-0,658699191849414	0,212080605	-3,10589076	0,001897068	0,039946388
gir_mul_maguk	251,9210221	-0,638779774105776	0,211280886	-3,02336754	0,002499784	0,046246011
gir_mul_soxB	17,08815028	-1,02577256205706	0,341097816	-3,00726804	0,002636072	0,048080471
gir_mul_20047	106,8607268	-0,906938839273828	0,309464869	-2,93066816	0,003382339	0,05675981
gir_mul_pcdhP-4	55,6662357	-0,809775204053934	0,277503087	-2,91807638	0,003521981	0,057002562
gir_mul_gna-o	74,89381194	-0,901279942512317	0,32018123	-2,81490561	0,004879156	0,069322245
gir_mul_26962	164,1040443	-0,60982978466117	0,217291386	-2,80650695	0,005008183	0,069522855
gir_mul_sk3	37,18699555	-0,52629559273531	0,188641593	-2,78992339	0,005272052	0,069580797
gir_mul_04136	17,26569574	-0,757163604800711	0,275596713	-2,7473608	0,006007701	0,075902168
gir_mul_27330	46,60445193	-0,572311651593925	0,208215597	-2,74864929	0,005984138	0,075902168
gir_mul_09132	15,64923556	-0,885738926666073	0,328576137	-2,69568854	0,007024331	0,084008706
gir_mul_gpcrP-1	14,36269872	-0,97476381397459	0,365980567	-2,66343052	0,007734838	0,088113009
gir_mul_21613	179,3358946	-0,59588568819918	0,224085166	-2,65919292	0,00783281	0,088113009
gir_mul_eya	15,82033178	-0,666578900499514	0,25388739	-2,62549039	0,008652425	0,092221322
gir_mul_32587	10,43379336	-0,785168661501633	0,300660802	-2,61147664	0,009015215	0,094150831
gir_mul_meis	25,29732707	-0,598951532828688	0,231488242	-2,58739505	0,009670465	0,097611075
gir_mul_13405	82,12206428	-0,897689863135459	0,349731161	-2,56679977	0,010264186	0,099045498
gir_mul_09268	76,60574353	-0,51014249506562	0,198905843	-2,56474363	0,010325206	0,099045498
gir_mul_pitp	20,53902862	-0,903098403103648	0,354495868	-2,54755693	0,010848015	0,101798404
gir_mul_29424	151,5491617	-0,644769880298167	0,253509009	-2,54338054	0,010978559	0,10265151
gir_mul_34512	19,19551085	-0,700545290238785	0,277044581	-2,5286374	0,011450626	0,105541354
gir_mul_pmca2	26,87472063	-0,72500915884648	0,288322026	-2,51458124	0,011917384	0,10725483
gir_mul_stim	64,43140684	-0,359388170872851	0,143395701	-2,5062688	0,012201282	0,108913152
gir_mul_27566	32,98629969	0,64946421950247	0,259330364	2,504389419	0,012266294	0,108913152
gir_mul_pctaire	19,35472014	-0,648066193962536	0,261208192	-2,48103319	0,013100217	0,113969487
gir_mul_cdkl1	10,18760995	-0,918063991694543	0,373249321	-2,45965348	0,013907122	0,116567785
gir_mul_fzdP-1	54,73531804	-0,682322677062008	0,2876097	-2,37239105	0,017673375	0,128940958
gir_mul_33093	119,3086231	-0,60597346800529	0,266274582	-2,27574657	0,022861182	0,144866431
gir_mul_pde1c	27,66840604	-0,786882423867081	0,357174852	-2,20307342	0,027589574	0,155344632

gir_mul_tph	9,485932259	-0,853799004260926	0,38853816	-2,19746499	0,027987254	0,156222293
gir_mul_08086	93,03838302	-0,343069569360041	0,156120667	-2,19746415	0,027987314	0,156222293
gir_mul_12518	8,087303117	-1,12798484917682	0,513638842	-2,1960661	0,028087212	0,156442753
gir_mul_abl	73,86644911	-0,446584390547316	0,204192718	-2,18707305	0,028737192	0,157158894
gir_mul_17028	16,54521067	-0,884816603869994	0,415340349	-2,13034107	0,033143465	0,166682668
gir_mul_03464	7,121309453	-0,774789675623864	0,364017127	-2,12844291	0,033300378	0,166870508
gir_mul_09627	45,32793507	-0,457288053170925	0,217167804	-2,10568991	0,035231297	0,172875955
gir_mul_inpp5	50,13152177	-0,376030970415714	0,181418478	-2,07272696	0,038197697	0,179646946
gir_mul_13049	72,43506592	0,529832460191849	0,262834695	2,015839123	0,043816801	0,194324511
gir_mul_04906	39,00551614	-0,612817940729681	0,310865195	-1,97133018	0,048686121	0,206716483
gir_mul_28813	6,791216408	0,961453103171823	0,490567297	1,959880142	0,050009801	0,209588001
gir_mul_ddc	13,73949574	-0,873205795342197	0,455564606	-1,91675513	0,055269048	0,221589525
gir_mul_07357	60,81897964	-0,409235877204884	0,215336316	-1,90044988	0,057374106	0,225261604
gir_mul_03129	25,8173754	-0,405904152783197	0,222284659	-1,82605563	0,067841883	0,245648382
gir_mul_20798	23,94782466	0,456041091719155	0,252384017	1,806933331	0,070772675	0,249050581
gir_mul_09720	13,39080554	-0,729138769339342	0,432789746	-1,68474132	0,09203849	0,289999622
gir_mul_26028	195,8553885	-0,312393053727884	0,185807549	-1,68127213	0,092710069	0,291406649
gir_mul_rgs6-7-9	261,891821	-0,393486471074783	0,235638641	-1,66987243	0,094944605	0,294146564
gir_mul_hcng	26,45552489	-0,510612923133118	0,306344347	-1,66679401	0,095555371	0,295288171
gir_mul_trpc-2	41,50356115	-0,32500226148492	0,197880563	-1,6424163	0,100503759	0,304006849
gir_mul_20488	32,04060628	-0,433820528563097	0,266058336	-1,63054665	0,102986014	0,308760446
gir_mul_nhe-rf	31,18206398	0,30966326217189	0,194196755	1,594585152	0,110805035	0,3220932
gir_mul_21691	98,88415036	-0,471602768216249	0,296446849	-1,59085101	0,111643111	0,323440332
gir_mul_ip3k	31,05446574	-0,405699207318025	0,255481304	-1,58798003	0,112290856	0,324590754
gir_mul_ttbk	37,83289369	-0,444465700194048	0,281407342	-1,57943889	0,114235422	0,326567046
gir_mul_otxA	11,97420571	-0,383496740672531	0,242768641	-1,57967989	0,114180193	0,326567046
gir_mul_ncx	147,0268248	-0,267801129334036	0,171405821	-1,5623806	0,118198354	0,332475912
gir_mul_23489	26,86207329	-0,32057911863755	0,206844747	-1,54985381	0,121176609	0,33632316
gir_mul_17151	19,04892488	-0,488073189427267	0,318118235	-1,5342509	0,124967939	0,340897792
gir_mul_01362	23,64621775	-0,353660620759446	0,234776061	-1,50637428	0,131971119	0,349494067
gir_mul_14918	35,73721404	-0,468201103364477	0,320679621	-1,46002762	0,144282484	0,372945741
gir_mul_smad6-7-2	66,57565412	-0,577429095776467	0,399416659	-1,44568105	0,148266681	0,379941044
gir_mul_best-a	4,649296593	0,697068918905947	0,486569801	1,432618542	0,15196688	0,385498745
gir_mul_arrestin	91,59578752	-0,538647453996702	0,385403879	-1,39761814	0,162227767	0,401308422
gir_mul_cdh23	13,5312933	0,428169787535769	0,336297417	1,273187857	0,202951388	0,455017221
gir_mul_gpcrP-2	12,03023699	-0,446002889631654	0,355191455	-1,25566897	0,209236013	0,46173299
gir_mul_23388	83,84837155	-0,161924752522717	0,13169036	-1,22958698	0,218851807	0,473143722
gir_mul_ovo	8,205386898	-0,47138508332608	0,438597273	-1,07475607	0,282483951	0,546675553
gir_mul_myoVI	85,22818206	0,277945252648985	0,259626052	1,070559948	0,284367341	0,547999563
gir_mul_02471	14,57968834	0,251109912269821	0,243052204	1,033152171	0,301532691	0,569635063
gir_mul_01915	57,87718834	-0,149213758856177	0,145560448	-1,02509824	0,305316835	0,573612711
gir_mul_ip3r	22,83690887	-0,328454628198542	0,327579429	-1,00267171	0,316019282	0,583548121
gir_mul_plcb	23,56483348	-0,462069700161015	0,479570952	-0,96350644	0,335293435	0,600560163
gir_mul_gna-q	116,2821222	-0,302396323115961	0,317719955	-0,95177001	0,341213637	0,603224209
gir_mul_08883	40,4561405	0,177769764057194	0,196896905	0,902857075	0,366601755	0,62768096
gir_mul_19866	77,47210417	0,262469741101235	0,291166812	0,901441134	0,367353819	0,62768096
gir_mul_arrb-2	8,11472171	-0,360771173487754	0,405786159	-0,88906722	0,373966954	0,63274417
gir_mul_10513	78,53833905	-0,126687178999873	0,143000141	-0,88592345	0,375658776	0,633435046

gir_mul_23244	44,99460518	-0,162986139789453	0,191025826	-0,85321521	0,39353997	0,646866231
gir_mul_robo-c	146,8565539	-0,180964460772134	0,242020515	-0,74772364	0,454626869	0,694202571
gir_mul_six-1-2	21,49392696	-0,30234753036477	0,410449107	-0,73662611	0,46134975	0,697953186
gir_mul_pis	16,12499518	-0,137357646916587	0,194153647	-0,7074688	0,479275198	0,712175997
gir_mul_dagk	41,76232737	-0,141333774408906	0,217539039	-0,64969384	0,515890002	0,74148452
gir_mul_06952	21,41095333	0,173427972702226	0,269075984	0,644531594	0,519230777	0,745045824
gir_mul_cds	45,24490868	-0,105279921615773	0,175772126	-0,59895686	0,54920165	0,762579397
gir_mul_dlx	13,28359767	-0,16223307897883	0,285508867	-0,56822431	0,569882666	0,772750112
gir_mul_09274	37,41386113	-0,175313242620376	0,309827241	-0,56584193	0,571501243	0,772944239
gir_mul_foxq2	8,587303355	-0,206616903356341	0,393405153	-0,52520131	0,599443252	0,79374132
gir_mul_05359	35,93096623	0,0976115077709606	0,213315455	0,457592291	0,647245383	0,813663741
gir_mul_myoVIIA	258,7886965	0,163408107332692	0,378611707	0,431598136	0,666033513	0,824126251
gir_mul_crf-r	8,986567158	0,160553909730284	0,3761407	0,426845352	0,669491971	0,824326197
gir_mul_10538	10,51662923	-0,138973336424189	0,346944205	-0,40056394	0,688741202	0,835913643
gir_mul_04256	18,22760366	0,0933948122169766	0,236473793	0,394947834	0,692881387	0,839657846
gir_mul_03496	98,30938264	0,0792156993923352	0,203180303	0,389878834	0,696626146	0,841594008
gir_mul_07503	40,92741355	-0,050100088704783	0,138584828	-0,36151207	0,717716681	0,848589473
gir_mul_34343	20,43704029	-0,090787291872129	0,279023773	-0,32537476	0,744897459	0,863600904
gir_mul_atp13	57,07261499	0,078650312494884	0,275057998	0,285940831	0,77492344	0,878744182
gir_mul_sans	6,505450923	-0,144662613223153	0,513016418	-0,28198437	0,777955492	0,879871059
gir_mul_unc5-2	74,39671998	0,0505821881074856	0,194037781	0,260682162	0,794337624	0,890187404
gir_mul_fzdP-2	207,7698584	-0,034541974703329	0,221434555	-0,1559918	0,876039485	0,944212346
gir_mul_egfr-1	29,77638411	-0,043727849828038	0,331125011	-0,13205843	0,894938074	0,951124174
gir_mul_erm	7,974600663	-0,055275244302512	0,442541673	-0,12490404	0,900599517	0,953229567
gir_mul_00978	103,5848499	0,0253730176214565	0,209991157	0,120828981	0,903826494	0,954305186
gir_mul_30263	14,31510384	0,0174450658220385	0,273973855	0,063674199	0,949229649	0,974051027
gir_mul_unc5-1	92,916547	0,0163211482711712	0,292301992	0,055836596	0,955471981	0,978905234
gir_mul_rops2	28,73048798	-0,01547953836226	0,293136001	-0,05280668	0,957885941	0,980602603
gir_mul_31312	2,571412387	-1,435385591616	0,528313321	-2,71692107	0,006589232	NA
gir_mul_5ht-r2	1,405958211	-1,27657344110444	0,668090725	-1,91077857	0,056033043	NA
gir_mul_best-b	1,445315053	-1,19678897820336	0,676575263	-1,7688926	0,076911799	NA
gir_mul_eag	1,930413554	-1,04226729558121	0,65913814	-1,58125775	0,113819115	NA
gir_mul_gucy-2	4,027105906	-0,94980495516137	0,439771033	-2,15977152	0,030790361	NA
gir_mul_ninag	2,165369495	-0,761074062692243	0,624846924	-1,21801682	0,223217581	NA
gir_mul_22239	4,240498188	-0,750133754021415	0,468805344	-1,60009642	0,109577194	NA
gir_mul_cpo	1,275200168	-0,693785113117173	0,684051037	-1,01423004	0,310472996	NA
gir_mul_34307	3,913674276	-0,610588995316409	0,402589919	-1,51665247	0,12935445	NA
gir_mul_nkx1	3,441672541	-0,592653831593183	0,413126	-1,43455951	0,15141266	NA
gir_mul_transferrin	4,298065723	-0,304721139479848	0,530351448	-0,57456455	0,565585835	NA
gir_mul_cng	3,612864606	-0,205563521055484	0,462917671	-0,44406065	0,656998736	NA
gir_mul_22592	1,331574395	0,0021833209218344	0,679538047	0,003212949	0,997436442	NA
gir_mul_nol1	3,818123629	0,0941450661372704	0,431146064	0,218360027	0,827148608	NA
gir_mul_klf	3,500491823	0,102773517547348	0,576616365	0,17823552	0,858538016	NA
gir_mul_sp6-9	1,432347217	0,142228936779413	0,609583775	0,233321395	0,815511846	NA
gir_mul_23802	1,30360065	0,632828475720041	0,695720318	0,909601832	0,363032532	NA
gir_mul_gls	4,156605037	0,70006688562508	0,481018548	1,455384389	0,145562876	NA
gir_mul_nanos	1,431419879	0,975819584326934	0,641751561	1,520556619	0,12837114	NA
gir_mul_07486	4,304660279	1,08805032034256	0,47384192	2,296230608	0,021662699	NA

Table S3b. Results of the differential gene expression analysis in planarian eye-related gene orthologs. DESeq2 Wald test pair-wise comparison between *G. dorotocephala* and *G. multidiverticulata* morphotypes.

***G. dorotocephala and G. multidiverticulata* non-discernible eyes:**

gene	baseMean	log2FoldChan	lfcSE	stat	pvalue	padj
gir_mul_tyrosinase	112,4774342	-4,77501228	0,444914936	-10,7324162	7,17E-27	3,49E-25
gir_mul_tph	55,73708718	-4,10615923	0,418724091	-9,80636013	1,06E-22	3,45E-21
gir_mul_19866	47,46845961	3,049772023	0,336368226	9,066766074	1,23E-19	2,99E-18
gir_mul_rops2	75,84765669	-2,26119462	0,305977784	-7,39006144	1,47E-13	1,89E-12
gir_mul_arrestin	296,069815	-2,93283929	0,418957516	-7,00032624	2,55E-12	2,86E-11
gir_mul_16656	28,89609572	-2,10114472	0,304751319	-6,89462061	5,40E-12	5,68E-11
gir_mul_fzdP-1	88,67898437	-1,81274992	0,267743983	-6,77045998	1,28E-11	1,31E-10
gir_mul_12518	13,95022965	-2,25340272	0,346343686	-6,50626186	7,70E-11	7,00E-10
gir_mul_03129	36,41258749	-1,27639384	0,212499151	-6,00658327	1,89E-09	1,45E-08
gir_mul_cpo	5,759809143	-3,51947088	0,61487692	-5,7238624	1,04E-08	7,18E-08
gir_mul_inpp5	71,11817057	-1,34854068	0,236118284	-5,71129289	1,12E-08	7,68E-08
gir_mul_pmca2	36,01419709	-1,40374062	0,24651573	-5,69432472	1,24E-08	8,41E-08
gir_mul_09132	21,04159714	-1,64112498	0,288622021	-5,68606989	1,30E-08	8,75E-08
gir_mul_cgs-pde	25,76063288	-1,39188723	0,251493266	-5,53449105	3,12E-08	2,00E-07
gir_mul_01362	39,80717903	-1,62496883	0,302864576	-5,36533142	8,08E-08	4,84E-07
gir_mul_22592	3,918965352	-2,6979258	0,531071245	-5,08015793	3,77E-07	2,04E-06
gir_mul_cds	69,24219844	-1,23781764	0,244671108	-5,0591083	4,21E-07	2,27E-06
gir_mul_gpcrP-1	16,78622457	-1,40551746	0,277820942	-5,05907673	4,21E-07	2,27E-06
gir_mul_pitp	28,39927046	-1,61969713	0,321646213	-5,03564806	4,76E-07	2,54E-06
gir_mul_09268	89,54986664	-1,02666716	0,204441418	-5,02181587	5,12E-07	2,72E-06
gir_mul_34307	8,832647734	-2,10584538	0,423331153	-4,97446352	6,54E-07	3,40E-06
gir_mul_gna-o	102,221816	-1,67824366	0,342233462	-4,9037977	9,40E-07	4,81E-06
gir_mul_22239	8,798759418	-2,22201498	0,455412805	-4,87912276	1,07E-06	5,41E-06
gir_mul_27330	50,69559742	-0,97207709	0,210695327	-4,61366232	3,96E-06	1,80E-05
gir_mul_cdkl1	11,60318247	-1,3606098	0,30154007	-4,51220229	6,42E-06	2,81E-05
gir_mul_myoVIIA	175,2273354	1,568769905	0,365048041	4,297434112	1,73E-05	6,93E-05
gir_mul_29449	15,11552662	-1,80517202	0,420173	-4,29625897	1,74E-05	6,95E-05
gir_mul_pcdhP-4	56,85397821	-1,01740762	0,241998117	-4,20419642	2,62E-05	0,000101434
gir_mul_crf-r	5,866485834	1,834471869	0,439322427	4,175684542	2,97E-05	0,000113866
gir_mul_09720	21,25980949	-1,83789605	0,448947148	-4,09379157	4,24E-05	0,000158303
gir_mul_23388	93,51298959	-0,62353273	0,152765162	-4,08164218	4,47E-05	0,000165604
gir_mul_04136	8,477124906	1,67495379	0,421565729	3,973173521	7,09E-05	0,000252001
gir_mul_unc5-1	66,26127249	1,03454664	0,261412364	3,957527574	7,57E-05	0,000268154
gir_mul_cng	5,60588631	-1,45346441	0,373828564	-3,88805069	0,000101053	0,000347015
gir_mul_10513	93,02695134	-0,72534816	0,188735285	-3,84320375	0,000121439	0,000410139
gir_mul_29424	151,7063523	-0,79000381	0,206291739	-3,82954651	0,00012838	0,000431209
gir_mul_eya	19,21034361	-1,19548877	0,315849524	-3,78499468	0,000153712	0,000508116
gir_mul_32587	12,85795801	-1,16016964	0,330296963	-3,51250472	0,000443904	0,001326958
gir_mul_transferrin	6,654633361	-1,6498378	0,473687462	-3,482967	0,000495889	0,001470899
gir_mul_trpc-2	54,35378032	-1,06764204	0,310467137	-3,43882463	0,000584246	0,001703356
gir_mul_09627	46,21515826	-0,63699679	0,189151109	-3,36766086	0,000758088	0,002158933

gir_mul_rgs6-7-9	292,2476383	-0,80823147	0,241768991	-3,34299063	0,000828807	0,002334346
gir_mul_maguk	229,7121132	-0,56704362	0,174718349	-3,24547262	0,001172558	0,003188088
gir_mul_pctaire	22,26216855	-1,08851195	0,335359115	-3,24580993	0,00117117	0,003188088
gir_mul_ddryk	31,89022341	-0,87082139	0,27445015	-3,17296745	0,001508894	0,003979644
gir_mul_17028	15,82143467	-0,97742113	0,315578006	-3,09724096	0,00195331	0,004997219
gir_mul_27566	30,85049016	0,792165245	0,256555495	3,087695493	0,00201715	0,005143399
gir_mul_nhe-rf	24,97394906	1,040283917	0,342054996	3,041276784	0,002355772	0,005899037
gir_mul_ninag	3,605479088	-1,63452104	0,555798941	-2,9408495	0,003273135	0,008006793
gir_mul_ddc	14,61973258	-1,16269153	0,417946753	-2,78191305	0,005403952	0,012520854
gir_mul_sk3	36,72241151	-0,63479558	0,228947538	-2,77266831	0,005559876	0,012843355
gir_mul_ip3k	33,00379082	-0,74719659	0,269798062	-2,76946612	0,005614824	0,012960534
gir_mul_31312	2,409245564	-1,5287914	0,55709902	-2,74420049	0,006065848	0,013924306
gir_mul_nkx1	3,978612652	-1,02145992	0,377912436	-2,70290105	0,006873721	0,015470911
gir_mul_nol1	2,500405485	1,381935589	0,513360594	2,691939359	0,007103786	0,015895498
gir_mul_gls	8,680143502	-1,30073873	0,488139207	-2,66468809	0,007705976	0,017118196
gir_mul_21613	167,7348833	-0,58309002	0,218971188	-2,66286186	0,007747921	0,017186502
gir_mul_ncx	162,7553333	-0,68155644	0,25995663	-2,62180826	0,008746462	0,019179742
gir_mul_08883	50,96423876	-0,65882795	0,251486755	-2,61973221	0,008799884	0,019283114
gir_mul_smad6-7-2	74,57085652	-0,95963383	0,36654892	-2,6180239	0,008844061	0,01933851
gir_mul_08086	90,64254366	-0,4345095	0,174327212	-2,49249384	0,012684953	0,026764815
gir_mul_sans	10,24629848	-1,16963531	0,474220349	-2,46643847	0,013646417	0,028596929
gir_mul_07357	59,32847489	-0,50809747	0,20839927	-2,43809623	0,014764843	0,030772619
gir_mul_14918	36,06848451	-0,64183902	0,265703563	-2,41562068	0,015708416	0,032518434
gir_mul_21691	107,2556377	-0,79620823	0,338940753	-2,34910739	0,018818478	0,038033396
gir_mul_sp6-9	2,379398191	-1,28173676	0,560266995	-2,28772491	0,022153546	0,044048825
gir_mul_23802	0,848331127	1,992387628	0,879420429	2,265568962	0,023477787	0,046262392
gir_mul_04906	39,80685891	-0,73806216	0,33388913	-2,21050072	0,027070431	0,052235212
gir_mul_23244	47,13006435	-0,41880704	0,19262505	-2,17420858	0,029689474	0,056789212
gir_mul_25626	154,3533135	-0,63263382	0,296538815	-2,13339295	0,032892505	0,061912931
gir_mul_stim	63,87786679	-0,43668372	0,206664111	-2,11301187	0,034599752	0,064729578
gir_mul_59sley	5,847828317	-0,84789087	0,405404219	-2,09147026	0,03648593	0,067762737
gir_mul_09274	27,48428648	0,705837869	0,347638344	2,030379795	0,042317949	0,077055814
gir_mul_05359	40,1249141	-0,35393208	0,181548576	-1,94951726	0,051233684	0,091022806
gir_mul_12199	97,29323274	-0,44749203	0,244076393	-1,8334097	0,066741667	0,114403639
gir_mul_dlx	17,27390243	-0,69970913	0,38449344	-1,81982073	0,06878631	0,117318873
gir_mul_hcng	25,76689858	-0,58496418	0,322920309	-1,81148154	0,070066345	0,119039114
gir_mul_eag	1,822558431	-1,20848854	0,67698135	-1,78511349	0,07424291	0,125509766
gir_mul_best-b	1,396772808	-1,31815163	0,758801387	-1,73714974	0,082360744	0,137119025
gir_mul_best-a	7,021974088	-0,79462837	0,471429815	-1,68557088	0,091878483	0,150596339
gir_mul_32930	28,10133763	-0,3836301	0,228418214	-1,67950746	0,093053185	0,152277867
gir_mul_arrb-2	8,402424873	-0,61443536	0,366817061	-1,67504576	0,093925255	0,153507715
gir_mul_pde1c	16,42284824	0,672869343	0,403605797	1,667144891	0,095485598	0,15574364
gir_mul_ovo	9,457189859	-0,71635275	0,439764405	-1,62894665	0,103324314	0,164926611
gir_mul_egfr-1	32,76987369	-0,48554495	0,301477144	-1,61055309	0,107277165	0,170202013
gir_mul_unc5-2	65,12510537	0,296748079	0,1892772	1,567796222	0,116928697	0,182868619
gir_mul_10538	11,04953621	-0,5337867	0,353413358	-1,510375	0,130947764	0,200703762

gir_mul_fzdP-2	172,4968065	0,376267617	0,249567344	1,507679694	0,131636525	0,201457693
gir_mul_04256	15,6494416	0,406184766	0,269723608	1,505929605	0,132085246	0,20194308
gir_mul_26028	150,602507	0,294498527	0,196295187	1,500283996	0,133540853	0,203762634
gir_mul_gna-q	84,89992158	0,516469923	0,34724345	1,487342446	0,136924389	0,20778936
gir_mul_foxq2	11,73103848	-0,6956152	0,507189393	-1,37150975	0,170216107	0,250077903
gir_mul_5ht-r2	1,286203966	-0,96057271	0,755623209	-1,2712324	0,203645982	0,288639504
gir_mul_nf-i-a	35,78104056	-0,27239194	0,216720343	-1,25688219	0,208796298	0,294579336
gir_mul_20047	85,99766472	-0,52725769	0,420584856	-1,25362976	0,209976609	0,296108493
gir_mul_myoVI	79,82160858	0,312117997	0,251606881	1,240498654	0,214791012	0,301926926
gir_mul_klf	2,69150342	0,790847316	0,643141033	1,229663909	0,218822987	0,306611853
gir_mul_32281	311,4203365	0,45369669	0,372061498	1,21941317	0,222687414	0,310891478
gir_mul_cdh23	17,17546513	-0,39399612	0,340054594	-1,15862608	0,246608633	0,337383468
gir_mul_ttbk	27,11111601	0,439805869	0,380661458	1,155372734	0,247937823	0,338899873
gir_mul_ip3r	22,13438077	-0,37283325	0,32299921	-1,15428532	0,248383213	0,339357571
gir_mul_17151	16,94794736	-0,3197976	0,280966992	-1,13820347	0,255035532	0,34674893
gir_mul_robo-c	144,8392496	-0,29991478	0,278653958	-1,0762983	0,281793867	0,375154888
gir_mul_23489	23,58577238	-0,24040812	0,224325676	-1,0716924	0,283858215	0,377575702
gir_mul_meis	20,89553793	-0,26148129	0,247969423	-1,05449005	0,291658615	0,386611377
gir_mul_01915	49,44343609	0,200661862	0,199437573	1,006138711	0,31434885	0,412062753
gir_mul_20798	29,1236906	-0,25832028	0,257959284	-1,00139943	0,316633738	0,414173658
gir_mul_dagk	40,24801906	-0,25543659	0,257993215	-0,99009035	0,322129961	0,419931626
gir_mul_plcb	22,46367852	-0,51417638	0,5360603	-0,95917638	0,337469896	0,437329076
gir_mul_13049	88,35952214	-0,25261494	0,265850383	-0,95021468	0,34200318	0,440046003
gir_mul_gucy-2	2,173058761	0,483229672	0,54076853	0,893597992	0,371537021	0,471915041
gir_mul_nanos	1,360322452	0,556215306	0,659457136	0,843444215	0,398980052	0,499024055
gir_mul_26962	115,6332412	0,211859692	0,254186029	0,833482837	0,404572473	0,505098615
gir_mul_erm	8,145789183	-0,35000344	0,439326193	-0,7966824	0,42563551	0,526047108
gir_mul_six-1-2	21,10080952	-0,32684911	0,412853945	-0,79168217	0,428546024	0,529218139
gir_mul_06952	23,02583081	-0,20675874	0,26423372	-0,7824843	0,433930007	0,534995956
gir_mul_30263	14,23968468	-0,24151422	0,31272608	-0,7722868	0,439944584	0,540468137
gir_mul_11992	12,54031559	0,212589211	0,288683967	0,736408098	0,461482378	0,560423615
gir_mul_13405	59,35599484	-0,25451697	0,358223332	-0,71049802	0,477395358	0,575649548
gir_mul_otxA	10,31612337	-0,18342649	0,283015507	-0,64811463	0,516910809	0,613182451
gir_mul_07486	5,636368861	0,307798468	0,500609544	0,614847383	0,538655514	0,633590968
gir_mul_15984	64,41650021	-0,10356127	0,171358453	-0,60435459	0,545607922	0,640786656
gir_mul_28813	8,150949687	0,237798534	0,447705259	0,531149745	0,595315015	0,689146718
gir_mul_abl	57,41396196	0,123434692	0,314527093	0,392445342	0,69472919	0,776134866
gir_mul_03496	94,15257145	0,072970092	0,216515183	0,337020669	0,736101319	0,810847166
gir_mul_07503	38,23781998	-0,06062619	0,188426713	-0,32174944	0,747642521	0,819151513
gir_mul_00978	96,65075716	0,062041749	0,218931048	0,283384882	0,776881816	0,843361802
gir_mul_08711	24,93273421	0,118254533	0,419224269	0,282079406	0,777882624	0,843851469
gir_mul_pis	15,13068354	-0,07202397	0,268925775	-0,26782102	0,788837094	0,853193217
gir_mul_02471	14,51601671	-0,07224272	0,273332859	-0,26430308	0,791546384	0,85474759
gir_mul_20488	26,19068047	-0,06135747	0,241225509	-0,25435731	0,799219529	0,861215849
gir_mul_03464	4,683731774	0,100451126	0,410196138	0,244885597	0,806545014	0,86728308
gir_mul_qdr	95,5297225	-0,05743894	0,373077128	-0,15395996	0,877641308	0,920204514

gir_mul_34343	20,18041912	-0,04563417	0,337402908	-0,13525126	0,89241322	0,926906678
gir_mul_gpcrP-2	9,65409569	0,048357998	0,387074448	0,124932034	0,900577357	0,931841081
gir_mul_33093	88,16811051	0,03171192	0,264506163	0,119891045	0,904569458	0,9345332
gir_mul_34512	14,01586457	-0,0251769	0,282608414	-0,08908759	0,929012303	0,951804464
gir_mul_atp13	54,95066837	0,017251713	0,284744629	0,060586613	0,951688435	0,96520763
gir_mul_5ht-r1	21,51852102	-0,01058288	0,273818436	-0,03864926	0,969170028	0,977769302
gir_mul_soxB	10,67290879	-0,00045288	0,387967178	-0,00116733	0,999068608	0,999394144

***G. dorotocephala and G. multidiverticulata* discernible eyes:**

gene	baseMean	log2FoldChan	lfcSE	stat	pvalue	padj
gir_mul_04136	13,94292517	2,44819314	0,295289297	8,29082926	1,12E-16	1,74E-15
gir_mul_19866	43,70758208	2,741251787	0,338847811	8,089920301	5,97E-16	8,49E-15
gir_mul_tph	64,88945921	-3,28402951	0,430947796	-7,62048105	2,53E-14	3,09E-13
gir_mul_rops2	84,84196167	-2,2694715	0,327876306	-6,92173073	4,46E-12	4,32E-11
gir_mul_qdr	75,48092825	-1,28669327	0,187893803	-6,84798142	7,49E-12	7,06E-11
gir_mul_arrestin	347,3920523	-2,42936185	0,391295855	-6,20850393	5,35E-10	4,00E-09
gir_mul_cpo	6,459748121	-2,84042786	0,525226872	-5,40800177	6,37E-08	3,67E-07
gir_mul_22592	4,071730928	-2,67376832	0,49518109	-5,3995768	6,68E-08	3,82E-07
gir_mul_11992	22,25320576	1,317833445	0,24590633	5,359087109	8,36E-08	4,71E-07
gir_mul_03129	43,4589205	-0,89386095	0,179011439	-4,99331749	5,94E-07	3,02E-06
gir_mul_cds	78,12145641	-1,16965008	0,236429074	-4,94714995	7,53E-07	3,71E-06
gir_mul_gls	8,200998632	-2,0506831	0,420737928	-4,8740153	1,09E-06	5,24E-06
gir_mul_fzdP-1	111,2944466	-1,17183331	0,245166842	-4,77973818	1,76E-06	8,08E-06
gir_mul_inpp5	85,70811436	-1,00744472	0,212304732	-4,74527679	2,08E-06	9,45E-06
gir_mul_01362	46,8130239	-1,33070456	0,282242692	-4,71475292	2,42E-06	1,09E-05
gir_mul_tyrosinase	146,1517042	-2,36491153	0,535045195	-4,42002199	9,87E-06	3,94E-05
gir_mul_16656	39,46340309	-0,99246896	0,235582169	-4,21283566	2,52E-05	9,40E-05
gir_mul_myoVIIA	179,0433927	1,394873929	0,3310985	4,21286696	2,52E-05	9,40E-05
gir_mul_08711	16,44572074	-1,71670774	0,425974828	-4,0300685	5,58E-05	0,000195099
gir_mul_34307	10,68155276	-1,54499097	0,389250945	-3,96913866	7,21E-05	0,000248144
gir_mul_22239	10,99417548	-1,48066718	0,374664596	-3,9519805	7,75E-05	0,000263678
gir_mul_crf-r	5,601762814	1,607511553	0,408681108	3,933412926	8,37E-05	0,000282099
gir_mul_pde1c	24,98385572	1,401249343	0,359046245	3,902698781	9,51E-05	0,000316606
gir_mul_5ht-r1	34,99749451	0,914585666	0,23552542	3,883171787	0,000103103	0,000340206
gir_mul_10513	106,1414707	-0,63658808	0,166328012	-3,82730529	0,000129554	0,000417617
gir_mul_08883	53,54966802	-0,87454842	0,232494201	-3,76159243	0,000168835	0,000534152
gir_mul_23388	107,806214	-0,50851102	0,13597079	-3,73985485	0,000184127	0,000578953
gir_mul_best-a	6,703478115	-1,53574241	0,419287406	-3,66274394	0,000249528	0,000762736
gir_mul_20798	27,69943725	-0,75711572	0,206900803	-3,65931746	0,000252888	0,000770706
gir_mul_unc5-1	72,5804541	0,996733668	0,277608494	3,590429293	0,000330134	0,000984632
gir_mul_gna-o	135,9811237	-0,83056836	0,232687078	-3,56946491	0,000357711	0,001061728
gir_mul_32281	521,235676	1,335089276	0,376250377	3,548406477	0,00038757	0,001139343
gir_mul_13049	83,29200698	-0,810951	0,229891934	-3,52753133	0,000419454	0,001223707
gir_mul_soxB	17,84435378	0,996014732	0,283507589	3,513185435	0,000442768	0,001283193
gir_mul_gna-q	105,8508397	0,78870635	0,225181005	3,502543878	0,000460838	0,001328043
gir_mul_26028	189,0279401	0,57828034	0,165623847	3,491528252	0,000480266	0,001377569

gir_mul_12518	18,49773521	-1,1445228	0,338160757	-3,3845524	0,000712944	0,001962513
gir_mul_gucy-2	3,830481087	1,395950779	0,414757345	3,365704784	0,000763484	0,002079275
gir_mul_09274	32,08120777	0,826197266	0,262462927	3,147862736	0,001644689	0,004179061
gir_mul_cng	6,385193201	-1,28117912	0,416352613	-3,07714923	0,002089906	0,005173403
gir_mul_26962	162,6310049	0,777078112	0,254524515	3,053058017	0,002265221	0,005571887
gir_mul_09268	113,4225435	-0,55402282	0,181673147	-3,04955814	0,002291783	0,005627783
gir_mul_pitp	38,36682044	-0,75135372	0,249851404	-3,0072023	0,002636642	0,00637275
gir_mul_sp6-9	2,664642909	-1,46945403	0,511405975	-2,87336109	0,004061296	0,009379481
gir_mul_33093	123,1140175	0,603744043	0,210785823	2,864253558	0,004179933	0,009611342
gir_mul_pmca2	46,71942812	-0,73711506	0,272529674	-2,70471486	0,006836304	0,014947421
gir_mul_transferrin	7,955793156	-1,33869324	0,497831607	-2,6890483	0,007165605	0,015578725
gir_mul_trpc-2	64,67908628	-0,79249688	0,295737182	-2,67973366	0,007368076	0,015996275
gir_mul_sans	11,62966071	-1,07588918	0,421163316	-2,55456527	0,010632049	0,022198319
gir_mul_ttbk	35,6946071	0,853054169	0,333951159	2,554427932	0,010636245	0,022198319
gir_mul_09720	27,02989638	-1,14656705	0,453931653	-2,52585834	0,011541601	0,023859891
gir_mul_09132	28,00386653	-0,80520275	0,319835845	-2,51755005	0,011817418	0,024364502
gir_mul_cdh23	16,8344357	-0,8532283	0,340725905	-2,50414861	0,012274647	0,025155246
gir_mul_15984	87,90191324	0,44313132	0,17982254	2,464270171	0,013729258	0,027820766
gir_mul_34512	19,33807988	0,627002477	0,259654049	2,414761021	0,015745537	0,03130763
gir_mul_05359	43,12811811	-0,50185639	0,212263474	-2,36430874	0,018063752	0,035435405
gir_mul_32930	43,1312129	0,484894045	0,20725195	2,33963562	0,019302562	0,03760144
gir_mul_28813	6,538550564	-0,76072487	0,327029453	-2,32616624	0,020009684	0,038757723
gir_mul_nol1	2,620450149	1,276244143	0,554252305	2,30264111	0,021299044	0,04102236
gir_mul_03464	6,991374084	0,837374052	0,374841711	2,233940423	0,025486993	0,047770618
gir_mul_12199	146,8228022	0,413216457	0,189034362	2,185933031	0,028820505	0,053047688
gir_mul_29449	20,39063465	-0,8172286	0,384195606	-2,12711595	0,033410451	0,059986502
gir_mul_eya	24,95820126	-0,57548608	0,272510592	-2,11179345	0,034704169	0,061947244
gir_mul_nhe-rf	23,1505251	0,663582905	0,314383967	2,11074029	0,034794641	0,06207267
gir_mul_27330	65,77791426	-0,43619416	0,213574555	-2,04235079	0,041116747	0,071685952
gir_mul_nf-i-a	50,56314138	0,364442614	0,182555768	1,996335796	0,045897384	0,078901379
gir_mul_abl	75,27433906	0,54468831	0,277506709	1,962793306	0,049670196	0,084395377
gir_mul_06952	23,74730839	-0,42492967	0,223192685	-1,90386915	0,056927228	0,095302694
gir_mul_ip3k	41,69144345	-0,37815545	0,199422008	-1,89625735	0,057926023	0,096763862
gir_mul_07486	4,274838013	-0,81224735	0,432932796	-1,87615112	0,060634532	0,101068521
gir_mul_ncx	194,2079203	-0,44432935	0,237708239	-1,86922148	0,061592006	0,102331338
gir_mul_rgs6-7-9	359,140319	-0,45966039	0,24666453	-1,8635042	0,062391362	0,103379862
gir_mul_13405	91,69373659	0,610913757	0,328882923	1,857541737	0,063234119	0,104607008
gir_mul_pctaire	28,59500337	-0,49380349	0,269798799	-1,83026572	0,067210215	0,110175976
gir_mul_fzdP-2	192,0955765	0,377108118	0,21405648	1,761722502	0,078116194	0,126235112
gir_mul_23244	55,01605668	-0,2811479	0,15966615	-1,76084851	0,078264047	0,126407546
gir_mul_01915	57,51048304	0,316283302	0,183886457	1,719992366	0,085433829	0,136622969
gir_mul_gpcrP-1	23,45039359	-0,47716477	0,286824656	-1,66361142	0,096190109	0,151847901
gir_mul_meis	28,52151007	0,307762321	0,188721013	1,630779291	0,102936899	0,160927304
gir_mul_ninag	4,579849474	-0,95195791	0,599208437	-1,58869244	0,112129847	0,173358273
gir_mul_32587	16,66543464	-0,422109	0,265834628	-1,5878631	0,112317298	0,173560735
gir_mul_smad6-7-2	96,02888218	-0,44632003	0,283880486	-1,57221102	0,115901621	0,177669551

gir_mul_23802	0,768482045	1,39689047	0,894579275	1,561505514	0,118404524	0,181325373
gir_mul_dlx	19,80913071	-0,54657309	0,353726342	-1,54518627	0,122301215	0,186641496
gir_mul_20488	33,44378261	0,340127151	0,22636669	1,502549475	0,132955261	0,201010082
gir_mul_cdkl1	15,97460759	-0,47831962	0,32217211	-1,48467109	0,137630972	0,207154506
gir_mul_02471	15,02472104	-0,36560383	0,247996974	-1,47422698	0,140420488	0,210322642
gir_mul_59sley	9,540440078	0,502195656	0,34762007	1,444668185	0,148551111	0,220565013
gir_mul_gpcrP-2	11,77838605	0,455446964	0,316423891	1,439357065	0,150049382	0,222252508
gir_mul_21691	134,9420468	-0,36103345	0,251729117	-1,43421411	0,15151117	0,223985715
gir_mul_klf	2,992194309	0,720426467	0,517590877	1,391884012	0,163957524	0,239276729
gir_mul_10538	12,70332479	-0,43351603	0,314801326	-1,37710992	0,168478266	0,244905345
gir_mul_egfr-1	37,31292413	-0,45529161	0,333667598	-1,3645065	0,172408209	0,249711465
gir_mul_nkx1	5,035271967	-0,46233175	0,347267373	-1,33134231	0,183076402	0,263054586
gir_mul_cgs-pde	37,78374275	-0,28994172	0,219148581	-1,32303717	0,185823018	0,266288281
gir_mul_25626	240,2492131	0,346239389	0,274725365	1,26031096	0,207557208	0,291814985
gir_mul_30263	15,61800137	-0,30250675	0,26240367	-1,15282971	0,248980287	0,341154077
gir_mul_04256	16,77626924	0,283640002	0,247270188	1,147085314	0,251346363	0,343782737
gir_mul_unc5-2	70,2448261	0,213761195	0,189583681	1,127529512	0,25951867	0,353387125
gir_mul_erm	9,144155539	-0,40001406	0,360922168	-1,10831115	0,267727464	0,362157098
gir_mul_09627	58,13128183	-0,22197322	0,201032185	-1,10416756	0,26952046	0,363791902
gir_mul_foxq2	13,92670752	-0,39961191	0,431451359	-0,92620385	0,354340051	0,4552625
gir_mul_pcdhP-4	78,15121231	-0,24909158	0,273357944	-0,91122863	0,362174924	0,463970545
gir_mul_arrb-2	9,92108061	-0,2945209	0,340358751	-0,86532489	0,386860494	0,490480989
gir_mul_20047	128,8591727	0,338739108	0,39356277	0,860699063	0,389403809	0,493298351
gir_mul_29424	202,6166559	-0,19434755	0,231469966	-0,83962317	0,401119706	0,503571613
gir_mul_ddc	20,77202477	-0,32828875	0,396737656	-0,8274706	0,407970378	0,510503056
gir_mul_08086	111,4014671	-0,12656532	0,160465953	-0,78873626	0,430266168	0,532116614
gir_mul_14918	45,47439505	-0,20773989	0,263634796	-0,78798359	0,430706306	0,532311113
gir_mul_robo-c	169,4709679	-0,14783067	0,20930283	-0,70630037	0,480001364	0,581911678
gir_mul_dagk	46,90692541	-0,14575432	0,208771659	-0,69815185	0,485082235	0,586682136
gir_mul_stim	78,09462968	-0,12671389	0,185375722	-0,68355168	0,494258304	0,595435886
gir_mul_nanos	1,022135205	-0,46692952	0,692579456	-0,67418909	0,500191102	0,601639415
gir_mul_sk3	47,7433085	-0,14129458	0,216103909	-0,65382706	0,513223232	0,612518169
gir_mul_ovo	12,17927142	-0,28129083	0,431096204	-0,6525013	0,514077835	0,613142001
gir_mul_07357	73,09505421	-0,14010025	0,21496033	-0,65174934	0,514562885	0,613402089
gir_mul_otxA	12,97101867	0,150808594	0,237348316	0,635389358	0,525174507	0,621469987
gir_mul_eag	2,59933617	-0,23648937	0,446715987	-0,52939537	0,596531212	0,684967567
gir_mul_04906	52,23444444	-0,16369499	0,310338911	-0,5274717	0,597866062	0,685570938
gir_mul_atp13	59,33096873	-0,09201678	0,189682198	-0,48511024	0,627598146	0,71143229
gir_mul_17151	21,65875368	0,140135576	0,293887827	0,47683355	0,633480653	0,715460503
gir_mul_5ht-r2	2,155943643	0,270455089	0,571516516	0,473223576	0,636053679	0,717048405
gir_mul_hcng	32,68949777	-0,10351696	0,242917079	-0,42614112	0,67000502	0,746556192
gir_mul_27566	25,84885509	0,098068603	0,25810246	0,379959971	0,703975129	0,774297027
gir_mul_17028	22,46048454	-0,11135196	0,330911911	-0,3365003	0,736493625	0,803162378
gir_mul_31312	3,960861055	-0,12970501	0,431429401	-0,30064017	0,763688898	0,823754316
gir_mul_ip3r	27,56113297	-0,06652088	0,269879036	-0,24648407	0,805307532	0,858100845
gir_mul_23489	29,78235157	0,048728132	0,215998582	0,225594683	0,821516676	0,8705413

gir_mul_07503	43,2356528	-0,0376377	0,180511281	-0,20850608	0,834833833	0,879797439
gir_mul_pis	17,65302883	0,046272596	0,239535508	0,193176355	0,846820856	0,888747993
gir_mul_maguk	311,6261645	0,034636395	0,192037711	0,180362464	0,856868021	0,896254191
gir_mul_plcb	29,04438214	-0,07031593	0,43179304	-0,16284637	0,870639395	0,906338265
gir_mul_best-b	2,265913688	-0,09722694	0,60717025	-0,16013126	0,872777679	0,907948875
gir_mul_03496	100,8685474	-0,02012933	0,148260112	-0,13577036	0,892002821	0,921086611
gir_mul_six-1-2	25,89266082	-0,04265757	0,354833638	-0,12021851	0,904310056	0,930948102
gir_mul_34343	22,87318432	0,031473019	0,29735115	0,105844617	0,915705636	0,938767716
gir_mul_21613	223,8170042	-0,02686943	0,257700686	-0,10426602	0,916958242	0,939591635
gir_mul_ddryk	46,49176797	-0,01725445	0,249283586	-0,06921614	0,944817577	0,962039208
gir_mul_myoVI	80,04228741	0,016394905	0,244402322	0,067081624	0,946516723	0,96313328
gir_mul_00978	104,9102211	-0,00112299	0,154010325	-0,00729168	0,994182134	0,995802907

Table S3c. Results of the differential gene expression analysis in planarian eye-related gene orthologs. DESeq2 Wald test pair-wise comparison between *D. japonica* and *G. multidiverticulata* morphotypes.

***D. japonica and G. multidiverticulata* non-discernible eyes:**

gene	baseMean	log2FoldChan	lfcSE	stat	pvalue	padj
gir_mul_tph	153,8029805	-5,03251907	0,348671258	-14,433421	3,19E-47	3,27E-45
gir_mul_27566	28,23484008	3,540890012	0,277635781	12,7537236	2,97E-37	1,72E-35
gir_mul_05359	131,6006835	-2,07157259	0,175200131	-11,8240356	2,93E-32	1,13E-30
gir_mul_gucy-2	41,04313447	-4,36155839	0,374714492	-11,6396843	2,59E-31	8,94E-30
gir_mul_pitp	187,0888635	-4,14205808	0,36046271	-11,4909475	1,46E-30	4,55E-29
gir_mul_03464	35,1907097	-3,06226841	0,274453674	-11,1576878	6,57E-29	1,84E-27
gir_mul_19866	60,57428559	3,320809856	0,297825608	11,15018241	7,15E-29	1,98E-27
gir_mul_32930	101,5093707	-2,19518286	0,206571152	-10,6267639	2,24E-26	5,13E-25
gir_mul_eya	62,11070008	-2,63555196	0,266237099	-9,89926634	4,19E-23	7,32E-22
gir_mul_cds	106,6824874	-1,40046762	0,163301574	-8,57595909	9,83E-18	1,05E-16
gir_mul_trpc-2	159,7072354	-2,42247824	0,295190324	-8,2064961	2,28E-16	2,19E-15
gir_mul_cdkl1	30,18440601	-2,34751041	0,287198644	-8,17382135	2,99E-16	2,82E-15
gir_mul_soxB	45,11260262	-2,34207715	0,287345703	-8,15072966	3,62E-16	3,36E-15
gir_mul_ddc	55,74058598	-2,86328954	0,354170438	-8,08449613	6,24E-16	5,59E-15
gir_mul_12199	255,3949138	-1,76893307	0,232202412	-7,61806503	2,58E-14	1,93E-13
gir_mul_5ht-r2	8,761452344	-3,62819016	0,487955472	-7,43549434	1,04E-13	7,23E-13
gir_mul_ncx	270,6417297	-1,05010164	0,144646505	-7,25977884	3,88E-13	2,54E-12
gir_mul_cng	12,40662764	-2,17982303	0,302112922	-7,21525918	5,38E-13	3,50E-12
gir_mul_best-b	8,188754373	-3,40690395	0,48080514	-7,08583098	1,38E-12	8,63E-12
gir_mul_cgs-pde	45,73559461	-1,78799006	0,252698165	-7,07559573	1,49E-12	9,25E-12
gir_mul_pcdhP-4	115,3713379	-1,70359822	0,240773042	-7,07553555	1,49E-12	9,25E-12
gir_mul_27330	91,74803745	-1,41765112	0,20079995	-7,06001733	1,66E-12	1,03E-11
gir_mul_tyrosinase	50,53397091	-2,89920792	0,415340054	-6,98032345	2,95E-12	1,79E-11
gir_mul_ip3k	92,14420043	-2,05059943	0,297612806	-6,89015859	5,57E-12	3,26E-11
gir_mul_06952	19,26149997	1,762882702	0,259299516	6,798634748	1,06E-11	5,92E-11
gir_mul_32587	26,62940623	-2,01189721	0,297552827	-6,76147904	1,37E-11	7,54E-11
gir_mul_rgs6-7-9	211,5721064	1,152841171	0,178163317	6,470698836	9,76E-11	4,87E-10
gir_mul_fzdP-1	160,8554709	-2,22073506	0,345602288	-6,42569549	1,31E-10	6,42E-10
gir_mul_22592	9,492110535	-3,2432337	0,506945944	-6,39759277	1,58E-10	7,64E-10
gir_mul_07503	38,0956337	1,05668351	0,16638846	6,350701898	2,14E-10	1,02E-09
gir_mul_07357	133,7911204	-1,48927096	0,234526748	-6,35011135	2,15E-10	1,02E-09
gir_mul_gpcrP-1	32,15634575	-1,95207474	0,310629363	-6,28425697	3,29E-10	1,52E-09
gir_mul_03129	62,22823339	-1,60584688	0,260620425	-6,16163094	7,20E-10	3,19E-09
gir_mul_17151	37,51809702	-1,40197248	0,234576135	-5,97662023	2,28E-09	9,55E-09
gir_mul_15984	136,8689669	-1,12166074	0,190738312	-5,88062632	4,09E-09	1,66E-08
gir_mul_29449	34,9954216	-2,58942107	0,442269153	-5,85485342	4,77E-09	1,92E-08
gir_mul_eag	9,21119092	-3,31892413	0,582953259	-5,69329371	1,25E-08	4,81E-08
gir_mul_cpo	7,68867504	-3,38697847	0,604012996	-5,6074596	2,05E-08	7,77E-08
gir_mul_01362	64,96532255	-1,84926229	0,33390355	-5,53831276	3,05E-08	1,12E-07
gir_mul_29424	265,3517904	-1,23695754	0,22473877	-5,50397929	3,71E-08	1,35E-07
gir_mul_arrestin	347,8164149	-2,56112677	0,46956091	-5,45430148	4,92E-08	1,77E-07

gir_mul_20047	199,1218002	-1,60402612	0,295463286	-5,42885087	5,67E-08	2,02E-07
gir_mul_34307	10,76524664	-1,88811301	0,348598646	-5,41629473	6,08E-08	2,15E-07
gir_mul_gna-o	148,3295723	-1,69429811	0,31508819	-5,37721871	7,56E-08	2,63E-07
gir_mul_09627	98,15064895	-1,46219167	0,273095247	-5,35414545	8,60E-08	2,98E-07
gir_mul_pis	31,30104604	-1,00711166	0,196989166	-5,11252308	3,18E-07	1,04E-06
gir_mul_dlx	29,67767601	-1,24676089	0,24486327	-5,09166152	3,55E-07	1,15E-06
gir_mul_13049	71,18934317	1,796568716	0,361359302	4,971696337	6,64E-07	2,07E-06
gir_mul_59sley	12,22706275	-1,70951447	0,344220616	-4,96633378	6,82E-07	2,13E-06
gir_mul_stim	99,28614226	-0,71575425	0,145814356	-4,90866789	9,17E-07	2,78E-06
gir_mul_dagk	89,20610762	-1,2596388	0,2584168	-4,87444625	1,09E-06	3,26E-06
gir_mul_pctaire	31,92360801	-1,15640945	0,237947843	-4,85992825	1,17E-06	3,48E-06
gir_mul_23802	4,990131495	-1,98988282	0,409529994	-4,8589428	1,18E-06	3,49E-06
gir_mul_30263	35,40590614	-1,40265376	0,294432872	-4,76391699	1,90E-06	5,49E-06
gir_mul_pde1c	50,62383167	-1,45611305	0,306562382	-4,74980994	2,04E-06	5,87E-06
gir_mul_04256	36,98884852	-0,98428782	0,208897001	-4,71183318	2,45E-06	6,99E-06
gir_mul_rops2	81,0802012	-1,710034	0,367441512	-4,65389442	3,26E-06	9,11E-06
gir_mul_26028	178,7276827	0,768827022	0,168530244	4,561952808	5,07E-06	1,38E-05
gir_mul_crf-r	7,98992237	1,913715651	0,422450132	4,53003918	5,90E-06	1,60E-05
gir_mul_ip3r	45,44305144	-1,28888978	0,287451157	-4,48385664	7,33E-06	1,95E-05
gir_mul_08086	142,5405381	-0,6955385	0,155329272	-4,47783272	7,54E-06	2,01E-05
gir_mul_nkx1	7,019976608	-1,37918989	0,316570524	-4,3566592	1,32E-05	3,42E-05
gir_mul_otxA	24,32650281	-1,23138578	0,285834637	-4,30803557	1,65E-05	4,21E-05
gir_mul_5ht-r1	44,9736021	-1,0386708	0,241146154	-4,30722522	1,65E-05	4,22E-05
gir_mul_23388	140,243473	-0,71933754	0,168251472	-4,27537143	1,91E-05	4,84E-05
gir_mul_23489	43,47255801	-0,8342798	0,195978544	-4,2569956	2,07E-05	5,24E-05
gir_mul_22239	8,776282303	-1,57925874	0,371088152	-4,25575092	2,08E-05	5,26E-05
gir_mul_foxq2	20,69925399	-1,4321633	0,348919886	-4,10456199	4,05E-05	9,74E-05
gir_mul_14918	67,53209867	-1,25340387	0,307322003	-4,07847099	4,53E-05	0,000108446
gir_mul_21613	271,8290499	-0,90787957	0,227897561	-3,98371782	6,78E-05	0,000159569
gir_mul_plcb	66,09165263	-2,03814472	0,511914264	-3,98141811	6,85E-05	0,000160731
gir_mul_20488	51,6241986	-0,91562126	0,232321835	-3,94117608	8,11E-05	0,000188682
gir_mul_04136	10,727532	2,268769993	0,576045126	3,938528236	8,20E-05	0,000190344
gir_mul_21691	73,35683266	1,503809153	0,382451997	3,932020664	8,42E-05	0,000195277
gir_mul_sk3	52,27470547	-0,65754965	0,168821931	-3,89493027	9,82E-05	0,00022601
gir_mul_pmca2	43,58350078	-1,09063283	0,284042412	-3,83968303	0,000123193	0,000279481
gir_mul_12518	13,50783314	-1,55135244	0,4098828	-3,78486834	0,00015379	0,000343963
gir_mul_meis	39,68757444	-0,9660363	0,25615587	-3,7712831	0,00016241	0,0003623
gir_mul_20798	27,87538524	0,931040883	0,251378465	3,703741614	0,000212443	0,00046613
gir_mul_myoVIIA	247,1958078	1,219533793	0,329783932	3,697978208	0,000217324	0,00047616
gir_mul_qdr	89,78470674	1,432073851	0,388275205	3,688295911	0,000225761	0,00049289
gir_mul_23244	71,65985833	-0,62994139	0,172099777	-3,66032661	0,000251894	0,000546066
gir_mul_maguk	188,3900653	1,071810515	0,293073755	3,65713578	0,000255049	0,000552517
gir_mul_07486	14,83594602	-1,42065767	0,39832324	-3,56659498	0,00036165	0,000765877
gir_mul_nhe-rf	34,22547868	0,955072874	0,268251552	3,5603629	0,000370343	0,000782428
gir_mul_gls	13,01514448	-1,35186858	0,393953389	-3,43154449	0,000600155	0,001230737
gir_mul_01915	87,8911569	-0,51125507	0,14959671	-3,41755558	0,000631862	0,001288026

gir_mul_17028	24,18822422	-1,09001114	0,327227169	-3,33105331	0,00086518	0,001731487
gir_mul_02471	28,90280463	-0,81248101	0,248149782	-3,27415564	0,001059782	0,002090995
gir_mul_04906	58,39761028	-0,86971689	0,269858239	-3,22286581	0,00126915	0,00246922
gir_mul_unc5-2	131,7490539	-0,69386469	0,215319525	-3,22248849	0,001270823	0,00247091
gir_mul_25626	245,7170577	-0,91225709	0,285376832	-3,19667537	0,001390213	0,002682683
gir_mul_hcng	48,73450429	-1,19896096	0,38211404	-3,13770455	0,001702764	0,003235041
gir_mul_09268	67,96501731	0,635664292	0,210896062	3,014111719	0,002577329	0,004755527
gir_mul_16656	23,04685092	-0,91584081	0,308996489	-2,96391981	0,003037474	0,005541363
gir_mul_09132	22,20592293	-0,90872946	0,306957611	-2,96043956	0,003072004	0,005591083
gir_mul_ninag	4,776976631	-1,52419932	0,519465053	-2,93417104	0,003344399	0,006029725
gir_mul_10513	87,06467042	0,388106534	0,140148191	2,769258241	0,005618408	0,009735619
gir_mul_nanos	1,852619331	1,78423075	0,658765505	2,70844593	0,006759913	0,011538019
gir_mul_ddryk	42,48694459	-0,69836274	0,258647506	-2,70005596	0,006932781	0,01181995
gir_mul_32281	373,5025628	0,897222766	0,336414851	2,667012953	0,007652872	0,012954256
gir_mul_robo-c	227,250325	-0,62302083	0,236794468	-2,6310616	0,008511861	0,014282456
gir_mul_03496	161,8743697	-0,51939693	0,20060751	-2,58912006	0,009622154	0,015988379
gir_mul_34512	27,07689255	-0,6938667	0,27759634	-2,49955276	0,012435018	0,020289409
gir_mul_klf	8,477726283	-1,1643169	0,50744474	-2,29447034	0,021763497	0,034046305
gir_mul_34343	32,82586646	-0,52826206	0,234390513	-2,25376896	0,024210707	0,037644771
gir_mul_13405	102,4808936	-0,77454847	0,352910404	-2,19474535	0,02818187	0,043207515
gir_mul_six-1-2	37,17700369	-0,91920313	0,424192686	-2,16694714	0,030238885	0,046062603
gir_mul_nol1	6,615666349	-0,73872736	0,344553317	-2,14401466	0,032031712	0,048433587
gir_mul_31312	2,849099128	-0,95524394	0,476328139	-2,00543253	0,044916827	0,066095896
gir_mul_arrb-2	12,13933403	-0,62130039	0,323228486	-1,92217089	0,054584258	0,078554312
gir_mul_09274	59,23856511	-0,53170917	0,285179659	-1,86447088	0,062255608	0,088150041
gir_mul_33093	146,21471	-0,44677743	0,241406747	-1,8507247	0,064209169	0,090499306
gir_mul_transferrin	6,367746159	-0,78566168	0,455707509	-1,72404813	0,084699154	0,116234464
gir_mul_unc5-1	101,2286112	0,585570262	0,34840865	1,68069955	0,092821288	0,126309269
gir_mul_ttbk	35,13464157	0,594498136	0,355708234	1,671308337	0,094660791	0,128584742
gir_mul_gpcrP-2	17,26936823	-0,55877344	0,347549869	-1,60775041	0,107889854	0,144385464
gir_mul_00978	148,9013267	-0,25998965	0,181899088	-1,42930707	0,152915994	0,198004957
gir_mul_nf-i-a	40,22991434	0,311221358	0,221206956	1,406923921	0,159449949	0,205599473
gir_mul_sp6-9	1,712131253	0,837951154	0,673419812	1,244322099	0,213381041	0,266206704
gir_mul_28813	15,36626653	-0,4970591	0,409922237	-1,21256924	0,225294555	0,279143676
gir_mul_cdh23	24,27639358	-0,39194316	0,344056526	-1,13918247	0,254627054	0,310604589
gir_mul_erm	12,70555631	-0,51107166	0,461317913	-1,10785132	0,267926032	0,325535424
gir_mul_26962	185,2625559	-0,22764108	0,23459958	-0,97033883	0,331877629	0,390728392
gir_mul_atp13	81,81563203	-0,22197406	0,249247391	-0,89057725	0,37315601	0,434500596
gir_mul_gna-q	118,8360515	0,430402727	0,486293353	0,885068084	0,376119912	0,437620215
gir_mul_smad6-7-2	64,83547997	0,305874062	0,403507535	0,758038042	0,448428209	0,510165919
gir_mul_best-a	7,149815895	0,333387722	0,568960709	0,585959131	0,55790298	0,616460733
gir_mul_inpp5	59,83665551	-0,11334885	0,204975951	-0,55298607	0,580272947	0,637740093
gir_mul_09720	14,94311608	-0,20889812	0,407882647	-0,5121525	0,608544281	0,665480298
gir_mul_abl	78,1429457	0,108222891	0,26416171	0,409684246	0,682037588	0,731791814
gir_mul_08711	36,99630071	-0,14547186	0,368101376	-0,3951951	0,692698911	0,741676154
gir_mul_fzdP-2	276,0584718	-0,09446611	0,239424463	-0,39455496	0,693171356	0,741676154

gir_mul_sans	7,87361062	0,166863729	0,462403321	0,360861874	0,718202706	0,763929107
gir_mul_10538	13,42763718	0,121330991	0,422721552	0,287023433	0,774094378	0,813526878
gir_mul_11992	18,29816225	0,070053191	0,312920986	0,223868624	0,82285953	0,855417719
gir_mul_egfr-1	37,87951788	0,052573399	0,276301689	0,190275344	0,849093373	0,878825936
gir_mul_08883	55,95030536	-0,02115287	0,186366988	-0,11350114	0,909633261	0,928665774
gir_mul_myoVI	121,9402657	-0,01422925	0,263481803	-0,0540047	0,956931423	0,966045055
gir_mul_ovo	9,396048794	0,014828965	0,49011659	0,030255996	0,975862891	0,980007359

D. japonica and G. multidiverticulata **discernible eyes:**

gene	baseMean	log2FoldChan	lfcSE	stat	pvalue	padj
gir_mul_gucy-2	47,29734913	-3,47511534	0,29155388	-11,9192903	9,39E-33	2,70E-31
gir_mul_tph	173,6985379	-4,23998664	0,362817463	-11,6862805	1,50E-31	3,91E-30
gir_mul_05359	143,6338907	-2,24269132	0,195551328	-11,4685558	1,90E-30	4,56E-29
gir_mul_pitp	215,5289694	-3,30438302	0,296019283	-11,1627289	6,21E-29	1,38E-27
gir_mul_27566	20,07838391	2,820627156	0,27347683	10,31395294	6,09E-25	1,02E-23
gir_mul_19866	54,3594172	2,979965057	0,295059065	10,09955435	5,55E-24	8,71E-23
gir_mul_gna-q	114,9988242	1,74272705	0,183233616	9,510957023	1,89E-21	2,54E-20
gir_mul_eya	72,94276954	-2,03430787	0,218914863	-9,29268958	1,50E-20	1,90E-19
gir_mul_03464	41,30467833	-2,32175088	0,250277893	-9,27669181	1,75E-20	2,20E-19
gir_mul_cds	119,2054641	-1,34334722	0,147642247	-9,09866418	9,14E-20	1,07E-18
gir_mul_rgs6-7-9	274,8132953	1,480824012	0,185838191	7,9683514	1,61E-15	1,22E-14
gir_mul_07486	13,79210009	-2,60141896	0,334790035	-7,77029985	7,83E-15	5,56E-14
gir_mul_trpc-2	181,7155862	-2,16944651	0,28172317	-7,70063218	1,35E-14	9,36E-14
gir_mul_26028	223,6031558	1,039175036	0,140841618	7,378323602	1,60E-13	1,01E-12
gir_mul_ncx	315,5092194	-0,81772525	0,111388001	-7,34123284	2,12E-13	1,31E-12
gir_mul_06952	18,63253488	1,536672175	0,212360723	7,236141181	4,62E-13	2,77E-12
gir_mul_32930	127,2938636	-1,33498959	0,191183902	-6,98275102	2,89E-12	1,63E-11
gir_mul_soxB	57,45442704	-1,33837138	0,192305073	-6,95962598	3,41E-12	1,90E-11
gir_mul_22592	10,11041135	-3,2302425	0,47718163	-6,76941923	1,29E-11	6,75E-11
gir_mul_5ht-r2	10,56551029	-2,40156581	0,356636418	-6,73393319	1,65E-11	8,51E-11
gir_mul_07503	42,61885469	1,056125677	0,157547311	6,703546195	2,03E-11	1,04E-10
gir_mul_ip3k	108,3673752	-1,66540634	0,252943168	-6,58411277	4,58E-11	2,27E-10
gir_mul_gls	12,52598137	-2,05744171	0,315291859	-6,52551486	6,78E-11	3,32E-10
gir_mul_23802	5,278322007	-2,83881426	0,446414169	-6,3591491	2,03E-10	9,46E-10
gir_mul_best-b	9,98713566	-2,3397426	0,379880184	-6,1591594	7,31E-10	3,20E-09
gir_mul_ddc	67,21041508	-2,05840485	0,336009524	-6,12603127	9,01E-10	3,87E-09
gir_mul_cng	13,94393462	-2,02506721	0,333989761	-6,06326135	1,33E-09	5,63E-09
gir_mul_21691	100,3583243	1,925478872	0,317603956	6,062515387	1,34E-09	5,65E-09
gir_mul_04256	40,12653755	-1,13335673	0,190588657	-5,94661166	2,74E-09	1,11E-08
gir_mul_09268	91,5840411	1,085281342	0,183268599	5,921807371	3,18E-09	1,29E-08
gir_mul_04136	17,45636446	3,030659064	0,520812691	5,819096035	5,92E-09	2,32E-08
gir_mul_30263	38,7318847	-1,44725004	0,249010739	-5,81199848	6,17E-09	2,41E-08
gir_mul_pis	35,57046082	-0,92669247	0,168302392	-5,50611585	3,67E-08	1,30E-07
gir_mul_maguk	277,7518137	1,644300131	0,304833863	5,394086185	6,89E-08	2,37E-07
gir_mul_08711	26,78609121	-1,98076559	0,370931135	-5,33998202	9,30E-08	3,15E-07
gir_mul_03129	72,70656724	-1,27839836	0,239941624	-5,32795579	9,93E-08	3,32E-07

gir_mul_32587	32,47288449	-1,29648424	0,243633414	-5,32145496	1,03E-07	3,43E-07
gir_mul_eag	10,92308138	-2,27365684	0,432071128	-5,26222812	1,42E-07	4,65E-07
gir_mul_cpo	8,655056576	-2,74425754	0,523398115	-5,24315519	1,58E-07	5,11E-07
gir_mul_32281	629,6405066	1,773070929	0,343489485	5,16193656	2,44E-07	7,75E-07
gir_mul_dlx	33,65023782	-1,11605843	0,216276706	-5,16032658	2,47E-07	7,81E-07
gir_mul_dagk	101,4510148	-1,14834615	0,22718996	-5,0545638	4,31E-07	1,33E-06
gir_mul_cdkl1	37,19277284	-1,50199737	0,300672226	-4,99546428	5,87E-07	1,77E-06
gir_mul_01362	74,71814913	-1,57406929	0,315658892	-4,98661478	6,14E-07	1,85E-06
gir_mul_28813	13,63183114	-1,51364162	0,306785323	-4,93387885	8,06E-07	2,38E-06
gir_mul_12199	330,7656124	-0,91844522	0,188222888	-4,87956185	1,06E-06	3,10E-06
gir_mul_nf-i-a	57,82296929	0,934436301	0,19233942	4,858267242	1,18E-06	3,43E-06
gir_mul_fzdP-1	193,1891968	-1,60544612	0,330856573	-4,85239301	1,22E-06	3,53E-06
gir_mul_07357	156,5673652	-1,15230914	0,237654534	-4,84867309	1,24E-06	3,59E-06
gir_mul_02471	30,62364062	-1,1038474	0,231798786	-4,76209311	1,92E-06	5,42E-06
gir_mul_arrestin	409,8793608	-2,08531353	0,452402196	-4,60942397	4,04E-06	1,10E-05
gir_mul_foxq2	23,94419931	-1,25275894	0,278198854	-4,50310603	6,70E-06	1,78E-05
gir_mul_27330	112,982079	-0,90350408	0,20139335	-4,48626572	7,25E-06	1,92E-05
gir_mul_rops2	89,93070978	-1,72603081	0,390361457	-4,421622	9,80E-06	2,54E-05
gir_mul_34307	12,97390527	-1,3324615	0,314850019	-4,23205151	2,32E-05	5,71E-05
gir_mul_11992	30,49158285	1,148832543	0,271787938	4,226944544	2,37E-05	5,83E-05
gir_mul_03496	174,0522315	-0,63293578	0,151003628	-4,19152696	2,77E-05	6,71E-05
gir_mul_23388	158,9608597	-0,62186851	0,148428642	-4,18967997	2,79E-05	6,76E-05
gir_mul_crf-r	7,527397152	1,661912936	0,398505898	4,170359696	3,04E-05	7,31E-05
gir_mul_gna-o	192,1664727	-0,85621756	0,210192927	-4,07348415	4,63E-05	0,000109372
gir_mul_ip3r	53,9650476	-0,99968275	0,246626588	-4,05342651	5,05E-05	0,000118553
gir_mul_10513	99,27040542	0,474396426	0,1176106	4,033619638	5,49E-05	0,000128518
gir_mul_29449	43,63309682	-1,64409573	0,41801245	-3,93312623	8,38E-05	0,000190547
gir_mul_09627	117,3167463	-1,08750224	0,281976485	-3,85671253	0,000114922	0,000255503
gir_mul_17151	44,97215907	-0,93498275	0,251509199	-3,71748928	0,000201213	0,000433869
gir_mul_plcb	78,35721672	-1,58432589	0,427396156	-3,70692592	0,00020979	0,0004511
gir_mul_unc5-2	143,1634822	-0,79836384	0,219480672	-3,6375132	0,000275283	0,000580169
gir_mul_pcdhP-4	145,8178096	-0,96250732	0,265598947	-3,62391239	0,00029018	0,000609893
gir_mul_otxA	28,72993003	-0,88879877	0,251013465	-3,54084101	0,000398854	0,000826447
gir_mul_13049	59,5422596	1,221399533	0,346035883	3,529690393	0,000416046	0,000859191
gir_mul_myoVIIA	252,5117506	1,036396634	0,298545595	3,47148526	0,000517588	0,001052622
gir_mul_23244	82,56102097	-0,51019819	0,148719981	-3,43059611	0,000602257	0,001215973
gir_mul_stim	118,3179309	-0,41283591	0,124204113	-3,32385057	0,000887838	0,001744493
gir_mul_gpcrP-1	41,55637889	-1,02716044	0,316107295	-3,24940441	0,001156469	0,002218489
gir_mul_cgs-pde	61,95237678	-0,71135514	0,220131411	-3,23150222	0,001231414	0,002351992
gir_mul_ttbk	45,9251801	1,003021607	0,319524519	3,139106852	0,001694636	0,003164075
gir_mul_pctaire	40,12931928	-0,54240832	0,173228634	-3,13117007	0,001741113	0,003244945
gir_mul_22239	11,19721954	-0,88467081	0,282682798	-3,12955304	0,001750725	0,003260884
gir_mul_klf	9,329079876	-1,28569664	0,415561183	-3,0938805	0,00197557	0,003645402
gir_mul_15984	171,4638579	-0,59606607	0,194009891	-3,07234885	0,002123814	0,003906141
gir_mul_23489	52,29887175	-0,56913947	0,18526746	-3,07198833	0,00212638	0,003908526
gir_mul_01915	100,1329264	-0,41635364	0,136944947	-3,04029943	0,002363431	0,004295497

gir_mul_nkx1	8,50652825	-0,81661911	0,282052265	-2,89527585	0,003788255	0,006664643
gir_mul_robo-c	261,538134	-0,47979742	0,166600141	-2,87993407	0,003977583	0,006957944
gir_mul_20047	261,5074733	-0,7404494	0,262646049	-2,81919107	0,004814485	0,008313227
gir_mul_08086	170,6523874	-0,40238304	0,144868714	-2,7775703	0,005476699	0,009377743
gir_mul_14918	81,18049707	-0,84672701	0,306751196	-2,76030549	0,005774733	0,00983879
gir_mul_29424	334,77734	-0,66170701	0,241982424	-2,73452509	0,006247033	0,010596539
gir_mul_00978	161,2078087	-0,32435744	0,121774002	-2,66360168	0,007730904	0,012907212
gir_mul_pde1c	64,37232016	-0,72052505	0,272479462	-2,64432794	0,008185331	0,01362156
gir_mul_cdh23	23,93840646	-0,86007708	0,35080824	-2,45170148	0,014218256	0,022821896
gir_mul_20488	62,36349577	-0,53170868	0,219903254	-2,41792092	0,015609469	0,024809058
gir_mul_smad6-7-2	86,94089398	0,771517261	0,321177233	2,402154267	0,016298833	0,025811379
gir_mul_nol1	7,10705683	-0,87218783	0,371264483	-2,34923584	0,018811987	0,029531503
gir_mul_nhe-rf	31,68505915	0,55166728	0,236119023	2,336394893	0,01947067	0,030473678
gir_mul_34343	36,77895705	-0,46071308	0,200138877	-2,30196693	0,021337037	0,033242564
gir_mul_hcng	59,31238791	-0,75560941	0,335166171	-2,25443221	0,024168991	0,03729588
gir_mul_abl	100,4273238	0,516788027	0,229542254	2,251385174	0,024361152	0,037548775
gir_mul_09274	66,81865554	-0,43083671	0,191794957	-2,24634013	0,02468223	0,037992607
gir_mul_atp13	88,28360111	-0,33970278	0,165196906	-2,05635072	0,039748723	0,058716669
gir_mul_20798	24,56491905	0,398543983	0,198917578	2,003563421	0,045116848	0,065762454
gir_mul_meis	50,41345108	-0,39749752	0,210821492	-1,88546964	0,059366459	0,08484468
gir_mul_six-1-2	44,23437095	-0,66389844	0,380805626	-1,74340501	0,081262893	0,112027743
gir_mul_ninag	5,980235334	-0,86103132	0,560096171	-1,53729192	0,124221839	0,164329577
gir_mul_08883	57,69060176	-0,25360743	0,165815377	-1,52945662	0,126151284	0,166595494
gir_mul_erm	13,94444961	-0,59090857	0,408061476	-1,44808713	0,147592683	0,191621387
gir_mul_pmca2	56,72063055	-0,4469925	0,309066116	-1,44626821	0,148101989	0,192201527
gir_mul_unc5-1	110,5869832	0,517511614	0,364720725	1,41892571	0,155920676	0,201329383
gir_mul_21613	348,1944033	-0,37150409	0,266960768	-1,39160556	0,164041874	0,211018748
gir_mul_26962	246,7531101	0,32405787	0,236726263	1,368913893	0,171026193	0,219178507
gir_mul_59sley	17,33654222	-0,37830496	0,294600816	-1,28412733	0,199097408	0,251177829
gir_mul_31312	4,747610142	0,444026538	0,355454352	1,249180196	0,211599174	0,265210044
gir_mul_04906	74,48228561	-0,30879657	0,248160139	-1,24434397	0,213372995	0,266998253
gir_mul_inpp5	74,11058048	0,218110481	0,180186027	1,210473895	0,226097102	0,28173164
gir_mul_myoVI	124,1063916	-0,31726595	0,264643404	-1,19884323	0,230588908	0,286479233
gir_mul_sk3	66,45092404	-0,18509387	0,15830554	-1,16921915	0,242315348	0,29931928
gir_mul_tyrosinase	81,42932333	-0,52405425	0,452212678	-1,15886678	0,246510491	0,303405112
gir_mul_12518	18,64506876	-0,4646636	0,402104497	-1,15557922	0,247853311	0,304814004
gir_mul_arrb-2	14,06093963	-0,31721891	0,29622315	-1,0708782	0,284224199	0,342956816
gir_mul_nanos	1,413659868	0,738983465	0,697688642	1,059188039	0,289514151	0,347846561
gir_mul_09720	20,8589135	0,442764058	0,421671322	1,050021745	0,293708115	0,352433571
gir_mul_sp6-9	1,904180298	0,665553847	0,634102348	1,049600036	0,293902041	0,352433571
gir_mul_transferrin	7,708050824	-0,49154523	0,480227789	-1,02356683	0,30603992	0,364852706
gir_mul_ovo	12,4118609	0,460262208	0,472848371	0,973382245	0,33036336	0,389176133
gir_mul_17028	32,85256184	-0,24404153	0,338691618	-0,720542	0,471191352	0,529918052
gir_mul_16656	34,60349914	0,177194566	0,24684631	0,717833563	0,472859916	0,531406122
gir_mul_best-a	6,579655722	-0,34905739	0,53848918	-0,64821616	0,516845152	0,57474974
gir_mul_33093	192,8966793	0,127391413	0,19667144	0,647737227	0,517154922	0,574886451

gir_mul_5ht-r1	63,58226533	-0,13458633	0,209946862	-0,64104948	0,521490535	0,578660791
gir_mul_ddryk	60,75644	0,146802669	0,239979872	0,61172909	0,540717012	0,596204461
gir_mul_qdr	57,88020723	0,12973308	0,225757031	0,574657982	0,56552263	0,619698409
gir_mul_gpcrP-2	20,41821615	-0,15823432	0,28912074	-0,54729493	0,584176124	0,638547046
gir_mul_10538	15,43580697	0,220280651	0,404650534	0,544372569	0,586185118	0,640288111
gir_mul_fzdP-2	305,7269366	-0,10513853	0,21509064	-0,48881033	0,624975986	0,67570313
gir_mul_sans	8,796515637	0,175937176	0,403266412	0,436280262	0,662633401	0,710921539
gir_mul_09132	30,28785372	-0,11960868	0,336194662	-0,3557721	0,722011243	0,764760274
gir_mul_34512	34,61451993	-0,07684766	0,25876859	-0,29697447	0,766486001	0,803296126
gir_mul_13405	146,0215749	0,077483619	0,334846743	0,231400246	0,817003874	0,84720609
gir_mul_25626	356,8799567	0,058269068	0,267928829	0,217479651	0,827834564	0,855929755
gir_mul_egfr-1	42,92531004	0,050614483	0,313488895	0,161455425	0,871734717	0,892027843

Table S3d. Results of the differential gene expression analysis in planarian eye-related gene orthologs. DESeq2 Wald test pair-wise comparison between *S. mediterranea* and *G. multidiverticulata* morphotypes.

***S. mediterranea and G. multidiverticulata* non-discernible eyes:**

gene	baseMean	log2FoldChan	lfcSE	stat	pvalue	padj
gir_mul_tph	160,5051455	5,168691807	0,294275196	17,5641436	4,64E-69	1,10E-66
gir_mul_gucy-2	34,62219212	4,212564535	0,33483757	12,58091957	2,69E-36	9,29E-35
gir_mul_27566	31,2076629	-3,40955438	0,274977904	-12,3993758	2,63E-35	8,26E-34
gir_mul_05359	162,4782955	2,46992568	0,204900858	12,05424763	1,84E-33	5,10E-32
gir_mul_32930	148,5210231	2,890321249	0,240246891	12,03062914	2,45E-33	6,73E-32
gir_mul_cdkl1	47,0438357	3,178460993	0,265099415	11,98969446	4,02E-33	1,09E-31
gir_mul_03464	40,36163341	3,354520829	0,282150866	11,88910345	1,35E-32	3,45E-31
gir_mul_cng	24,54991028	3,365038001	0,295840947	11,37448362	5,60E-30	1,19E-28
gir_mul_arrestin	1089,769735	4,413194945	0,391922952	11,26036361	2,06E-29	3,98E-28
gir_mul_32281	307,0345569	-2,66821337	0,240572055	-11,0911193	1,39E-28	2,54E-27
gir_mul_21613	501,7610087	2,110780558	0,191995827	10,9938877	4,09E-28	7,10E-27
gir_mul_cgs-pde	61,29908946	2,399471674	0,22028629	10,89251482	1,25E-27	2,13E-26
gir_mul_inpp5	34,84962821	-2,79615797	0,264196396	-10,5836341	3,55E-26	5,37E-25
gir_mul_cds	34,70080418	-3,01509602	0,287062134	-10,5032871	8,34E-26	1,24E-24
gir_mul_pitp	154,643176	3,930825301	0,375420596	10,47045725	1,18E-25	1,71E-24
gir_mul_ninag	23,25559959	4,274513166	0,410420323	10,41496468	2,12E-25	3,00E-24
gir_mul_07503	34,26826942	-2,10138442	0,202284709	-10,3882514	2,80E-25	3,92E-24
gir_mul_ip3k	133,9943897	2,723552591	0,264105265	10,31237524	6,20E-25	8,39E-24
gir_mul_32587	35,46114122	2,652911616	0,264042615	10,0472858	9,44E-24	1,18E-22
gir_mul_01915	137,2861893	1,46111606	0,146053187	10,00399986	1,46E-23	1,80E-22
gir_mul_29449	37,88623784	2,892883833	0,290231075	9,96751928	2,11E-23	2,59E-22
gir_mul_59sley	23,05010016	3,020343837	0,303326846	9,957390437	2,34E-23	2,86E-22
gir_mul_tyrosinase	45,4866119	2,813813493	0,28352878	9,924260575	3,27E-23	3,94E-22
gir_mul_trpc-2	149,9486044	2,357835017	0,243330366	9,689851095	3,33E-22	3,71E-21
gir_mul_09627	112,8298692	1,773435495	0,191446238	9,263360372	1,98E-20	1,95E-19
gir_mul_03129	79,00076723	2,097165787	0,22691521	9,242067927	2,42E-20	2,34E-19
gir_mul_cpo	15,62878459	4,683264668	0,507893296	9,220961778	2,94E-20	2,84E-19
gir_mul_09132	34,80097313	1,929458075	0,210890208	9,149111742	5,74E-20	5,36E-19
gir_mul_rops2	95,6723593	1,988088554	0,225715615	8,807935375	1,27E-18	1,07E-17
gir_mul_22239	23,09035405	3,292010719	0,381777496	8,622851664	6,53E-18	5,17E-17
gir_mul_eya	39,92807652	1,91706319	0,227943231	8,410265918	4,09E-17	3,08E-16
gir_mul_5ht-r1	90,17535878	2,347345611	0,285926181	8,209621122	2,22E-16	1,55E-15
gir_mul_22592	9,897723271	3,571501619	0,447342713	7,98381536	1,42E-15	9,22E-15
gir_mul_12518	29,85790588	2,954971071	0,372763544	7,92719974	2,24E-15	1,42E-14
gir_mul_09720	49,34489233	2,647231664	0,343419082	7,708458271	1,27E-14	7,56E-14
gir_mul_plcb	135,7058773	3,254529527	0,426261415	7,635055428	2,26E-14	1,32E-13
gir_mul_best-b	8,776559563	3,61042658	0,485029074	7,443732287	9,79E-14	5,46E-13
gir_mul_5ht-r2	7,783570665	3,601174585	0,49409693	7,288397006	3,14E-13	1,67E-12
gir_mul_02471	60,63689358	2,288660401	0,314934884	7,267090816	3,67E-13	1,94E-12
gir_mul_28813	39,31578523	2,401706992	0,360993575	6,65304636	2,87E-11	1,28E-10
gir_mul_34512	37,46352164	1,504551667	0,232125041	6,481643098	9,07E-11	3,82E-10

gir_mul_pctaire	40,33902431	1,598685681	0,254287778	6,286915136	3,24E-10	1,29E-09
gir_mul_nhe-rf	34,29791994	-1,22471194	0,204758168	-5,98126052	2,21E-09	8,08E-09
gir_mul_gpcrP-2	29,2011705	1,76271386	0,3046742	5,785569835	7,23E-09	2,49E-08
gir_mul_soxB	33,58091706	1,806746081	0,312973566	5,772839235	7,79E-09	2,68E-08
gir_mul_19866	80,45362327	-1,49091207	0,261534242	-5,70063812	1,19E-08	4,04E-08
gir_mul_20798	27,18244463	-1,34295416	0,238531521	-5,63009097	1,80E-08	5,97E-08
gir_mul_eag	6,181397234	2,834440356	0,504701992	5,616067308	1,95E-08	6,47E-08
gir_mul_16656	31,21746896	1,54044277	0,275639742	5,58860909	2,29E-08	7,54E-08
gir_mul_04136	32,25622784	1,464627531	0,264567716	5,535926873	3,10E-08	1,00E-07
gir_mul_nkx1	9,139119875	1,911541092	0,347864402	5,495075321	3,91E-08	1,24E-07
gir_mul_26028	168,1196454	-1,35260459	0,250528418	-5,39900666	6,70E-08	2,09E-07
gir_mul_06952	21,94838552	-1,38911434	0,257913883	-5,38596186	7,21E-08	2,24E-07
gir_mul_10538	27,93333203	1,624446952	0,304331971	5,337746625	9,41E-08	2,88E-07
gir_mul_pcdhP-4	110,1017327	1,613957896	0,302553528	5,33445406	9,58E-08	2,93E-07
gir_mul_20488	62,41455928	1,291360988	0,2461692	5,245826804	1,56E-07	4,67E-07
gir_mul_six-1-2	57,82340508	1,79922326	0,347895803	5,171730278	2,32E-07	6,80E-07
gir_mul_pde1c	49,05310073	1,424192973	0,275540562	5,16872349	2,36E-07	6,90E-07
gir_mul_pmca2	46,75372919	1,27420213	0,250631662	5,083963133	3,70E-07	1,06E-06
gir_mul_egfr-1	68,14412826	1,311730418	0,263582944	4,976537555	6,47E-07	1,80E-06
gir_mul_nanos	5,758371681	1,953101169	0,408727983	4,778486553	1,77E-06	4,66E-06
gir_mul_dagk	84,61761052	1,133114993	0,237939285	4,762202222	1,91E-06	5,00E-06
gir_mul_04256	49,03530393	1,54915127	0,331541582	4,672570056	2,97E-06	7,62E-06
gir_mul_arrb-2	18,03134083	1,450756061	0,319290879	4,54368151	5,53E-06	1,36E-05
gir_mul_ncx	252,779661	0,871148363	0,199833852	4,359363319	1,30E-05	3,08E-05
gir_mul_ddc	25,594359	1,516921103	0,353112466	4,295858254	1,74E-05	4,05E-05
gir_mul_23388	144,5315822	0,767457292	0,184513578	4,15935402	3,19E-05	7,18E-05
gir_mul_13405	131,0006011	1,284903965	0,310692178	4,135617365	3,54E-05	7,90E-05
gir_mul_crf-r	8,835699113	-1,63239695	0,397029183	-4,11152889	3,93E-05	8,75E-05
gir_mul_dlx	26,5740909	1,055661313	0,264655622	3,988811221	6,64E-05	0,000143148
gir_mul_best-a	15,01480941	1,507451796	0,37938687	3,973389472	7,09E-05	0,000151999
gir_mul_foxq2	20,44355554	1,447495079	0,371860198	3,892578675	9,92E-05	0,000208974
gir_mul_23489	42,17217341	0,69603012	0,179252618	3,882956516	0,000103194	0,000216947
gir_mul_25626	255,0223998	0,964615853	0,254425983	3,791341758	0,000149836	0,000307268
gir_mul_otxA	20,5972394	0,916471365	0,244799215	3,743767581	0,000181281	0,000367101
gir_mul_07357	89,67611899	0,624625215	0,167184516	3,736142728	0,000186865	0,000378158
gir_mul_29424	211,3971519	0,716925033	0,192379526	3,726618149	0,000194066	0,000392215
gir_mul_sp6-9	4,672186398	1,677727251	0,451234294	3,71808454	0,000200739	0,000404107
gir_mul_cdh23	36,1148872	1,283132251	0,357533879	3,588841017	0,000332151	0,000654928
gir_mul_ddryk	49,50019191	1,036883492	0,293490148	3,532941388	0,000410964	0,000798547
gir_mul_34343	46,0129499	1,298303127	0,372142119	3,488729338	0,000485322	0,000934762
gir_mul_31312	4,117957186	1,753550943	0,510848147	3,432626612	0,000597765	0,001139906
gir_mul_smad6-7-2	121,1916408	1,226348723	0,359473729	3,411511397	0,000646038	0,001225877
gir_mul_hcng	44,6512591	1,028827585	0,319428405	3,220839376	0,001278158	0,002323511
gir_mul_pis	26,65545988	0,63849477	0,204014015	3,129661307	0,00175008	0,003138707
gir_mul_stim	67,54606378	-0,43887867	0,147055034	-2,98445187	0,002840869	0,004950584
gir_mul_11992	24,95121575	0,761271341	0,263943536	2,884220436	0,00392384	0,006675088

gir_mul_myoVI	165,5970819	0,719397142	0,266037495	2,704119362	0,006848568	0,011186238
gir_mul_21691	90,42802905	-0,7868684	0,293475555	-2,68120595	0,007335735	0,011912335
gir_mul_26962	152,1580085	-0,50558434	0,197492323	-2,56002024	0,010466607	0,016513568
gir_mul_04906	54,89055869	0,719703085	0,281209384	2,559313897	0,010487899	0,016533232
gir_mul_34307	6,211437003	0,860899497	0,336526727	2,558190563	0,010521842	0,016578237
gir_mul_08086	129,8473167	0,427604904	0,171950958	2,486784079	0,012890361	0,020077047
gir_mul_qdr	105,1138436	-0,8758954	0,353982149	-2,47440558	0,013345809	0,020751147
gir_mul_unc5-2	124,3982799	0,523791735	0,218372027	2,398621029	0,016456937	0,025243824
gir_mul_17151	26,52346326	0,598977596	0,262713446	2,279965512	0,022609734	0,033757048
gir_mul_33093	106,6545212	-0,63222098	0,278575463	-2,26947835	0,023239252	0,034628635
gir_mul_ip3r	35,75330137	0,720531522	0,33159718	2,172912094	0,029786934	0,043581645
gir_mul_myoVIIA	304,0309401	-0,66328572	0,314799121	-2,10701263	0,035116485	0,050870912
gir_mul_10513	91,40087182	-0,36012779	0,171043716	-2,10547218	0,035250227	0,051040601
gir_mul_12199	141,3034913	0,483962328	0,237251203	2,039873021	0,041362974	0,059304936
gir_mul_atp13	101,7029333	0,676346441	0,355629113	1,901830917	0,057193263	0,0793018
gir_mul_27330	57,56703642	0,349159195	0,184998549	1,887361807	0,059111678	0,081887922
gir_mul_08883	51,90637981	-0,37492019	0,207322149	-1,80839427	0,070545159	0,096252029
gir_mul_ovo	11,39712534	0,501913905	0,326702162	1,536304207	0,124463788	0,163413756
gir_mul_klf	6,833680027	0,743752055	0,486065251	1,530148584	0,125979955	0,165143872
gir_mul_09268	77,9508774	-0,31140346	0,208036489	-1,49686944	0,134427233	0,17524568
gir_mul_nol1	4,320178123	-0,63499302	0,428428454	-1,48214484	0,138301759	0,179991366
gir_mul_gna-o	83,74926584	0,442962804	0,306523177	1,445120099	0,148424154	0,19186537
gir_mul_maguk	296,9814284	0,290728176	0,205256942	1,416410931	0,156655225	0,201404501
gir_mul_17028	18,0032529	0,398098784	0,334132794	1,191438826	0,233481358	0,288241645
gir_mul_rgs6-7-9	287,3217337	-0,22364712	0,188612679	-1,18574807	0,235721806	0,290774009
gir_mul_fzdP-1	64,14077763	0,310124046	0,267553558	1,159110153	0,246411288	0,302382554
gir_mul_nf-i-a	50,7070918	0,226200653	0,204528542	1,105961303	0,268743269	0,327304599
gir_mul_erm	9,363064918	-0,43279991	0,393358912	-1,10026721	0,271215714	0,329793159
gir_mul_ttbk	48,68441411	0,318146597	0,29065305	1,094592322	0,273695281	0,332545175
gir_mul_transferrin	5,861977056	0,426165888	0,437672106	0,973710415	0,330200344	0,390849387
gir_mul_robo-c	173,4001642	-0,23764822	0,252059755	-0,94282494	0,345770453	0,407709388
gir_mul_sk3	44,30255552	0,202139107	0,222976354	0,906549523	0,364645065	0,427342329
gir_mul_07486	7,340078855	-0,3810517	0,43295163	-0,88012534	0,378791411	0,441899354
gir_mul_23244	54,83140576	-0,14851074	0,185040643	-0,80258445	0,422214958	0,485736819
gir_mul_gpcrP-1	14,42566859	0,223547274	0,279097326	0,800965301	0,423151734	0,486632409
gir_mul_00978	134,586185	-0,16034328	0,206606211	-0,7760816	0,437700806	0,501487991
gir_mul_gna-q	129,2448215	-0,30070854	0,394658839	-0,76194555	0,446092504	0,5099622
gir_mul_01362	26,91273606	-0,19416852	0,263928688	-0,73568555	0,461922088	0,526687128
gir_mul_meis	26,01743223	-0,16537439	0,225585687	-0,73308904	0,463504124	0,528295023
gir_mul_13049	123,5418277	0,183030092	0,264570758	0,691800159	0,489062837	0,553119632
gir_mul_gls	7,96530562	0,295854159	0,440404362	0,671778449	0,501724751	0,565070429
gir_mul_unc5-1	118,0401257	-0,22199925	0,331973353	-0,66872611	0,50367021	0,566306593
gir_mul_15984	91,4736461	0,12069587	0,188142235	0,641513959	0,521188815	0,584294098
gir_mul_30263	18,31524906	-0,19070344	0,300480398	-0,63466183	0,525648992	0,588422982
gir_mul_09274	52,04442327	0,195185995	0,322427911	0,60536321	0,54493769	0,606714401
gir_mul_03496	142,7988525	0,12130251	0,269468506	0,450154684	0,652598909	0,710120016

gir_mul_20047	105,0681294	0,120005425	0,278469085	0,430947029	0,666506887	0,721169367
gir_mul_08711	38,70318543	0,155725829	0,377199758	0,412847107	0,679718636	0,734430611
gir_mul_23802	2,232417663	0,202179379	0,519359719	0,38928583	0,697064717	0,750272038
gir_mul_abl	82,07429233	-0,10402884	0,274203461	-0,37938559	0,70440155	0,755318447
gir_mul_fzdP-2	270,2229196	-0,09312729	0,245764901	-0,37892837	0,704741058	0,755360535
gir_mul_14918	41,73205941	0,061158236	0,26871408	0,227595949	0,81996037	0,85674989
gir_mul_sans	8,716324934	0,043964259	0,394913153	0,111326397	0,911357523	0,931044542

***S. mediterranea and G. multidiverticulata* discernible eyes:**

gene	baseMean	log2FoldChan	lfcSE	stat	pvalue	padj
gir_mul_32281	598,4310581	-3,58711283	0,249123777	-14,398918	5,26E-47	2,21E-45
gir_mul_inpp5	48,59188909	-3,16671892	0,235196309	-13,4641523	2,54E-41	7,97E-40
gir_mul_tph	180,5364379	4,315735263	0,321478828	13,42463296	4,34E-41	1,33E-39
gir_mul_28813	39,71641957	3,341830583	0,252196931	13,2508773	4,46E-40	1,31E-38
gir_mul_gucy-2	40,1194552	3,27901864	0,25329292	12,94555981	2,49E-38	6,43E-37
gir_mul_05359	176,6437319	2,572161712	0,225207194	11,42131235	3,27E-30	5,32E-29
gir_mul_ip3k	154,6516458	2,305646919	0,204619127	11,26799313	1,89E-29	2,87E-28
gir_mul_cds	40,51221409	-3,11768528	0,277826796	-11,2216868	3,19E-29	4,74E-28
gir_mul_07503	39,36826819	-2,15779712	0,19402319	-11,1213361	9,88E-29	1,43E-27
gir_mul_arrestin	1219,401977	3,866589723	0,362383755	10,66987599	1,41E-26	1,83E-25
gir_mul_03464	46,99637376	2,564225809	0,253819396	10,10256052	5,38E-24	5,87E-23
gir_mul_cng	27,2142945	3,149194393	0,312808708	10,06747674	7,69E-24	8,27E-23
gir_mul_27566	22,74058915	-2,74759694	0,274514482	-10,0089326	1,39E-23	1,47E-22
gir_mul_01915	155,4609965	1,314703516	0,132129758	9,950094034	2,52E-23	2,58E-22
gir_mul_pitp	180,0827393	3,032991936	0,311654271	9,731911991	2,20E-22	2,14E-21
gir_mul_plcb	155,0441051	2,791593567	0,289470302	9,64379953	5,22E-22	4,92E-21
gir_mul_cpo	17,29034892	3,928458224	0,417380586	9,412172852	4,86E-21	4,39E-20
gir_mul_32930	180,4307729	1,983943952	0,219118687	9,0541979	1,38E-19	1,10E-18
gir_mul_trpc-2	171,1631193	2,039640436	0,226535226	9,003634769	2,18E-19	1,72E-18
gir_mul_32587	42,60407961	1,870356198	0,210191303	8,898351963	5,67E-19	4,30E-18
gir_mul_03129	91,33373814	1,696187008	0,198028285	8,565377465	1,08E-17	7,25E-17
gir_mul_22592	10,476043	3,437552535	0,405064243	8,486437888	2,13E-17	1,40E-16
gir_mul_02471	65,15145919	2,510264763	0,298306044	8,415065036	3,93E-17	2,51E-16
gir_mul_ninag	26,34313799	3,494530778	0,417313461	8,373875054	5,58E-17	3,53E-16
gir_mul_22239	27,17461788	2,557531829	0,309936901	8,251782284	1,56E-16	9,46E-16
gir_mul_rops2	105,9456439	1,98921009	0,247105785	8,050034469	8,28E-16	4,76E-15
gir_mul_cdkl1	56,25077515	2,256276547	0,281728263	8,008697901	1,16E-15	6,55E-15
gir_mul_26028	218,446794	-1,66647494	0,228164079	-7,30384444	2,80E-13	1,36E-12
gir_mul_29449	46,95998175	1,797353617	0,258621477	6,949746164	3,66E-12	1,63E-11
gir_mul_eya	49,22047744	1,243062735	0,180850957	6,873409782	6,27E-12	2,74E-11
gir_mul_21691	123,6667526	-1,25057788	0,186191611	-6,71661773	1,86E-11	7,78E-11
gir_mul_cgs-pde	80,83611096	1,268967496	0,19043059	6,663674666	2,67E-11	1,11E-10
gir_mul_best-a	14,99232324	2,222070983	0,334459468	6,643767625	3,06E-11	1,26E-10
gir_mul_09627	134,2536795	1,318759561	0,200465329	6,578492002	4,75E-11	1,92E-10
gir_mul_best-b	10,65550774	2,492616533	0,381268456	6,537694098	6,25E-11	2,51E-10
gir_mul_21613	606,9636282	1,506971872	0,234703904	6,420736293	1,36E-10	5,29E-10

gir_mul_5ht-r2	9,594056957	2,332000401	0,363876308	6,408772296	1,47E-10	5,70E-10
gir_mul_nanos	5,643734323	2,953092583	0,46102773	6,405455446	1,50E-10	5,82E-10
gir_mul_stim	85,48408376	-0,79231876	0,123749135	-6,40262059	1,53E-10	5,91E-10
gir_mul_59sley	29,83014045	1,606904249	0,258686369	6,211785551	5,24E-10	1,93E-09
gir_mul_26962	218,8724419	-1,11154178	0,199291578	-5,57746489	2,44E-08	7,69E-08
gir_mul_09720	59,11569349	1,923537069	0,345687572	5,564380159	2,63E-08	8,24E-08
gir_mul_5ht-r1	115,2845869	1,385547654	0,253188452	5,47239671	4,44E-08	1,36E-07
gir_mul_10538	31,41730386	1,475491244	0,269959545	5,465601307	4,61E-08	1,40E-07
gir_mul_nhe-rf	31,67907171	-0,88489192	0,161939745	-5,46432821	4,65E-08	1,41E-07
gir_mul_06952	21,86710388	-1,20848865	0,221531452	-5,4551561	4,89E-08	1,48E-07
gir_mul_33093	154,7906008	-1,23909557	0,227561151	-5,44511032	5,18E-08	1,56E-07
gir_mul_gpcrP-2	33,78783545	1,273151247	0,238910671	5,328984429	9,88E-08	2,90E-07
gir_mul_six-1-2	67,20604741	1,5040641	0,28945886	5,196123895	2,03E-07	5,77E-07
gir_mul_08711	26,72871992	1,943761616	0,374273522	5,193425397	2,06E-07	5,84E-07
gir_mul_dagk	96,54156742	0,980776148	0,191054603	5,133486106	2,84E-07	7,90E-07
gir_mul_pctaire	50,12166527	0,941435037	0,184786044	5,094730199	3,49E-07	9,56E-07
gir_mul_04256	53,29613955	1,631259653	0,320244882	5,093788359	3,51E-07	9,59E-07
gir_mul_12518	37,15674776	1,813238835	0,357787882	5,067915727	4,02E-07	1,10E-06
gir_mul_eag	7,646046051	1,681468287	0,341841438	4,918854474	8,71E-07	2,29E-06
gir_mul_20798	23,57832236	-0,89148189	0,183450321	-4,85952755	1,18E-06	3,04E-06
gir_mul_cdh23	36,58039101	1,710285381	0,358529285	4,770280851	1,84E-06	4,67E-06
gir_mul_19866	76,79561764	-1,22151662	0,260238695	-4,69383163	2,68E-06	6,68E-06
gir_mul_meis	36,5592134	-0,77475492	0,168651417	-4,59382398	4,35E-06	1,06E-05
gir_mul_04136	42,26311419	0,719549788	0,157997835	4,554174984	5,26E-06	1,26E-05
gir_mul_09268	106,6728498	-0,82071123	0,185001523	-4,43624041	9,15E-06	2,14E-05
gir_mul_sp6-9	5,157939439	1,84350102	0,418992839	4,399838967	1,08E-05	2,51E-05
gir_mul_egfr-1	76,37280148	1,270530878	0,292876898	4,338105493	1,44E-05	3,28E-05
gir_mul_nkx1	10,96681254	1,317410361	0,316576845	4,161423626	3,16E-05	6,93E-05
gir_mul_foxq2	23,76287645	1,228827556	0,299241528	4,106473997	4,02E-05	8,67E-05
gir_mul_09132	45,08986362	1,033710864	0,25482316	4,05658129	4,98E-05	0,000106523
gir_mul_crf-r	8,57443292	-1,44907204	0,372590428	-3,88918216	0,000100583	0,00020849
gir_mul_dlx	30,25028837	0,886449891	0,230125599	3,852026439	0,000117144	0,000241193
gir_mul_myoVI	170,7589507	0,985305462	0,257926967	3,820094788	0,0001334	0,000273018
gir_mul_20488	75,25586849	0,854117859	0,231293973	3,692780444	0,000221816	0,00044162
gir_mul_34512	46,94984114	0,786698942	0,214373596	3,669756714	0,000242781	0,00048149
gir_mul_23388	165,5561636	0,618445296	0,170517357	3,626875921	0,000286871	0,000564198
gir_mul_arrb-2	20,83311998	1,078959868	0,299637466	3,600884372	0,000317137	0,000617402
gir_mul_soxB	45,71267293	0,770209042	0,21583788	3,568460927	0,000359084	0,000692488
gir_mul_ncx	298,795357	0,601895752	0,168702651	3,567790719	0,000360004	0,000693826
gir_mul_34343	51,51057136	1,21015356	0,345036444	3,507320981	0,000452643	0,000859434
gir_mul_20047	165,1661882	-0,79101324	0,236607229	-3,34314908	0,000828334	0,001518365
gir_mul_10513	105,5394791	-0,4850363	0,147655983	-3,28490786	0,001020157	0,001846857
gir_mul_rgs6-7-9	369,1755392	-0,60541593	0,196150783	-3,08648233	0,0020254	0,003491912
gir_mul_13049	115,453371	0,70666717	0,232185811	3,043541587	0,002338111	0,003995194
gir_mul_pis	30,76413145	0,5035529	0,177468107	2,837427574	0,004547866	0,007492037
gir_mul_gls	6,76296143	1,003991999	0,369907099	2,714173372	0,006644138	0,01065975

gir_mul_unc5-2	134,7105899	0,572700026	0,216362791	2,64694323	0,008122299	0,012850204
gir_mul_gpcrP-1	22,98169272	-0,74299585	0,285879363	-2,59898387	0,009350017	0,014649312
gir_mul_nf-i-a	71,71641373	-0,4365149	0,169210541	-2,57971457	0,009888201	0,01541396
gir_mul_gna-o	125,287335	-0,45326261	0,176339941	-2,57039108	0,010158376	0,015811063
gir_mul_otxA	24,95381608	0,519141157	0,202761123	2,560358461	0,010456425	0,016250278
gir_mul_pcdhP-4	143,3232504	0,819222958	0,323095306	2,535545836	0,011227223	0,017404178
gir_mul_atp13	110,0876771	0,743833874	0,296533629	2,508430079	0,012126897	0,018704506
gir_mul_pde1c	64,15482645	0,602718968	0,244694395	2,463149876	0,013772233	0,021104609
gir_mul_robo-c	204,8522226	-0,42229491	0,175924591	-2,40043136	0,016375762	0,024760989
gir_mul_smad6-7-2	153,1902925	0,671217747	0,280569645	2,392339153	0,016741364	0,025251695
gir_mul_15984	125,5030651	-0,46706875	0,196285392	-2,37953902	0,017334307	0,026043823
gir_mul_abl	107,62912	-0,55251569	0,232356772	-2,37787644	0,01741266	0,026148762
gir_mul_23489	51,85948583	0,390221034	0,169559475	2,301381465	0,021370079	0,031537024
gir_mul_01362	33,60860698	-0,5383359	0,23913892	-2,25114299	0,024376482	0,035614689
gir_mul_12199	212,7282621	-0,41072224	0,182896231	-2,24565723	0,024725972	0,036056754
gir_mul_klf	7,544320545	0,845774182	0,388392534	2,177627294	0,029433796	0,042538547
gir_mul_hcng	55,25547466	0,522300903	0,249034107	2,09730671	0,035966433	0,05109095
gir_mul_pmca2	61,36762939	0,562053954	0,272040447	2,0660676	0,038822097	0,054893802
gir_mul_ddc	34,75065836	0,669651637	0,330397737	2,026804548	0,042682405	0,05988394
gir_mul_23244	64,73758393	-0,31050278	0,155849615	-1,99232308	0,046335621	0,06454499
gir_mul_gna-q	160,4617879	-0,60319471	0,308170529	-1,95734068	0,050307428	0,069565917
gir_mul_07486	5,299382149	0,714619787	0,373476246	1,913427677	0,055693324	0,076265735
gir_mul_myoVIIA	315,0264142	-0,51983973	0,271816368	-1,91246662	0,05581637	0,076393376
gir_mul_16656	45,1680505	0,393490423	0,208086145	1,890997704	0,058624651	0,079952163
gir_mul_qdr	72,86599541	0,29827609	0,164905692	1,808767705	0,070487099	0,094796961
gir_mul_23802	2,222907563	0,92408606	0,545904278	1,692762078	0,090500764	0,119296461
gir_mul_11992	39,69416278	-0,36942507	0,223260083	-1,65468483	0,097988446	0,128286407
gir_mul_maguk	411,1347156	-0,35124924	0,216342864	-1,62357674	0,104466172	0,136142607
gir_mul_sk3	59,21709376	-0,32745307	0,209860833	-1,56033437	0,118680884	0,152902363
gir_mul_14918	54,19433898	-0,41233744	0,266279579	-1,54851318	0,121498792	0,15614017
gir_mul_fzdP-1	89,53965755	-0,37041871	0,244088674	-1,51755797	0,129125868	0,164976275
gir_mul_17028	27,13839907	-0,50672288	0,345337507	-1,46732653	0,142287251	0,180367427
gir_mul_ip3r	43,81250907	0,390008037	0,284357017	1,371543564	0,170205573	0,211569726
gir_mul_erm	10,55513521	-0,42443806	0,311750632	-1,36146656	0,173366294	0,215237475
gir_mul_13405	182,2836564	0,375765037	0,279968738	1,342167841	0,179541581	0,221857708
gir_mul_nol1	4,601279158	-0,56519912	0,461207723	-1,22547627	0,220395827	0,26746813
gir_mul_27330	77,46231814	-0,22215562	0,188109558	-1,18099058	0,237606464	0,286711716
gir_mul_07357	109,7717902	0,202363286	0,178190191	1,135658951	0,256099327	0,306919817
gir_mul_08883	53,15048942	-0,19539698	0,184944396	-1,05651745	0,290731875	0,34163218
gir_mul_00978	145,918286	-0,14027943	0,144472733	-0,97097515	0,331560655	0,38475555
gir_mul_tyrosinase	80,20999349	0,363321856	0,3749215	0,96906114	0,332514685	0,385571536
gir_mul_03496	152,8943132	0,196561598	0,228270109	0,861092147	0,389187294	0,445904488
gir_mul_34307	8,117191642	0,242701555	0,299431483	0,810541202	0,417629195	0,474599684
gir_mul_31312	6,404182967	0,3177472	0,397092867	0,800183601	0,42360443	0,480856727
gir_mul_30263	20,04872448	-0,17171912	0,258585209	-0,6640717	0,506644424	0,561841616
gir_mul_fzdP-2	300,6108598	-0,13170606	0,211085481	-0,62394657	0,532662677	0,587515274

gir_mul_unc5-1	129,3279128	-0,20755378	0,339350881	-0,61161998	0,540789218	0,596264726
gir_mul_08086	159,2969331	0,085769052	0,157369796	0,545015973	0,585742532	0,639407863
gir_mul_ttbk	62,05214509	-0,12814921	0,235825463	-0,54340701	0,586849602	0,640388951
gir_mul_ddryk	70,767236	0,143008463	0,269757374	0,530137361	0,596016697	0,648091732
gir_mul_04906	72,48407011	0,103047009	0,257367492	0,400388598	0,688870321	0,732475741
gir_mul_29424	283,6706717	0,081119195	0,219810989	0,369040672	0,712097409	0,753005654
gir_mul_17151	33,54468827	0,091592117	0,274808549	0,33329428	0,738912157	0,775756821
gir_mul_25626	382,0672517	-0,05953974	0,230133524	-0,25871825	0,795852635	0,825666892
gir_mul_transferrin	7,261241197	0,103932345	0,459706823	0,226083973	0,82113611	0,847891301
gir_mul_sans	10,06292446	-0,07355591	0,331379764	-0,22196863	0,824338307	0,850340925
gir_mul_ovo	15,00530907	0,07150524	0,340919655	0,209742204	0,833868884	0,859595221
gir_mul_09274	60,0813642	0,037971931	0,250392452	0,151649663	0,879463261	0,898162812

Table S4 - Contig annotation of genes used during the study

gene	Species	Best blast hit	evalue	A.primer	B.primer	C.primer	Sequence
arrestin	G. multidiverticulata	Beta arrestin [Fasciolopsis buski]	1,00E-129	TCAAAACGCACAGTGCATAA	TCTAAACCGGGTCTGATGAAA	TGGAAGAGAGGATATGGATGAA	CCACTTTATTTCAAATTTAATCAATTTTGTCACTATAACACGATTATTGTGTCGAAAATAATCTAGAATTCAAAACGCACAGTGCATAATAAAATGTAAGTTATTGCAAAGAC<br>AGTTCAAGAATCTAAACCGGGTCTGATGAAACTATTTCTTCACCCAATTTTAGTTTAATTCTCGAAAAATTTTCGATATTTATTTCTTCAGGAATGATAGATGAATCCTCTTTTT<br>TAGGTTCATGGATAAGTGTAAAAGGAATTTCCATTTCCGTATCTCTGCCTCCGAAGCCAGTAATTAATTTAATTCTTATAGAATATTGAACTATTATTGCCATTGTACTCTTTT<br>TCCTGTAATCTTTCACTAAAGTTGATGTGGCAAAATTTGTGTCTTTATGCTTCATTTTTCCGTCGAGAGCTAAACCACTTTGAGATCTATTATCGTTTAATGAAGGACGCATTT<br>TAAAGTTTTGCGACCAACCAGTCTTTCCAGGATCAATTGGCATTCCGCTTGTTGTTTCTATCTCAAAAATGACAGATTTATATGTCTGAGGTCGGAATAATGTTAACTCGACA<br>ATTTGTAAAATCTGTAATTTTATTCTTTTGACACTGTTACTTGAATTATTATCAATAACTAGATTTAAAGGTATTGTTTCGCCGTGAAAAAATACTTCTTTTGGAAGTGATGCTT<br>CTATTTTAATAATACCTGAATGAATGTGAAATGGTTTCTGCATTTCTTTGGAATATGGTTGAGATTGCGGATGTATAGAAGCAGTTGTAAGTCTCCTAATCACAACACTGACA<br>GAGTTGTGAGAATGAGCTTTATCATCTTTTGATTCTCCAACAAACGCTTTTAAGCTGTATTCAATACCACAAGGTTGGTTGGTTTGGTCTTCATCTGCAGATAGTTGCAGCAT<br>AATTGAACAAGGAATATCATGAGGTAAGTAAAATAAGAACGGATACGATTCGTTATTAAATTTTTTCATTAATTTTTCTTGAAGTGGTGTTAATTTGTCAATTGCTCCATCTA<br>AAGGAGGATAAATCTGCTCGCATTGAAGATAAAAATCTTTCTTAAAAGTTACACCACTTAAAACTTCATCCATATCCTCTCTTCCATATCTAAATAAGCAATGAATTCTTATAA<br>AAACTTTCCGACCCTTTATATAATTTTGATCAACTGTTATAACACCATCAATAGGATCAATATTAGTCAGGTCATCAAAAAAATCTCTACTTCCAAGATATAAAACAATTTTAT<br>TATTGAGACTGGATTTTTTGAAAATACTTTTCTGT
arrestin	G. dorotocephala	Beta arrestin [Fasciolopsis buski]	5,00E-131	TAAACCGGATCCGATGAAAC	TCATGGATGAGGGTAAAAGGA	AAGGGTCGGAAAGTTTTTATCA	GAAAGTTTCAAATTTATTTCAAATTTAATAAATTTTGTCACTATAACACGATAATTGTGTCAAAAATAATCTAGAATTTAAAACGCATAGTGCATAATAAAATGTCAGTTTTTG<br>CAAAAACAGTCCCGGAAACTAAACCGGATCCGATGAAACCATTTCTTCACCCAATTTTAATTTAATTCTCGAAAAATTTTCGATATTTATTTCCTCAGGAATGATAGATGAAT<br>CCTCTTTTTTAGGTTCATGGATGAGGGTAAAAGGAATTTCCATTTCGGTATCTCTGCCTCCGAAGCCAGTAATTAATTTAATTCTTATAGAATATTGAACTATTATTGCCATTG<br>TACTCTTCTTCCTGTAATCTTTTACCAAAGTTGATGTGGCAAAATTCGTGTCTTTATGCTTCATTTTTCCATCGAGAGCTAGACCACTTTGAGATCTATTGTTGTTTAAGGAAG<br>GTCGCATTTTAAAGTTTTGAGACCAACCCGTCTTTCCAGGATCAATTGGCATTCCACTTGTGGTTTCTATTTCAAAAATAACAGATTTATATGTCTGAGGTCGGAATAAGGTT<br>AACTCAACAACTTGTAATATCTGTAGTTTTATTCTCTTCACACTATTACTTGAATTATTATCAATAACTAGATTTAAAGGTATTGTTTCACCATGAAAAAATACTTCTTTAGGAA<br>GTGATGCTTCTATTTTAATAATACCTGAATGAATGTGAAATGGTTTCTGCATTTCTTTGGAATATGGCTGAGATTTAGGATGTATAGAAGCAGTTGTAAGTCGCCTAATCACA<br>ACACTGACAGAGTTGTGAGTATGAGCTTTATCATCTTTTGATTCTCCAACAAACGCCTTTAGACTGTATTCAATACCACAAGGTTGGTTGGTTTGATCTTCGTCTGCAGATAG<br>TTGCAGCATGATTGAGCAAGGAATATCATGAGGTAAGTAAAATAAAAACGGATACGATTCGTTATTAAATTTTTTCATTAATTTTTCTTGAAGTGGTGTTAATTTTTCGATTG<br>CTCCATCTATCGGAGGATAAATCTGTTCACATTGAAGATAGAAATCTTTCTTAAAAGTTACACCACTCAAAACTTCATCCATATCCTCTCTTCCATATCTAAATAAGCAATGGA<br>TTCTGATAAAAACTTTCCGACCCTTTATATAATTCTTATCAACAGTTATAACACCATCAATAGGATCAATATTAGTCAAGTCATCAAAAAAGTCTCTACTTCCAAGGTATAAAA<br>CAATTTTATTATTGAGACTGGACTTTTTGAAAATACTTTTTTGCGTCATGTTACATTAAC
dlx	G. dorotocephala	distal-less [Schmidtea mediterranea]	0	CTCTGCGGGATCAGAAACTC	CCTTACCAGACTCCCCGACT	ATTGACCCATGCCCAAATAA	TTTTACAGTGTTGTGTTGACAACAATTAAATTACAGAACAGTTGTTCTTCGAATTAATTTGAGCAATTTTCCCTCTGATTACAGGTGTCCAGTGACTTCAGCTATAATCATCGT<br>GTTACAACATAGTCTCAACCACCTCCACGCCAGTCGTAATTGCATAATCATTGAAGATGATCTCTGCTATGTCTCATTGCAATTTACAAGCTTCAAATGATCTATTTGAACCAC<br>CCACAGCCTCTGCGGGATCAGAAACTCATTCCCAATCTTTACCCTACCCCATCACTTCATCAGGGTCTTACGAATTTATTGATAATACCATTTTTAGTTCCGCCTTACCAGACT<br>CCCCGACTAAAAACGATATCAAACCTTTACAAGGTCTTTTACCTCCTTTTGGTTTGTCTAAATTAGATGCGCAATCTTTTTATGGAATCTCCAATCAGTATGATATGCTTTATC<br>CCAATATGCCAACCAGACTCTACGCGCCGTCAGATACTTGTAACGAATCCAGTCTCTCGCTCACAAAGACCTCCCTATTCAGTAATCCATCAAGTTTAGCATACGCTTCACTC<br>GGTTGTGACATCATTCCAGTTTCATCTCCCAACGAAATTCTTCAAGAAGGATTCGGTAATAATAGTTCTCATAAAAAAGATCATCATTCAATTTCTGGATTAGGGATTTCCAC<br>TTCAAACAATCAACAATTAATGCCTCCAATGACAACTCCTGGTTATAGAAACATTAATCAACAATTGAATCCCGGCTCAAGCAGTTCAGTTATTTTAACTCGTAATTTCAATTC<br>CCCATACGGTTCCCAACAATCGACTTCGGCATTGGGTCATTTGCATCAAAATTATCCGACAAATTCTCACAATTACATGTCCAGTCAAACCATGGCAGCTGCAGTTTATAATT<br>ATCAACATAATATGGTGTTCCCGAGTGGGCCAGTGACCCCTCCATCCATGAAAGAAGATATTCAACGGGAAGATCAAATTCGAGTTTCTGGGAAGGGTAAAAAAATCCGA<br>AAACCGCGAACCATTTATTCGAGTTTACAACTTCAACAGTTGAGTAAACGATTCCAAAGAACTCAGTATCTCAGTTTACCAGAAAGAGCCGAACTTGCGAGTTCTTTGGGAT<br>TAACTCAGACTCAAGTCAAAATTTGGTTTCAAAACCGTCGTTCGAAGTACAAAAAACTGATTAAACAGGGTCAAGATCCGAGTATTTTGATGAATGGAGAGTTTAATGATA<br>GTATGGATGAAATGACGGAAGATCAGATTGATGATGAAAATTGTATAAAACCGAAAGCGGAAATGCTTTTAACAAACGATCCAAATAACACACGGGGCGATAGCTCCGAT<br>ATCCCAACGGAAACGTCAGAAGCATCCAGTCCCGTTTTGAAACAACAGCAAACTGTAACTTCAATCCCTTCCAGTGGATCTCCTGGAAACAAATACTCACTCGATACAGGAA<br>TGGTCTCACACTACCATAGCGGAATAGATCCATATCCAACTCAAATTGCAAATCAGCATCAACTTGGTTACTACAACAACAATATGAATAACTCGCAACTCCCGCTGGGAAC<br>AGGTGGGTTTATAGATTCAAGTTATTTGGGCATGGGTCAATCCATGAATCATTTGACTGCCCAGTCCGGGTATCAGTATTATTTAAATTCCTACAACGCTAATTCCTATCCCA<br>CTTGGTACGCATCAGAATGTTTACTCAATAATGTTGATTTTAAATGAATATTTAATTACCCATTACAAAAAAAGGAAAAAAAAACAATCATTCATTGCAATGATTTTGTAATA<br>TTAATAATGTAGTTTAGTAGCTTGGTTTTTTTATAAATGGAAATAAAATTGATCGTTTCTCATTCTCGGATCTCTTTTTATCTTTCTCTCTCTCACACTGTCTCTTTCTTTTTTACC<br>GAAAACCGATCAAAGAATGTTTATTTGTTTGCAAAATAAACTGTAAAAACTCAATTTTCTTGTTTTTCGAAAACTCGAGAGAATGGTTCCAGATTTTGTCATTGTAAAAA
eya	G. multidiverticulata	eyes absent protein [Dugesia japonica]	0	CCGCATCAGTAACAGCTTCA	CTGTGCCCTTCATCAATCAG	AGAGCCACGATATCGGAATG	TGAAATTCGAGTATTATAAAAATCACTTCTCAATTTTTCATTTAACATTTGTTTTTCATTTAATTCAATCAATAAAAGAAATTCTTTAAAAAAAAATTTCCTCAATTAAATTAGA<br>AATTTAAATCAATTATTTTTTTTCAAGAATACCTAAATGTTCGCCAGTGGAGCAACTACTGCCTCTTTGTTTGGTTCAAGCCAAACGATTCCCACAACTTTTCCCTCATTTATGT<br>CATCATATTGTCTCAATAATTCTGAAGTTGCCCAACAATTAGCCGCATCAGTAACAGCTTCAACTAGTTCTTATTTATCAAATTACTCGGATTTTGAAGGTATTAATAGTACGG<br>CGTGTTTAGAGGCAGCAACTAATCCAAATGAGGCTCTAATTAATAGTCAAGCGGAAAGTTTCTTTAACTGTGCCCTTCATCAATCAGGACTTTATGGACAGCCATACTCAAT<br>GAGCAGTTACTTGTCAGATATGCCTCCAACAAGTGATGAGAATACTCTTTTCTCAAGCAATTTCTATAATGGTCTCACAGCAAGTTATTCCTCAGACCAAGCCCATCAACAAC<br>GTAATTCAGCCTACGTATATGCATACGGTTATTCTCCTTACATATCCGCCATGTATGCTGGAATTCATTCTCCATGTCAGCAAATAATGAACAGTATGGGATCAGTTGTTAAC<br>AGTCGGTCTTATTGTCCACCAAGCAATCCTTCAATTCCTTCAACCACTCAAAAGTACCAATTGATAAACTTGCCCCCGGCTCTTCCATCGACAGCAGGCAATGCATCGGTAAC<br>TGAGACCGCAATTTCGTTGAAAGCATCCGAATCTCCGGGACTGTCGAAAGACGATTCTGAAAGTCAATCGAAATCTCCGCAAAGTTGCAATGGGAATTCAGAAACAAATAA<br>TAATGGACCAAAATTACAAACAGAAATTGTGAGTGAAAACGAACTTGAACGAATTTTCGTTTGGGATTTAGATGAAACAATTATAATTTTCCATTCACTATTGACAGGAATT<br>TATGCTCAAAGATATCAAAAGGATGCATCGACAGCGGTGGCTCTGGGACTGAGAATGGAGGAACTAATATTTAGTTTGGCGGATAATCACATGTTTTTCAACGACTTAGAG<br>GAATGCGACCAGGTTCACATTGATGACGTTTCTTCGGACGACAACGGTCAAGATTTGAGCAACTATAATTTTCTGGTTGATGGTTTCGCGGGTCCAGCGAGTATTTGTAATG<br>GGGTGTCAACAGTTATCCAAGGAGGTCCTCTAGGATTACCGGGAAATACGAGTGTCCGAGGAGGTGTAGATTGGATGAGAAAATTGGCTTTCAGATATAGACGCATTAAA<br>GAATTATATAACTCTTACAGAAACAATGTTGGTGGATTATTGGGGACCCAGAAAAGAGAACATTGGCTCCAACTGAGACAAGATATAGAAACACTCACAGATAATTGGTTA<br>ACTCTAGCTTTAAAAGCGTTAACTTTGATTTCTCAAAGAACGAATTGCGTCAACGTTTTGGTCACAACCACGCAATTAGTTCCGGCATTGGCAAAGGTTCTTTTGTACGGATT<br>AGGAGGAGTTTTTCAGATTGAAAATATTTATTCAGCAAATAAAATTGGAAAAGAAAGTTGTTTTGAACGGATATCATCGAGATTTGGAAGAAAAAGTACGTATGTAGTAGT<br>TGGAGACGGAAAGGACGAAGAGACCGCCGCTCGGCAGGTAAACTGGCCGTTCTGGAGAATAAGCTCACATTCCGATATCGTGGCTCTTCATCATGCACTGAGTCTTGGTT<br>ATCTTTAATCCCACAAAACACTCACAAACTACTTTTTTTTAGTTTTTTCCATTCATTTGAAAATTTAATAAAATTATTTAACTG
foxq2	G. multidiverticulata	foxQ2 [Schmidtea mediterranea]	1,00E-173	CATTAACCCCGTAGATCTGTTG	GATCGCTCGATGCTTGAAT	CACCGACAATTTCCCTATGA	CAGAATTTATTAGTCTTTTTTCTAAAAAAAAAAAGAAAAATTATTGAAATTGATCAATTTGATGTATATTTGTAACACTTTTTAACATTAACCCCGTAGATCTGTTGGCGATCG<br>CTCGATGCTTGAATTCTAATATTTGATGAACTTGATTCTCTCGACATAAACCTGTTGGTAACTGTCTGGCGAATCTCATTCCATGGAAAATATTCAGAAAATTTAAATGGGAA<br>AAGAACTGATTGTAGAAAACATCTTTAAAGGGGATTTTCTTTGATACCGGATTGAGAATATTTTCTATATCGAATGTTCTCGTATTTTGATTTGGTTTATTGAATTTTACTTTTT<br>CATCATCTTCATTAAATTGAGACCAGAAATACGGATTGAACATGGAATTTGGAATGTTTGGGATTTCATTTTCTGATTTATTATGCATAAATGAACTCATTTTATAATGGGAT<br>GTGTCGGAAATTGAATAGTTTGTACTTTCATTTTGCGAATTTATTGACCATTCATAGACCTTTGGTGAATGTACTGGTGATGGTGATGGCGTATCATCTTCATCTGGACATGC<br>GAGCCCAAGAGCTCTACGAACTTTTCGTTGTGCTCTTCTCCGTCGAAAGTCACCCCTTTTAAAGTCTTCAATGTTAGCTGGATGGATTCCCCAATAATGACCTTTTCCATTTGA<br>TGATCGACCAACTTTTACAAAGCAGTCATTCAAGGAGAGATTGTGTCTGATACTATTTCTCCAACCGGGACCTCTCGTTTGAAAATAAGAATAATGGTCTTGAATCCACTGA<br>TAAATATCAGACAAAACCATTTTTTTATCTTTTGTACTTAAAATCGCCATCGATATGAGACCAATATAACTATAATTGGGTTTAGGATCGTCTTGCATGGTTTTCTTACAAAAA<br>TCACCCATAACCGGATGAAAGGTTTTCAATAATGGTTGTAATGAAAGCATAGAGCTTTGAGAAAAGAGATTATTTTGTGGAGTGCCAGGAATGACAGTCAATGGTAGAGA<br>GGATTGAATGATTTTCGAAAGAACATTTGAATTGTTTAATGAATACAAATTGCTTTGGGAAGAATTGTTAATGAAAGAATTTTCCATTTTCCAACGGATAAATTTAAAGTCAA<br>CCAGTCTATTAAATAAGTATCACTGTTTTTTTCAAGATTTATCATAGGGAAATTGTCGGTGTATGTTTATGTGTGTGTATCAACAGAGGTATTGACAAATAATAAACATTTTCC<br>ATACATACTGTGTTTTGG
klf	G. multidiverticulata	klf [Schmidtea mediterranea]	0	CACCCCAAAATTACAACGAGA	TGAAATATGGGTGACTAATTTCG	CGATTTCAAACGTCACACCA	TGAGACTAAATTGTGATACCCTGATTTCTGAAAACAATTTCCGAGTTCTTTACAACGATAATATGATCACACCCCAAAATTACAACGAGAATTTCTGTGATTACGATAATGAA<br>ATATGGGTGACTAATTTCGAAAATAATTCAGTCTCCTATTCCAATATCCCCGAAGGATCATGTTCGACAGTATCAACAAATACCGACGGATCAATCATTTCGTCTTCATCAAA<br>CAACAAATCCAACTATGATGAAACCGATCTCAGTGCGTTTTATAATTCCTCAAATTTCTCCTTTGGTAATAATAGTTTCACTTTTGATAAAGACGGCTTACTTGATTTTGAGTT<br>CATCCTCGACAACAGAATCACGAATGCCGGTTCAATAATAAATAATTATCAAGATAAATTAAATTCCATCTCTAATCTTAATCAACAAATCAAGTATGAACCATCCTCTGTTA<br>ATCATTCAGATAATTATAACTTCACCTTTCCCACTTCATCATGGTACAACAGGCTTATGTATCCAGATTCCATACAAATTAATGAAAAGTATAATCAAGGTAATGATGAAAAT<br>TATTCGATAATGTCAGAGTATTCTTTTGAGAACGAACAATATTCTTTACCTTCGGATCCAAATGTTGAAAACATCTATTTACTTAATAAATCAAATGTTCAAGAGTCTATTTAC<br>AATTCTCTTCAAAACAATTTCGAATTAGAACAAGATTTTAAAATTAAAAAAGAGACTGTTTTGAGACCGGATTTGTACACGTCTAATGGTTATTATTATCACGGATCTTGTGT<br>TAATAATTCCCAAATAAATAACCATTTGAATATCGAAGATGAGAGTATCACTTCTCTTAATGGAGGAAGTACCAAATACCTTCCTTTCATTGCGGACAATTGTTTGGCTAATA<br>AATTACCCCCTTTAATGCCAGCTCCAAATGAAACGAATTCTTGTTTAATTAATTCATCGATATCAGTTGAAAGTGCAACACCCGATAGCACATCATCTAATTATCTACGATCTT<br>CGTTAAAACAATCGTCAAATTCAATGACATCAAATAGACGGGCAAGGAATCGTCGGAAAGTGTCCGTTCACTCTTGCTACTATCTCGGTTGTGATAAATCCTATTCCAAAAG<br>CAGCCATCTGAAAGCACATATGCGAACACATACGGGAGAAAAACCTTATCGTTGTGATTGGTTGGGATGTGATTGGTGTTTCGCGAGGTCTGACGAGTTGACGAGACATTA<br>TAGAAAGCACACTGGAGATCGACCGTTTCAGTGTTCCGTTTGTTCGAGAACCTTTTCTCGGAGTGATCATTTATCTTTACATATGAAAAAACATAATTGATTTCTCTGCAAAT<br>ACTTTGTTTTTGTATGATATATATAACTGATAATTTTAACTCTATTTTGACTTTGTATGTATACAATTACATTTGATGTTAAAGAAAAAGACTCAAAGAATCTTACAAAACATTA<br>GAATTGAATTATATATTTTGGTGTGACGTTTGAAATCGTTAATTTGAAATACATGATTTCTAC

opsin	G. dorotocephala	opsin [Dugesia japonica]	0	CAGCTTGACCCTTGTTGGTT	GTTCTTTTGGTTTGGGTGGA	CAAATGAGCGTCCAGTCCTT	TTATTAACAAGAAAATTTTATTTTTATATTATATACTAAGATTTCTAATGATTAATTTATGAACATCATAAAAATATATGTGATAAATATATTTCGAACATTAAACAAATAATTA<br>TTTGCGCCTATTTCGGTGCTGAATAAAACAGTGAAATAAAAATAAAGAATCTAATGTTGTTACACATTGAAATTTCTATTTTCGACAAATTATTTTTCACTTTCGTAAGCCTCA<br>TTCGTATGACCACTAGCAGCTTGACCCTTGTTGGTTTCGACATTTTGTTTATCATCTTCAGAATCAAGTTGAGACATCGAACTTTCTTTTTCGCTTTGATATTTGCTCGGTTTCG<br>TGGCCGAAGCAACACTTGTTGTATCACGTTCTTTTGGTTTGGGTGGACAACAGCAAATCAAACAAGGGAATTTCTTTTCAAGTTGAATTCTAAATTTCGGATGATTGATCGC<br>GTAAATAAAAGGATTATACATAGCCGATGTTTTAGCAAAAAGTACAGGTAATTCAGCTGTATAAGGGTTTAGGTGATCTCTGCGTCCAGTAAGCGCCATCAGAGCAATAAC<br>TGCATACGGAGTCCAGGATATCAAATATAGAAAGACAATTATCACAGAAATTTTTGCAGCTTGAACATCTGCTTTTTTAGCACCCGTCTTCATGGAAGCTGGATGAGAAGCA<br>TTCATTTTTTGAGCCATCTTCAACATTTCTAGTTCATGAAGTCTAACAGCTTTTACTATCTGATAATAACAAAATATTATTATTGCAACAGGAATAACGAAATTCCCGATGTAC<br>ATTCCTATATTAAAAATTATGTTTCCTTTGGATTGAGTTAGATAATCAAATGTACAGGAAGTTTGAAATCCTTCAGGGACATAGTTTCCAAATCCAAAAAATGGCGGTGTCG<br>ACCATATCAAAGAATAAAGCCAAACGAACACGAGCATTATTATCGCTCTTTTAATGGTCAACGATTTCATAGTTTGAAAAGGTTGAGCGATAACAAAGTATCTGTCCAACGA<br>AATAAGTGCCATTGTATTAATGGATATAAATCCGAAAAGACCCCCAATGAATCCGTATATTTCACAAGTTAGTTTTCCCCAAGCCCAACGAGTGTTAAAACTCGAAATAGTT<br>AATAGTGGAAAGCCATTCACTGCTGAAAACGTCAGATCTCCGATCGCCAAACTCATAATAACATATTAGGAGGGGTCCTCAAACTTTTCGCACTAGCAAATATATAAAGTAC<br>TAGAAGATTTCCTAAAACTCCTGCTATTCCGACAAAGGAAATATAAACTCCGACTAAGTAATGATACACTTCTGGAACCAATTCGAATTCTCTCCAGTAAGGATGAACGATG<br>GATTCGAAATCAGAATCCCAATGCCATGGAACCTTCGAAACATTTGCCGTCTTATTTTCTATCAATTTTTGTCCTATACTTAAAAAGGACTGGACGCTCATTTGTAA
otxA	G. multidiverticulata	DjotxA [Dugesia japonica]	0	TCGGGAATGAACACAGCATA	ACATGATTGGATCCCCAGTT	GGGTCGATCTGACGAACTGT	AAAAAAAAAGATCTCCACATTAGGATTAACCAATGACAGCAAACCAGTAACTGCTATTCAATGCAATAGTATTTCTACCAGTAATCCGCGCTTTGTGATCTAATATTTCAATA<br>AGTCCCTGACAGCTCCAACATTTTAATCCAGCAAAAGTAGCTTAGTCAGAAGAACCTCAACATCCGAGATTTGATCTGATATTACGAGATTAGCACTGTATTAACCAAACCT<br>TATCCCAGCAACTGCTACTCCATTGAAGACTACCAGATAATCCTTCTGAAATACGAGACATATACAGAGCCAATATATCATTCAAACTGGCAGAGAATAATCAGTCTTCAGA<br>TTCCATTCATTTTCCGTATTACTCTTGCGGATCTGTTCAAGTTATCTCTGCGTCTCCACAAAGATTTCGTGACGGAGAAATGTTTCTACCACCGTCTCCAAATGCCGCTTATTT<br>GGTATCCAAATCTTTAACCCCTCAAATTCCTTTATATGGGAGTCAAGATATTAATTCAAGTTTAATGCATCAATCACCGTATTATCCCAGTTTGAATTCCACAACTATTCCTTAT<br>TCAAATTATGAATCAACATCAAGTATTGGGTTACCAAATTTAAATGTAGGCAGTTGTTATGTGGCAGGAATTAATACTCTTGAACGTTATGGGTCGGGAATGAACACAGCAT<br>ATCAGCATACTCCGTATTGGTCAAACAGTTATCATAACATGATTGGATCCCCAGTTGGAGGATTTGGAGCCAGCGCAATGGCTGCTGCCGCCAATACCATGGCCATGGTTG<br>CGGCTAATGGTTCCAGTTTCGGCGGTTCTCCCCAGAGTTATTTGAATCAACATCCAGCCCTTTATCAAGGAACTCAAGTGAAAAAAACCCGACGAGACAGAACAACATTCAC<br>TCGACAACAACTGGAAATTCTCGAACTTCATTTTGAAAAGAACCGATATCCAGATATATTCTTGCGAGACGAAATATCATCCAAGATTAACCTGCCGGAATCTCGAGTTCAA<br>GTTTGGTTTAAGAATAGAAGGGCCAAAGAAAGACAAAAAAACAAACAGAGGACCATTGGATCTGTATCGAGTAACATTAATAATCATGGCAGTTCAAACATCCGCTCAAC<br>GTCTGTCTCTACTCGGATTCCTGGATCTTCTTCTCGAAACAACCCTACAACTAAAACATCTACAGCAACTTCATCTTCAACCAATGTTTCATCAAACCTAAAAATCGAACCTGA<br>ATTGGTTCAGTCAAACAAATCTTCGATTATTAAAACACCAACTGATGATTCAAGGATCAAGAATATAACATCAGAATCTGAATCTAATCAAACCTCTTTCAATGATCATCATT<br>TGGATACAATGATAAAAAATCCTCTAAAATCCAGTCTAAAGAGCAGAAACAGAATGAACGTTTATTCTGATACCCATTGTAATCTTTCCAATAACAGTGACGCTTTAAACCA<br>TGCATCTGTCGAGGATGATTCCGATTTGGGTTTAAGCCGAACAAGTTTCTCATTTATAAACAAAAACTTTAGTTCCGATACCAATGGGAATGGACCTGAAGGGTTTTATAAT<br>TCAGAGATGTTAAGAAATATGTGGCAAAGGAGATCAGAAAAATACAATTCACTTTTTCTAAACTCAGAAAGAAATGGCAACAGTTCTTGTTATCGGCCGTCATATTTGGACT<br>TTATTCCCAAAACTATTGGCGCCAATCAACTACAGAATTATTCCTCCTTAGGTAACGATGGTATACCTTCATTAGGTCGAATGAGCCAATCTTATACTAATAATTGTACTGGA<br>TTGGACCAGGAATCGGAATTATCAGCTGCCGCTGCGGCCTATTACGGACTGAATAATACTAGCACAATGCGTAATTTTGATAGTGATACGTCATCATCTAACGTCAACTGTA<br>ATAAACTTTATTATTCAAACTTATTTTCTTTACCAAATATGGAAATATCCTCTGAGTTTACCAACAATAACAACAATAACAATAATAGTAACAATAATAATGGACCCAATACGC<br>TAACAGATACGGATACGGATAAATTAGAACAAACAAAATATTACACAAATTTGTCCGAACAGTTCGTCAGATCGACCCTAGATGAATATAGCAGCCAGAGCGCCAATGATG<br>ATAACCAGAAACTTTATGGCGATTACAAAAAGATTGATACAGTCACTTAAAACGATTTATCCCTTGGAAATTTATTGTAACTCTTTTTTAACAAAATATCATTAAATACAATG<br>GTTTACTA
ovo	G. multidiverticulata	ovo [Schmidtea mediterranea]	2,00E-67	GACGGAATTGTGAACCCTTT	CAGGAAACAGTTTAAAAATGAACG	AAGGAATTGGGATGTTCTTGA	TGGCTTTGCAAATATTGTTTCAAACCCTTTATTGATTCATATGACTTAAAAAGGCATACAAGAGTCCACACAGGTGTTCAACCTTACAAATGTCAAAGTTGCACCAGAAAATT<br>TAGTCAGCGTTGCTCGTTAGAAAGCCATCAAGTGAAGATTCATGGGATCGAATTGAATTATTTGTATAAGGAGCGCCGAAATAAGCTTTTTCCTTGTGAGATTTGCAGTTAT<br>AGTACTTCCTGTAAAACTACATGGCTAAACCATATTAGTCAAGAACATCCCAATTCCTTATATGCCAAAGAAGAACTTATAAAAGGAAAATACAATTGAATGTATCCC
pax6	G. multidiverticulata	Pax6A [Schmidtea mediterranea]	0	TGCAAGACCCTGTGATA	AGCCATTGGGGGAAGTAAAC	AATAATTGCTCGGTCGGTTG	TTTCAATTGAAACTATTTTTCCATAGATATAAAAAAATAACGAAAATTTTAATTTATTTTTTCAGTTTTCTAGTGGTAATTTTATTTGCTGAGTATTAAAACAAAATAATCAAT<br>TATAATAAATCATTGACTACTGATGAGTTATCCATTAATTTACAAATGTCAAATCATTGGGTTTTTTTTTATAAAAAAAATACACCATTAAACCACATAAACAAAAAGAGAGA<br>GAGAGAGGAGGTAAAAGGTAAGAGTGAGAAATTCTCAAACAAAATTTACGTACAATTTAAATTATATATTGACCATGTTATATCTATATCAAATACGTAAACATTCCATTTA<br>TGTAATACAAAAGTTTTTCTTTCTTTAAAGAGCAAATCGGTTTTTTTTTATGTTTTTTACTAAATGAAAGTCGATTGCAATTTTCAATTCTTTCAATTTCAAGCAACGGGAGTCC<br>AATAATTGCTCGGTCGGTTGAACGCTGAGAAGTCGTAGTCTGGTTTCGAGGAATTCGTTGACATATTGTTGGCAAGAAATCCTGCAGTGTTGATATTATTGTTTGTTTCTAA<br>AGATCCCGGGAACATTGCCGCTGGGTATTTTGGATAACCAATTGTGTCATAACCTCTCATGACTCCTTGGAATAAACTTGGGGGATATACTGATCCATGATTATAAAACGAA<br>TTGGAATTACTCGAAACGTTGGGAAGATCGATTTGTGAACCATTTGATCTGGATTGTAAATTAGCCATGTAGGAATAGGGGGAATTAATTGAAGATTGAGAAAGATTAGGC<br>AAACCGGCTGATTTTGCATATTGATCAGCAGCTGACGCGACTACAGAGTGATACATCCCGGCCGCAGCCACGGCTGAAAGAGATGGAGTTTGCATTCCGACATCCCCAAAC<br>TGATTTCTGATATCATTAATACCGGTAAATCCCATTGAACTTTGCCCTGTCGTTGATAAACACTGTGTATTTCCATTTGTTGTAACATTAGTTTCCGCAGTCGAACTCGTTCCA<br>CTTGAACCGAGCATTAAATTTTGTCTTTGTCTTCGAAGTTTTTCCTCCCGTCTCCATTTTGCTCGACGATTCGAAAACCAAACTTGAATTCTCGCTTCTGGAAGGCTGATTTTA<br>TCGGCCAATTTTTCCCTTGCAAACACATCCGGGTAATGAGTTCTTTCGAATTCTTTTTCCAAAGAATCTAATTGATCTGTCGAAAACGAAGTACGATTTCGTTGGAGTTTTCTC<br>TTCAGTTTTATCCTCATATCATCGTTCTCGCTCTTGACCCCCGACAAATATTCGTTTCCGGGTTCACTCGACGCATTAGATTCAGAATGCGCTATCGATTCTGAATATTTTTCTG<br>ATTTATGTTTGTCAGAACTATCGGCCGTTGATTGAACATGGTAATTGTTGACACCGGAGGTCGTTTGATTTATCGACGCCACAGCATGGGCCGTGCTCATTCCATTAATGAT<br>CCCAATCCCTGTCAGACCCCCATAGGCACCGCAATTGGGGAAAGTTGACGATGCATAGCCATGTGCCGCTGCAGCACTGGAATACCACGCAGCGTGAGCAGCTGCGGTTG<br>ACCAAGGTTGTCCACTGAGTAGGGATAATTTATCATACATTCCAGTCGCAGCTACTAAATGTCGTTGGTTTTCATTTGATAGACTTCGTAAAACTCGATTGATTGAGGAAAC<br>ACTGGGAATGTTGTCTTGATTGCATACCCCTTCTTGTAATAATCTATCTCGAATTTCCCACGAAAAAATCGATGGACATTCTCGTTTATAAGCAGCAATCTTTGATACAACTG<br>AACTAGTTGCTACTCTTGGTTTACTTCCCCCAATGGCTTTCGGACGGATTGATCCGGTTTCATAATACCGACAAAGGATTTTACTAACACAGCCATTAGAGACTTGAAGTATT<br>CGGGAAATATCACAGGGTCTTGCACCAGAATGAGCCAATTCAACGATTCGTTGTCGAGTCGAATCCGGTAACGGTCGACCATTAACAAACATTCCTCCTAACTGATTGATTC<br>CAGAATGTCCTCTTTTCACTTTCTTTTTATTTTTTATTTCATCTTCATTCGATTCGGTCATAGGTTTTGACTGTGTATATTTTCTACTTCAATAACTGAATCAATTTTTTTTAGTGT<br>CAAAAAGGATTTTGTCAATGAAGCGGTTTGATTTATGCCCAGAGATTGAATTTGAGAAGCCAACAATGCGAAAATAGTAGAGGAGTACTAACCGTGTAAAAAAAATTATAT<br>CAGTAAAGTAGCAGAATATTATGATTAATCTTGAGCGACCAATCTAGCATTAATAAATCTGTTAGAAAAAGCAACTCAAGGAGGTGTGTTCAAGCAAAAATTCTGAAACTG<br>GGAGCAGGTGAGTAAAATGATTGGATCGTTTTCAGCACACTCAATCAATACAGATTGGCCAAATGATGCGGAATAAATCGCCCGGCCGTATTACAGAACCGACTCATCTTT<br>TTTTTGGAAAAGAAAATTCTCTCTCCAATCTC
pc2	G. multidiverticulata	prohormone convertase 2 [Schmidtea mediterranea]	0	TCAAGAAACGGGGTTTTCAG	CCTGGCTCATCCTCTCGTAA	GTGGATTTTCTCCCCACGTA	CTTCCCGAATCGTAGAGTTTGCATATCGTTTCTACAAATACGGAGTCTTCTGTGTAGGTGGGAAGCAGATAAATATTAACAACAAAAGCACAAAAATTATCGTTTTAAAATT<br>TCTCTCGAGTTTTTTAGTTCTCATTATTTATTTTTATGAATGATGTTTTTGTGGATTTTAACGACACTACAAATTTATTTAATTAATGGGAAAATTGTCACCAATCAGTGGCATG<br>TAAGTCTTCATCATCCTGGAGACGAAGAAGTAGCTCATAAAGTAGCTCAAGAAACGGGGTTTTCAGTTATACGAAGTCATCTAAAGTCCCCTGGAGAATATGTGTTCGGTC<br>ATAAAGCATTACCTCAAGCTAGCGCTATTCCAAGTTGGGCCCATCACAAAATTCTCCTGGCTCATCCTCTCGTAAAAAATGCAAAACAAGTAGAAGGATTTTTAAGAAAGAA<br>AAGAGGTTATAAGCCGATTAAAAAACAAGCACGTATTCCACTCCCAACAATAAATAAAAAAGGAATTCCAGGCAGAGAAGCATCAGACAACATTAAAAATATGAATTTACC<br>AACTGATCCACTATTTTCTAAAGAATGGTATATTCGTAACACTGGACAGGCAGAAGGAGTAAAAGACTTAGATCTTAACGTTCTTTCAGCATGGGCTCAAGGCATTACTGGT<br>AAAGGTGTTACTACTGCAATTATGGACGACGGTATAGATTATCTTCATCCGGATCTGTCTAAAAATTATAATGCTGAAAGTAGCTATGATTTTAGTAGTAACGATCCTTATCC<br>ATTTCCTCGTTACACCGACGACTGGTTTAACAGTCACGGAACGCGATGCGCAGGTGAAATATCAGCTGAGAGGGACAATGGAATCTGCGGTGTTGGAGTTGCATATGATTC<br>TAAGATCGCCGGATTACGAATGCTTGATCAACCATATATGACGGATCTAATCGAGGCTGCTGCAATGAGCTATGACAGGGATAATATTGACATTTATTCTGCAAGTTGGGG<br>GCCAACAGATGACGGGAGAACGGTAGATGGACCAAGAAATGAAACGATGCGAGCAATTGTTGAAGGTGTTAATAAAGGCCGGAAAGGTAAAGGAAGCATATATGTATG<br>GGCTTCTGGAGATGGAGGAGCTGCAGATGATTGTAATTGTGATGGGTATGCAGCGAGCATGTGGACCATAAGTATAAATTCAGCTACTAATGATGGAGAGACAGCGAGTT<br>ATGACGAATCGTGTTCATCAACTCTTGCATCTACATTTTCCAATGGCAAGGCTTGGTTTAGTCTTGATGCAGGAGTAGGAACAGTGGATTTATATGGACAATGTACACTTCA<br>TCATTCCGGAACATCAGCTGCTGCTCCTGAAGCTGCTGGAGTGTTTGCTTTAGCTTTAGAAGCTAACCCAGAAATTGGGTGGCGAGATATGCAACACTTGACAGTTTTAACT<br>AGTAAAAGAAATCACTTACATGACAGAGACCGCCGACATAATTGGACAATAAATGGAGCAGGACTGGAATTTAATCATCTCTTTGGTTATGGAGTTCTTGATGCTGGAGAT<br>ATGGTTCGAGTAGCTAAAAGATGGGTTAATGTCCCAGAAAGATTTCACTGTATAGCAGGCCAAATTGATAAGCGCTACCGATTTAACGTCAGCAGAGCTCTAATATTAGAA<br>ATCGATACTGATGCCTGTAAGTCAGACGCTGGGCATCGAGTCAACTATTTGGAACATGTCCAAGCATTTGTTTCAGTTAAAGCTACTCGAAGAGGAGATATAACTATGTTTC<br>TTACCAGTCCTATGGGTACAACTTCAATGATTCTTAGTCATCGACCAAAAGATGATGATTCTTCTCGTGGTTTTACTAAATGGCCATTTATGACAACGCATACGTGGGGAGA<br>AAATCCACGTGGTAAATGGCGATTAACAGTAATGTTTGAAACAAAAGAGCCACTGAGTCAAGGATTATTTACTGAGTGGGTTCTTGTTTTACATGGAACACAAAATCGTCC<br>GTACGATAAATTGTCGAGTTCATTGGAAAAAATTATACCGAAACTTGGAGTCGTGAAAAGACAACATGCTTCGAAAAGATTCATGCAATAGGAAAACTAAAACTTGATTAG<br>TTTTGACGAAATGTGACATTCGAGGATCTTAGTCTGACTGAAGATTTTGTGATCGTATTTTTGTAAACTTTTGTCCAGTAAGATTGTGAAATATTGTTATTACATTAATATCAA<br>AGAATAAATATTTGTCTTGTATTTCATAATCAAGTTTGATTAACTTTAAATCTCCCAACATTTCATCTTGTTATTCGATAGTTCCTACACACTTATTATTTTATTTATTCAAAAAT<br>AAATGTGTTTTGCTTTC
ppl-1	G. multidiverticulata	pyrokinin prohormone like-1 [Schmidtea mediterranea]	3,00E-28	TCTCATGCTCAGAAGCAACAA	CCGATCCATTAATGTTCAGTCT	TTGGATTTGAAGAAATTGAAAGG	CTATATTTCTCCTATTTGTTACAATCATAGGAATATTCTCATGCTCAGAAGCAACAAGTACAACCGATCCATTAATGTTCAGTCTGATTGTACTCATAGCAAACTGTGGTGTA<br>ACACGAACCGAAATGATTCCAAAAAACGATTTAATGATGTTTGATAATTTCATGAATATTCCAGAACAAGAAAAAATGAAAAAATATTTTGCTTCCAAAACCGGGAAACGAT<br>ATTTTTCCCCGCGAATGGGAAAAAGGTACTTTTCACCGAGAATGGGTAAACGTTATTATTCTCCGAGAATGGGTAAACGGTATTATTCTCCGAGAATGGGTAAAAGATATTT<br>TCAAAGAATGGAATAAATGAAACATTTTCTTTTTCATTATGAGAATAAAATATGATTGAAATGTGTTTGAAAAACCAACTTTCATTTACGAATTTTTTTACCTTTCAATTTCTTC<br>AAATCCAAGGTTTATACCGTTCAAATCTCATTTATTTCTAATTTCCGTTAAATTTTTTTAATCCACG
ppl-1	G. dorotocephala	pyrokinin prohormone like-1 [Schmidtea mediterranea]	2,00E-28	GATGCAACAAGTACGGCAGA		TGTTTCATTTATTCCATTCTTTGAA	CAAACAAACTTCAATAAAATTTTATTCTCATAATGAAAAAGAAAATGTTTCATTTATTCCATTCTTTGAAAATAATATCGTTTACCCATTCTTGGTGAAAAGTACCTTTTTCCCA<br>TTCTCGGTGAAAAGTACCTTTTGCCCATTCGTGGTGAAAAGTACCTTTTTCCCATTCTCGGTGAAAAGTACCTTTTGCCCATTCGTGGTGAAAAATATCGTTTTCCGTTTTTGG<br>GATCTACATATTTGTTCATGCTTTCTAGATCCTGAATATTCATGACATTATCGCGCACCATTAAATCGTTTTTCGGAATCATATAAGTTCGTGTTACACCACAGTTTGCTATGA<br>GTACAATCAGACCGAACATTAATGGATCTGCCGTACTTGTTGCATCTGAGCATGAGAATATTCCTATAATTGTAAC

rx3	G. multidiverticulata	retinal homeobox protein Rx [Dugesia japonica]	2,00E-155	TTGCAAATGGTTTGAAAAAGG	AACCGGGCAAGGTTTAGAAT	CGCTATAATATTGAGGATATTCTTGGA	ATTCCTTACAAAGGTTTTGTACATAGATCATAATAATTGCAAATGGTTTGAAAAAGGATTTTTGTTATTCTCCATTAACGTTATCCGATGCTCTAACCGGGCAAGGTTTAGAA<br>TTGGAATTTAAATCTTTTGAAGATCTGGTAGATAGAAAATTAGAGAATTCATTAAATATTCCATAAGGAAGAGTCATTGGTATCTTTGGTGGATCCAAGTTAAGAGATGAGA<br>AATTATTGAATCCCAAATTGTTTACCATTGACATTGGATTCAAGATTCCATTTTGATGATAAAAAAATGATGTCGATTCAACATTCGGTAAAGACATATTTAAAAATGGATAA<br>GGATTAGCGGGAAATAAACTCGAACTGAGAGATTTTGCGACATTTGGGAATACTTCTTGAAGATTATGGGTATGATTAGCGGCTTCTATTTCTCTTGTCGACGCCATTTAG<br>CTCTGCGATTTTGAAACCACACCTGGACTCTGACTTCTGGTAAACTGATCTTCATAGCCAATTCTTCTCTACTGTAAACATCTGGATAATGACTCTTTTCGAACGCTCTCTCCA<br>GTTCGTGAAGTTGATATGTTGTGAAAGTGGTTCTGTTTCTTCTGTGTTTTTTATTACAGTTACTCAATCGGTCTGGAGACGATTCTGTTTCATCAGCTTCACTGATTGCTGGTG<br>ACTGTTCGGAACTGTCTTTTTGACATCTTTTTACGATTAGTAGTTCTCGTTTTGTAATAGGAATTTCTTTACCAGGGTAATCATCATTCATTTCATTTTCAATTGGCTGATTTGG<br>AAAACTATTTAACATAAGAGATTTCTGGTCTTGTAATCCAAGAATATCCTCAATATTATAGCGCTTATTAAT
six	G. multidiverticulata	sine oculis-1/2 [Schmidtea mediterranea]	0	GCAAATTCGACGTTTGGTCT	GTCGTCACATTCGGAAACCT	ATCGATATCCACGAGCCAAG	ATTAAATGAAAAATATTAAAAAATTAATTGTAACTGTAAATAGGCGTATCATAGTCTGGGTTATGGAAATTGCCATAGGTATTCTGAGAGAAATATGGATTGTAGGAACTAC<br>TATTAGTAGAACTGTTTGTATTAGTACGGGAAAGAGGATTTTCCGGAATATTTGTGGTGAAAATTTTATCGTAATTCAGATAAGTGGAATTTGTACTCGACATGGAATGATA<br>ATCACTGTTGGAGGTGTCGTATGCAGGAGGGTTGAGCAAATTCGACGTTTGGTCTGTGGAATCTAAGTCGTCACATTCGGAAACCTGATTGAGCTTTAATAATTTTGTTGAA<br>TGATCATTTTTTCCTGAATTCATATAACTAGTGTCTGTATTAACGAGAGATTCTCTACTAAGAATATTGGGATATTGTCCGGTTATGGGAAGGTTATGTAACCCCGAACTAAA<br>GTAAGAAATATAATTGGCTGCAGGATTGGAATTGTAAATGTACGGAGTACAATAACCTCCTTGAATACCAGTAGCACTTGTATTGAACGGACTCACGGTTTCATTAAATCCC<br>GAAAACATTAAACTTGGATTTCCAACTCCAATTGGGTACGATTGTAGTTTCTTCATCGGTCCTCCATTTTCATGGTTTCCTTCTTTAATATCCTTTAAAGACTCACTGTCGGCAT<br>CAGAAGCGGAGAGATCATCATGTTTGTCTTTATTTTCAGCAGCTCGATCTCTTTGTCTTCGGTTTTTGAACCAATTGGAAACTTGTGTCGTCGTCAAACTCGTCATTTCAGCA<br>AGATCTTTCTTTTCCCGTGGTGACGGATAGGGATTATGCAAATACCATTGTCGAAGAACGGCTCGGGATTTTTCTTTAAAACAATAACTCGTTTCTTCTCCATCCCAAATCGT<br>TCTCGGTAAGGGATATTTTCGTCGAATTCGATATTTCGCGACAGCTCCTAGTGAGCGTCCTTTAATTTTTTCTTCTTCAATATAATGAGCTTGTAACCAGAGCGCTTGAAGTTT<br>ATAATGATTATGCGGTGAGAACGTATAAGATTCTAATATTCGATAAAGCTCTTTGAAATTTTGTCTATGAAATGCGACAGCTGCTTTTGCAGTCAGTACGCTTTCGTTGGTTT<br>GAAGCTGTTGGCAAGGTGGAAGAGACCATATGAATAATGCTAATCTGTCAATATTACCCCCGTTTTCAAGGACCTCACAAACACAAGCAACCTGTTCTTGTGTGAAACCCAT<br>CGTACCGGAATCGGACGAGATCACTGAGTCCTGAGGAGTTATATAATCTAAAGTCGGTGCTGCGAGACATGTCTGTTCATAGGGTACCATCCCGAACTGATGGAATGTAG<br>AATAATTAACATTCGACTCCGGTTCGGACGGAGACGAAACAACCGGGCCATCGAGTGATTTGTAGTCACCTGAATTAGAAAATTGATCACATATATTTTGTTGAATGTCTGT<br>TGCTTGGCTCGTGGATATCGATCTCATAGTCAAACAAGTAATTTGAGATGTCGGTAAAGCAACCTAATATTCTGTTGTTGAATTTATCAATGCAATTTATTTAAACAAATAGA<br>ATTCTATTTTGGTTCAGCAATTAATTCAAGTAGAGTCCGCGTTAAATTTCAGCCAATGAGAAAGCAAGGATTCGTGAAGAGTGGTGTGTGGTGCTTATATTTAGCAAGAACG<br>AGTTTCAAGAGATCTGATTGGTGGAGATTTAAAATAACTCGACCAATTACAATTGTCTTTTGAGAAATTCTTTGAAAAACAATTTTTTGTTTTGAGGAAAAATTTAAATTAAA<br>AAAACAACCTTTTTTTCTTTT
smedwi-1	G. multidiverticulata	DjPiwiA [Dugesia japonica]	0	GTTTATCGACGCGACCAAAT	AAGTTGAAGAAGCGCCAAAA	CGGTTTGACAACCTTTTCGT	ACGAGATTGAACTGGGTGACTGGAGTTCAGACGTGTGCTCTTCCGATCTAAATAATTCATTTGTTTAAATAGATCGTTAGATAATATCGTATTTCTTTTCAAACGTTCTTTGTA<br>TGGATCCAGCAAACACAGCAACAACAAGACCAAGACGAGGGGGACTCCAACGACGATTTGCAATCATCGAACCAAGTCTTCAACCTGACACGGTTGTCGATAAAATCGGA<br>CGGGATGGACGCGCGGTTAACCTAAAATCCAATTACGCCAATTTTAATATTAAAACCGACGGCCCGTTTTTTATGTACGACGTTGTTTCTGAGAATAAAAGTATAAGATCTA<br>AGTTAAATTTTCTTACGACTTACGCTAATACTAAAAAAAAATAAGACGCCATTTTGTTTTGACGGTCGACGATTATTTACCGTTGTCGAGTGGCACGGTGACGATACCACGGA<br>AACGATAAAATTAAACGACACTAAGTTATCGATCCGATATTTAACGAGCTTCATAAAGAACAGCGAAGAATATTACCAAATGATCAATATTGTGTTCAATAATATTCAAATTT<br>TTATGGGACAAGAGAAAATCGGTCGACAATATTTCCTCTCATCGAACTGTGAACAAGTGTCAATTAAAGAGGGACCGAGTTTTTTCAAGATGGAGAATTTCAAAATCCTTG<br>GAGGATATTCGACGACAATCACCCGAGGAACGAATGAAGACGGATCGGCGTCGACTTTGTTGTATTTGGAGAGAATAAATCGTGTGTTGAATGAGAGTTCTGTTTGTAGT<br>GTTTATCGACGCGACCAAATCGATCAATTGATCGGTCGAGATATTATTACAAAATATAACAATAAAACGTATCGAATTTCTGATATTAAAGAAATGGGTATTGATGATGAGA<br>TTGAGTTAAATGGAAAGAAAATTTCCTATAGGGAATATTTTAAACAGCGATATAATATAAGTTTAAAAACCGATAAACAACCGTTCGTTATTTCTCGATTAAAACGATCGAT<br>CGCTCCGAATCTAAGAAATCGAAACAAAGCCGAAGGCGGGGGGGAAGTTGAAGAAGCGCCAAAAGAACCTGATCAAAGTTTATCAATCCCCGGGGAATTGTGTTATTTGT<br>GTGGATTTAGCGATAGTGAACGATCGAATATTAATTTACAGAAAAGTATGGGTGGGGTTTTGAAACGAGAACCGCGGGAACGTTTAAACGACATTAAGGATTTTTGTAAAT<br>GGACAAATAATGGGGCGCAAACGAAAGAATTTATGAATAGTTGGGGAATGGCAATCGATGAGCAACCGATTACGATTAGAGGGAGAGAAATGCCACCGGTTGATATTTT<br>CTCCATGGATAATAAAATCAACGCGAAAATACCCGACGATTGGAAATTTGGGGAGGTTAAATTCGATATTCCCAAGGGCACAAGTCATCGATTTGCGGTGTTGGTTGTCGA<br>CAGAAACCCGGCTCATTTCAACAGTTTCATTGAGGATATGAAACGAGAAATCGGTCGATTAAGAATCAATTATTCAATCGAGGCGGTTTCATCGTGTCGCGGAAACGAGGT<br>CGAAGATGCGTTGAGTGAATTTATTAAAATCAATAAAGTGCATATGGCTCTGGTTTTTATTCCTGACGATAAAATTTATGCCAAAGTTAAAAATTTTACAATGTCAACGGGAT<br>TATTGACGCAATGCGTTACGCAACGTAATGGAAGTAATCGAGACGATCGACGAAGGAAAGTTGTGGCCGATAAATCCGTTATGCAAATGTTCTCGAAACTTGGCTATGATC<br>CGTGGGGAATTAATTTGAAAATGGCACCGACGATGATTGTCGGATTGGACACGTTTCATAGTAAAACGGGAAAGCGGTCTGTGCAGGCGTCGGTGTTTTCGATTAGCGCC<br>AAATTCTCCCAGTACATTAGTTTCGTCAACAGTTCCAAGGGGAAAAATGAATTTCACGAGAATCTCGGCAAGAATTTCCTGACGGCGATTAATACATTCCAACAAAAATTCA<br>ATACGATGCCGCAAAGATTCATTATTTACCGAGACGGAGTCGGTGATTCTCAGTTGGCGTTCACGAAGAAATACGAGACGGAGGCCGTTATGAAAATGATTGAGAAAATC<br>TACGAGAATCAATCATTGCCTCAAATATTATACGTTATCGTTAAGAAAAGAATTAGCGTTAAGTTCTTTAAAGATGGCGGAAATCCCAATCCGGGTACGATTGTTGACGAAA<br>AGGTTGTCAAACCGAATTTTTATGAATTTTATCTCGTTTCTCAAAAAACAACGAAAGGAACGGCATCTCCTACTAACTACAATGTATTAATGGACACAAAAATGACAAATAA<br>AAAGACTAATGAGGTAGCAGCGATGGCTCCCAGTGTTTTACAACAAATTACCTATTCTCTGACGCACCTCTATTTCAACTGGATGGGCACCATTCGGGTGCCTGTGCCGACC<br>CACTATGCCCACCGACTGGCAGAACTCGTTGGGAAAGTCCATCGGGGTGCCAATCCTCCAACTATTAACGACAGGATTAGAGAACGGCTTTTTTACCTTTAGACGTCTGTTT<br>ATACTGCTGTAACTTTGCATTTTCGTCATTGTTCTTGATGTTATAATAAATTTTAGACTTTCATAATTCTATTGATTCGTATTTTTGAAATTTAAATTTAAAAATTG
tyrosinase	G. dorotocephala	tyrosinase [Schmidtea mediterranea]	3,00E-169	CGTCAGATGTTTGTGGAGGA	TAAATCCGGATGGTCTGGAG	CATTACCAGCATGACCGATG	[illegible]<br>TAATTCCAATACGCTGCATCAATAATGCCACTCTACCAAATGCCACTTGTTGTCCTTTTATTCAAAATTCGTCAGATGTTTGTGGAGGAAATGAACGAGGTAGTTGTGAAGCC<br>ATAACTTTGCACCGAGACCATAAAGAAGGAATAGAAAATAATAAAGAACATTTAAAAAATGATGATCGAATAAATTGGCCATTGAAATTCTTTCAATTTGTTTGTAAGTGCA<br>AGAAGAATTTCGATGGGGTTGCATGTGAGTCATGTAAATCCGGATGGTCTGGAGGAGATTGTAAGATTGCTGTTGAAAAAAAGAAAAGACAGAATGTACAAATGTTGTCC<br>GATTTCGATAAATTTGTGTTTCAAACAGTCCTTAATATAAGTAAATATAAGAGCTCTGAATATGCGATAATCAATTCTTCTTCCAACGCTCATTGTGATCCGATTTTCCCGAGT<br>ACAATTCAGTTCATAAATGTCTCAATATATGATACAATGGTCTATCTTCACTTTTATGCGTCTAGGAGCACTATGTCAAGGGAATCGATGACATCTTGCACGGGGGACGATA<br>ATGCATTGGATTTTGCACATAAAGGTCCAGCTTTTTTTACGTGGCACCGTCAATATTTACTATTATTTGAAGAATTATTGGGAAATATTGCGATTGAACAATTTAGTATTTATA<br>ACTTTAGTTTACCCTATTGGGATTGGACCAATGACACTATTTGTTCAGTCTGCGTTGAAGATTTAGTGGGAGTTATACCTTCCGACCAGGAACAGTCAAATAGCAGTTTTTAT<br>CAATGGAGACCAATATGTCCTCAAAATCCTTCTGATTCTGCTTGTCATATATGTGATCCTAATCAAGAGCAACCTTTATTATGGAGAGAATATTCATCAACGGTTGATAGATT<br>ACCAAAACAAGATGAAATTGATGAATTATTTACCATAAATAATTATGATTCGGATCCTTTTAATGCTAATACATTCAGTCGGCAAAGTTTTCGAAATTTCGCAGAAGGCTTTC<br>TTCGTTTGGATCCAAATAAAAATGATTTAAGAATGAATGAAATTGGAAAATCCGTTAAAAATCACAACCTATTATTACACAACATGGTGCACAGTTTCATTAATGGGACATG<br>GATGGATCTTACAACAAGCCCTAATGACCCCCTGTTTTTGTTACATCACACGTTCATGGATAAAATTTTCGAGATGTGGCTTCTCAAAAATTCGAAAAGTAGATTTCCGACAA<br>ACATAACAGTTCCCATCGGTCATGCTGGTAATGATTACATGGTCCCGTTTATCCCACTTAGGAAAAATAAGATGTTGTTCAAGAGGTCATCGGAATTTGGATACGAATATGA<br>CAACAACTACATCGGATCAAGACTCTTTGTAGAACCTTCTTCAGAGGAAACCAGTGACTTCAACTTGATATCGTACATCATAATCGGAGTTATCGTACTTGGGATAGTAATC<br>GGCATAGCTGTATCCTGTTATTATATTCACAGAAACAGAATTAATCGAAATTACCAACCTCTCGATGATCCGGTTATTTTGACGAGGAAACCTTTGTATTCCTACAATCCGGA<br>AAAAATACCTTTACTGAAATAATTACTGAAGCTGGATATACATTTTTATAATTATTTCTCATATGATTTAACGAACTTTGATTAAAT
agat-3	G. dorotocephala	glycine amidinotransferase [Dugesia japonica]	4,00E-141	GCCATTCCACTGCTAACCAT	CCCGTCAAAAGAAAAGTCCA	TTCAAGCGACACTCATCCAC	TTTATGGTCTAATCATCATATTGAACCTTGGTTTGAAGCGTGTGGGTACCCACATATTTTCACATTATTCAAACTCAGCAAATGAAGATCAAATACATAATTTGATAATTGAC<br>TACTACACATACTTACGAAATATAATATAATAAAATGTCACAATTTCATACCTTCTCTAACTCATTACAAGTTATTTGGTCTGAAACAAAATGAAAACAGCAAAAGTCTTAATT<br>TTTAATTCATTGAAACTGTCCAAAGCAATCCCAAGAAGCGGTCGGTTGAGTCGTCAATTATCGTGCCATTCCACTGCTAACCATTTAACTAGTCCTGAGGCTCTAGCGGCCC<br>GTCAAAAGAAAAGTCCAGTGTGGTCATGGAACGAATGGGATCCCCTTGAAGAATGCATGGTTGGTATTCCTGACAATGCTGTTGTTCCGACTATGACCCCGGAAGTTAAAG<br>CTTGTATGAGTCCAGATCTGTGGGACTTTCTGGACAAAAATGGTGGAAAATATTGGTCTGATGTTCTACCAGAAGGTCACTGGGAAAAATTGAAACTGGAAATCACAGAAT<br>TTGCCAAGGTTCTTGAAGGTGAAGGTGTTAAGGTTGTTAGACCAGAAGCTCAAGATTTCGGAAATATTTTTACAGTTCCAGGATTTTCATCCCATGGTATATATGCTGCCAT<br>GCCCAGAGATTTGGTTCTGGTGGTGGGAGATGAACTAATTGAATCCACTATGACCTGGAAAAGTCGTTACTTTGAATTCGTTTCTTATCGAAAGCTGCTGAAAGATTATTAT<br>GACCGGGGCGCAAAATGGACAGTAGCCCCAAAGCCTTTAATGTTAGATGAGTTGATAGAAAAAAATTACGATTTTTATGGAAAAAATAAATTAAAAAGAGTTACAACGAAT<br>AAAGAAATATGTTTTGATGCTGCAGATTTTTTCCGGTGTGGAAGAGATATATTTGGTCAACGAAGTCATGTGACCAATTCAGCAGCCATCAATTGGCTTCGAAGACATCTTG<br>CACCTCAAGGGATACGAGTACATGAATTAAACTTTTATGATTCTTTTTGTATGCACATTGATGCGACTTTAGTTCCAATAAGACCTGGGGTTTTCCTGAAAAATCCTAAATAC<br>CCTTTGGTAGAAATGGATATGTTTACAAATTCTGGCTGGGATTTTGTAGATGTACCAACACCACTTTTAAGCGAAGAAACCTTATACATGAGTTCAAATTATTTGGCCATGAA<br>TACATTAATGATGGATTCCAAGCGCGTATTTGTAGAAAAAGAGGAGACGGGAACTCAGAAATTGTTTGAGTCATTAGGTGTGAAGAGTATTCCTGTAAAGATGAAGCATTG<br>TTTCTCTATCGGAGGAGGATTCCACTGTTGGACTTGCGACGTGAAGAGAAAAGGCACTTTGGAAAACTATTTCAACTGGTCCAACGCAGTCAAAGATAAATACAGTGAGCA<br>ATACGAGCAATAAATGATTTGATATTCCTTGAAATTGAATTTCGTTACTTCGTTTTAGTATAATTAATTGTTATGTTATAAGTTTTGCCTAAAACATTAAATAAATTGTGTTTCG<br>ACATAATAACGATTGTGTATTATATAAGTAATCGATTTTTTGTTCTTATTGTTTAAATAGTTTTAATAAAGTGGATGAGTGTCGCTTGAACATTAATTTTATCATTGTGTGATC<br>TAATTAATACCACTTTGTTTGATATTGTTAAATTTTGATAAATAATATCTGTTTGTTGTTTGAAAAAAGAATTAAGAATGAAATTGTTATAATTATATGGAATTTGG

Table S5 - Probes used for In situ Hybridization Chain Reaction

pair	P1	P2	score	p1score	p2score	gene	pool	initiator	LEFT.SEQUENCE	LEFT. SPACER	RIGHT. SEQUENCE	RIGHT. SPACER	P1_seq	P2_seq
gd_3246_0_2_2_pair1	AGGAAGTTCTTGGCCAGGTTCTCGT	TTGCTCTGGAACGTGTTGATTGCCG	24,32	14,8	9,51	gd_3246_0_2_2	LS_HCR_gd	B2	CCTCGTAAATCCTCATCA	AA	ATCATCCAGTAAACCGCC	AA	CCTCGTAAATCCTCATCAAAAGGAAGTTCTTGGCCAGGTTCTCGT	TTGCTCTGGAACGTGTTGATTGCCGAAATCATCCAGTAAACCGCC
gd_3246_0_2_2_pair2	CGCCGTTCCCTTGGTAGTTTTCTGG	CATGAGCACGTTGTAGTTGGTCGGT	25,56	13,68	11,88	gd_3246_0_2_2	LS_HCR_gd	B2	CCTCGTAAATCCTCATCA	AA	ATCATCCAGTAAACCGCC	AA	CCTCGTAAATCCTCATCAAACGCCGTTCCCTTGGTAGTTTTCTGG	CATGAGCACGTTGTAGTTGGTCGGTAAATCATCCAGTAAACCGCC
gd_3246_0_2_2_pair3	TAATCGAGAACACGGACGCCTGCAC	AGCTGATGTACTGGGAGAACTTGGC	25,74	11,5	14,24	gd_3246_0_2_2	LS_HCR_gd	B2	CCTCGTAAATCCTCATCA	AA	ATCATCCAGTAAACCGCC	AA	CCTCGTAAATCCTCATCAAATAATCGAGAACACGGACGCCTGCAC	AGCTGATGTACTGGGAGAACTTGGCAAATCATCCAGTAAACCGCC
gd_3246_0_2_2_pair4	CAAGTAGAACAGGCGCTCGCGGATG	CACACAGAGAAACGACAAAGACGGC	27,56	14,32	13,24	gd_3246_0_2_2	LS_HCR_gd	B2	CCTCGTAAATCCTCATCA	AA	ATCATCCAGTAAACCGCC	AA	CCTCGTAAATCCTCATCAAACAAGTAGAACAGGCGCTCGCGGATG	CACACAGAGAAACGACAAAGACGGCAAATCATCCAGTAAACCGCC
gd_3246_0_2_2_pair5	TCGTCATGAATTCGCGAGTCTGTGC	GCTGCTCGTCAATGTTCATTCCCCA	29,04	16,76	12,29	gd_3246_0_2_2	LS_HCR_gd	B2	CCTCGTAAATCCTCATCA	AA	ATCATCCAGTAAACCGCC	AA	CCTCGTAAATCCTCATCAAATCGTCATGAATTCGCGAGTCTGTGC	GCTGCTCGTCAATGTTCATTCCCCAAAATCATCCAGTAAACCGCC
gd_3246_0_2_2_pair6	GCTGATGCGCTTCTTGACGATCACG	GTTGCCGCCGTCCTTGAAGAACTTG	29,13	14,71	14,43	gd_3246_0_2_2	LS_HCR_gd	B2	CCTCGTAAATCCTCATCA	AA	ATCATCCAGTAAACCGCC	AA	CCTCGTAAATCCTCATCAAAGCTGATGCGCTTCTTGACGATCACG	GTTGCCGCCGTCCTTGAAGAACTTGAAATCATCCAGTAAACCGCC
gd_3246_0_2_2_pair7	GGACTTGTCGGCCACCACCTTTCTG	GCCGAGCTTAGAGAACATCTGCATG	29,82	15,5	14,31	gd_3246_0_2_2	LS_HCR_gd	B2	CCTCGTAAATCCTCATCA	AA	ATCATCCAGTAAACCGCC	AA	CCTCGTAAATCCTCATCAAAGGACTTGTCGGCCACCACCTTTCTG	GCCGAGCTTAGAGAACATCTGCATGAAATCATCCAGTAAACCGCC
gd_3246_0_2_2_pair8	TTTGTCGTCGGGGATGAACACGAGA	GGTGAAGTTCTTGACCTTGGCGTAG	30,47	13,02	17,46	gd_3246_0_2_2	LS_HCR_gd	B2	CCTCGTAAATCCTCATCA	AA	ATCATCCAGTAAACCGCC	AA	CCTCGTAAATCCTCATCAAATTTGTCGTCGGGGATGAACACGAGA	GGTGAAGTTCTTGACCTTGGCGTAGAAATCATCCAGTAAACCGCC
gd_3246_0_2_2_pair9	AATCGATAGACTCTGGTCGGGCTCT	CCCGCACAAATAACACAATTCTCCG	30,8	14,54	16,26	gd_3246_0_2_2	LS_HCR_gd	B2	CCTCGTAAATCCTCATCA	AA	ATCATCCAGTAAACCGCC	AA	CCTCGTAAATCCTCATCAAAAATCGATAGACTCTGGTCGGGCTCT	CCCGCACAAATAACACAATTCTCCGAAATCATCCAGTAAACCGCC
gd_3246_0_2_2_pair10	GCGTGAGCGAGTACGTGATCTGCTG	TCGTGCCCATCCAGTTGAAGTAGAG	31,35	14,68	16,67	gd_3246_0_2_2	LS_HCR_gd	B2	CCTCGTAAATCCTCATCA	AA	ATCATCCAGTAAACCGCC	AA	CCTCGTAAATCCTCATCAAAGCGTGAGCGAGTACGTGATCTGCTG	TCGTGCCCATCCAGTTGAAGTAGAGAAATCATCCAGTAAACCGCC
gd_3246_0_2_2_pair11	AAGTGCCGGTTCGATGATGGCGAAT	GATTTTATCGACCACGGTGTCAGGT	31,88	15,25	16,63	gd_3246_0_2_2	LS_HCR_gd	B2	CCTCGTAAATCCTCATCA	AA	ATCATCCAGTAAACCGCC	AA	CCTCGTAAATCCTCATCAAAAAGTGCCGGTTCGATGATGGCGAAT	GATTTTATCGACCACGGTGTCAGGTAAATCATCCAGTAAACCGCC
gd_3246_0_2_2_pair12	CCGTGCCTTTAGGGATGTCGAATCT	CGACCACGAGCACTCCGAAACGATG	32,43	15,15	17,28	gd_3246_0_2_2	LS_HCR_gd	B2	CCTCGTAAATCCTCATCA	AA	ATCATCCAGTAAACCGCC	AA	CCTCGTAAATCCTCATCAAACCGTGCCTTTAGGGATGTCGAATCT	CGACCACGAGCACTCCGAAACGATGAAATCATCCAGTAAACCGCC
gd_3246_0_2_2_pair13	CGCGGTTCTCTCTTCAAAACGCCTC	AAATCCCGGATGTCGTTGAGTCTCT	33,5	16,26	17,24	gd_3246_0_2_2	LS_HCR_gd	B2	CCTCGTAAATCCTCATCA	AA	ATCATCCAGTAAACCGCC	AA	CCTCGTAAATCCTCATCAAACGCGGTTCTCTCTTCAAAACGCCTC	AAATCCCGGATGTCGTTGAGTCTCTAAATCATCCAGTAAACCGCC
gd_3246_0_2_2_pair14	TTCAAAACACGATTGATCCGCTCCA	CGATACACGCTACACACACTACTCT	33,5	18,14	15,36	gd_3246_0_2_2	LS_HCR_gd	B2	CCTCGTAAATCCTCATCA	AA	ATCATCCAGTAAACCGCC	AA	CCTCGTAAATCCTCATCAAATTCAAAACACGATTGATCCGCTCCA	CGATACACGCTACACACACTACTCTAAATCATCCAGTAAACCGCC
gd_3246_0_2_2_pair15	CTCGAGCATCTTCATGACGGCCTCC	CGGCAGCGGCTGGTTCTCGTAGATC	33,64	13,58	20,06	gd_3246_0_2_2	LS_HCR_gd	B2	CCTCGTAAATCCTCATCA	AA	ATCATCCAGTAAACCGCC	AA	CCTCGTAAATCCTCATCAAACTCGAGCATCTTCATGACGGCCTCC	CGGCAGCGGCTGGTTCTCGTAGATCAAATCATCCAGTAAACCGCC
gd_3246_0_2_2_pair16	AGATATCGACCGGCGGCATTTCTCG	TGGTGTTGATCTTCTGGTCCATCGA	35,41	19,56	15,85	gd_3246_0_2_2	LS_HCR_gd	B2	CCTCGTAAATCCTCATCA	AA	ATCATCCAGTAAACCGCC	AA	CCTCGTAAATCCTCATCAAAAGATATCGACCGGCGGCATTTCTCG	TGGTGTTGATCTTCTGGTCCATCGAAAATCATCCAGTAAACCGCC
gd_3246_0_2_2_pair17	TCAGGAAGCTCGTCAAGTACCGAAT	CCATTTGGTAGTACTCCTCGCTGTT	36,91	16,23	20,68	gd_3246_0_2_2	LS_HCR_gd	B2	CCTCGTAAATCCTCATCA	AA	ATCATCCAGTAAACCGCC	AA	CCTCGTAAATCCTCATCAAATCAGGAAGCTCGTCAAGTACCGAAT	CCATTTGGTAGTACTCCTCGCTGTTAAATCATCCAGTAAACCGCC
gd_3246_0_2_2_pair18	ATGGTTGCTTGTCAGTCTTCAAAGC	TCGATCGTTTCATCCGGGAAATCAC	38,61	17,63	20,99	gd_3246_0_2_2	LS_HCR_gd	B2	CCTCGTAAATCCTCATCA	AA	ATCATCCAGTAAACCGCC	AA	CCTCGTAAATCCTCATCAAAATGGTTGCTTGTCAGTCTTCAAAGC	TCGATCGTTTCATCCGGGAAATCACAAATCATCCAGTAAACCGCC
gd_3246_0_2_2_pair19	CAATCGCCATTTCTTTGATATCGGA	GCTTCCCGCCCAACTCGATTTCATC	39,56	22,3	17,27	gd_3246_0_2_2	LS_HCR_gd	B2	CCTCGTAAATCCTCATCA	AA	ATCATCCAGTAAACCGCC	AA	CCTCGTAAATCCTCATCAAACAATCGCCATTTCTTTGATATCGGA	GCTTCCCGCCCAACTCGATTTCATCAAATCATCCAGTAAACCGCC
gd_3246_0_2_2_pair20	TTCGATGAAATGAAATACTGGCGAC	CCCTCTTTTAACGACACAGAATCGA	40,18	20,55	19,63	gd_3246_0_2_2	LS_HCR_gd	B2	CCTCGTAAATCCTCATCA	AA	ATCATCCAGTAAACCGCC	AA	CCTCGTAAATCCTCATCAAATTCGATGAAATGAAATACTGGCGAC	CCCTCTTTTAACGACACAGAATCGAAAATCATCCAGTAAACCGCC
gd_7196_0_1_1_pair1	AGCGATTCCCGACATACTTGCACTC	ACCTTGTCTCATTCCACCGGCCATT	27,87	16,92	10,95	gd_7196_0_1_1	LS_HCR_gd	B5	CTCACTCCCAATCTCTAT	AA	CTACCCTACAAATCCAAT	AA	CTCACTCCCAATCTCTATAAAGCGATTCCCGACATACTTGCACTC	ACCTTGTCTCATTCCACCGGCCATTAACTACCCTACAAATCCAAT
gd_7196_0_1_1_pair2	CTGCCATCGTTGTTGGTTCTGACGA	AAACGAGAGCGAGTGACGGATGGAA	29,24	15,85	13,39	gd_7196_0_1_1	LS_HCR_gd	B5	CTCACTCCCAATCTCTAT	AA	CTACCCTACAAATCCAAT	AA	CTCACTCCCAATCTCTATAACTGCCATCGTTGTTGGTTCTGACGA	AAACGAGAGCGAGTGACGGATGGAAAACTACCCTACAAATCCAAT
gd_7196_0_1_1_pair3	CTGCCGCTCTTTGACTCTGTCTTCC	TTTCCGTGGCATTGGATCCAGGACC	31,21	13,69	17,52	gd_7196_0_1_1	LS_HCR_gd	B5	CTCACTCCCAATCTCTAT	AA	CTACCCTACAAATCCAAT	AA	CTCACTCCCAATCTCTATAACTGCCGCTCTTTGACTCTGTCTTCC	TTTCCGTGGCATTGGATCCAGGACCAACTACCCTACAAATCCAAT
gd_7196_0_1_1_pair4	TACACGGTATTCATTGCAGTTTCGT	TAAATCGACGGACCGGTTGGCAGAG	32,92	17,68	15,24	gd_7196_0_1_1	LS_HCR_gd	B5	CTCACTCCCAATCTCTAT	AA	CTACCCTACAAATCCAAT	AA	CTCACTCCCAATCTCTATAATACACGGTATTCATTGCAGTTTCGT	TAAATCGACGGACCGGTTGGCAGAGAACTACCCTACAAATCCAAT
gd_7196_0_1_1_pair5	CACTTGTTGTGAATTGTAACCGGCC	TCCCGGCAAGTTTAGAGACAATGTG	33,49	16,95	16,54	gd_7196_0_1_1	LS_HCR_gd	B5	CTCACTCCCAATCTCTAT	AA	CTACCCTACAAATCCAAT	AA	CTCACTCCCAATCTCTATAACACTTGTTGTGAATTGTAACCGGCC	TCCCGGCAAGTTTAGAGACAATGTGAACTACCCTACAAATCCAAT
gd_7196_0_1_1_pair6	CACACCGACTTTTCGAGAGAGAAAT	ATGCGGTCGCAACCATTCACAATCA	34,09	19,49	14,6	gd_7196_0_1_1	LS_HCR_gd	B5	CTCACTCCCAATCTCTAT	AA	CTACCCTACAAATCCAAT	AA	CTCACTCCCAATCTCTATAACACACCGACTTTTCGAGAGAGAAAT	ATGCGGTCGCAACCATTCACAATCAAACTACCCTACAAATCCAAT
gd_7196_0_1_1_pair7	TCCAGGCTTTTCAGGACTTCTACTT	TGGATGAAGCGTCCAGTAAGAACCC	34,88	17,91	16,96	gd_7196_0_1_1	LS_HCR_gd	B5	CTCACTCCCAATCTCTAT	AA	CTACCCTACAAATCCAAT	AA	CTCACTCCCAATCTCTATAATCCAGGCTTTTCAGGACTTCTACTT	TGGATGAAGCGTCCAGTAAGAACCCAACTACCCTACAAATCCAAT
gd_7196_0_1_1_pair8	CTGCGGCGTATAGGCTATCATGGGG	GCTCGAGATCCATTGATGATCCAGC	35,13	16,23	18,9	gd_7196_0_1_1	LS_HCR_gd	B5	CTCACTCCCAATCTCTAT	AA	CTACCCTACAAATCCAAT	AA	CTCACTCCCAATCTCTATAACTGCGGCGTATAGGCTATCATGGGG	GCTCGAGATCCATTGATGATCCAGCAACTACCCTACAAATCCAAT
gd_7196_0_1_1_pair9	GGCGGGATATTTGAATAGTTTGAGT	TCGTAGGCTGTCATTGTCGATGTGG	37,02	24,51	12,51	gd_7196_0_1_1	LS_HCR_gd	B5	CTCACTCCCAATCTCTAT	AA	CTACCCTACAAATCCAAT	AA	CTCACTCCCAATCTCTATAAGGCGGGATATTTGAATAGTTTGAGT	TCGTAGGCTGTCATTGTCGATGTGGAACTACCCTACAAATCCAAT
gd_7196_0_1_1_pair10	TCTGTTATCGGTATTTTGATGGATC	GGGTAGGGAGGCTGTTGGTCTCTAA	40,16	27,58	12,59	gd_7196_0_1_1	LS_HCR_gd	B5	CTCACTCCCAATCTCTAT	AA	CTACCCTACAAATCCAAT	AA	CTCACTCCCAATCTCTATAATCTGTTATCGGTATTTTGATGGATC	GGGTAGGGAGGCTGTTGGTCTCTAAAACTACCCTACAAATCCAAT
gd_7196_0_1_1_pair11	TTTGTCTTGGAATCGCTCTCGGCAT	TCACTGGAAATGTCTAACTGTTTGA	40,36	15,44	24,93	gd_7196_0_1_1	LS_HCR_gd	B5	CTCACTCCCAATCTCTAT	AA	CTACCCTACAAATCCAAT	AA	CTCACTCCCAATCTCTATAATTTGTCTTGGAATCGCTCTCGGCAT	TCACTGGAAATGTCTAACTGTTTGAAACTACCCTACAAATCCAAT
gd_7196_0_1_1_pair12	GAGATCCTGGTAAACTGTCATACGG	GTTGGGAGCCTTCGTATTGATATGT	40,47	17,6	22,87	gd_7196_0_1_1	LS_HCR_gd	B5	CTCACTCCCAATCTCTAT	AA	CTACCCTACAAATCCAAT	AA	CTCACTCCCAATCTCTATAAGAGATCCTGGTAAACTGTCATACGG	GTTGGGAGCCTTCGTATTGATATGTAACTACCCTACAAATCCAAT
gd_7196_0_1_1_pair13	AACTGTAAGGAGGTTTCGCATGGGT	GAATCGCCATTGTTATTAAACTGAT	42,45	14,35	28,1	gd_7196_0_1_1	LS_HCR_gd	B5	CTCACTCCCAATCTCTAT	AA	CTACCCTACAAATCCAAT	AA	CTCACTCCCAATCTCTATAAAACTGTAAGGAGGTTTCGCATGGGT	GAATCGCCATTGTTATTAAACTGATAACTACCCTACAAATCCAAT
gd_7196_0_1_1_pair14	CTGGTTATCAGTCCTCCCCAATTAA	CCAGATTTCTGTATGAGGTGAGCAA	44,07	23,6	20,47	gd_7196_0_1_1	LS_HCR_gd	B5	CTCACTCCCAATCTCTAT	AA	CTACCCTACAAATCCAAT	AA	CTCACTCCCAATCTCTATAACTGGTTATCAGTCCTCCCCAATTAA	CCAGATTTCTGTATGAGGTGAGCAAAACTACCCTACAAATCCAAT
gd_7196_0_1_1_pair15	ACTGGACATTTTCATTTCTACTGGT	TTGTTGTTGAGGAGGTTGTGGAGGT	45,09	25,55	19,54	gd_7196_0_1_1	LS_HCR_gd	B5	CTCACTCCCAATCTCTAT	AA	CTACCCTACAAATCCAAT	AA	CTCACTCCCAATCTCTATAAACTGGACATTTTCATTTCTACTGGT	TTGTTGTTGAGGAGGTTGTGGAGGTAACTACCCTACAAATCCAAT
gd_7196_0_1_1_pair16	CGTCACTTTGTAAATTATGCCCTGA	TGGATGGTAAGGATACGGAACTTTG	45,15	24,61	20,53	gd_7196_0_1_1	LS_HCR_gd	B5	CTCACTCCCAATCTCTAT	AA	CTACCCTACAAATCCAAT	AA	CTCACTCCCAATCTCTATAACGTCACTTTGTAAATTATGCCCTGA	TGGATGGTAAGGATACGGAACTTTGAACTACCCTACAAATCCAAT
gd_7196_0_1_1_pair17	AATTTTGCTGACTTTATCTGAATTC	TCGCGTGTTGATTTGAGTGTTCGGT	45,63	33,48	12,15	gd_7196_0_1_1	LS_HCR_gd	B5	CTCACTCCCAATCTCTAT	AA	CTACCCTACAAATCCAAT	AA	CTCACTCCCAATCTCTATAAAATTTTGCTGACTTTATCTGAATTC	TCGCGTGTTGATTTGAGTGTTCGGTAACTACCCTACAAATCCAAT
gd_7196_0_1_1_pair18	ACTGTTACTTGCTAGGGCACTCGGT	CTTTTACTGAATCTAAAGGCTCATA	45,76	15,46	30,31	gd_7196_0_1_1	LS_HCR_gd	B5	CTCACTCCCAATCTCTAT	AA	CTACCCTACAAATCCAAT	AA	CTCACTCCCAATCTCTATAAACTGTTACTTGCTAGGGCACTCGGT	CTTTTACTGAATCTAAAGGCTCATAAACTACCCTACAAATCCAAT
gd_7196_0_1_1_pair19	GATCGATTGAAAATGGATTACTGGT	CGGCTGCTAAACTAGGATGCATCAA	47,71	28,26	19,45	gd_7196_0_1_1	LS_HCR_gd	B5	CTCACTCCCAATCTCTAT	AA	CTACCCTACAAATCCAAT	AA	CTCACTCCCAATCTCTATAAGATCGATTGAAAATGGATTACTGGT	CGGCTGCTAAACTAGGATGCATCAAAACTACCCTACAAATCCAAT
gd_7196_0_1_1_pair20	TGAGAGACGAATTGAACCGGGTCGG	TGATCGAATATGGAATGTGAATTGT	48,14	16,03	32,11	gd_7196_0_1_1	LS_HCR_gd	B5	CTCACTCCCAATCTCTAT	AA	CTACCCTACAAATCCAAT	AA	CTCACTCCCAATCTCTATAATGAGAGACGAATTGAACCGGGTCGG	TGATCGAATATGGAATGTGAATTGTAACTACCCTACAAATCCAAT
gir_dor_ovo_pair1	CTTGTAAAGTGAGAAAATTTGACCC	AAGAGTTAATAAGAAAAGTGCTCAT	62,56	26,93	35,63	gir_dor_ovo	LS_HCR_gd	B4	CCTCAACCTACCTCCAAC	AA	TCTCACCATATTCGCTTC	AT	CCTCAACCTACCTCCAACAACTTGTAAAGTGAGAAAATTTGACCC	AAGAGTTAATAAGAAAAGTGCTCATATTCTCACCATATTCGCTTC
gir_dor_ovo_pair2	GATAAATCTGTTGGCTGCTCCTCAT	GCGTTTTCGAATCTCCCAGTTTTCA	42,56	22,84	19,72	gir_dor_ovo	LS_HCR_gd	B4	CCTCAACCTACCTCCAAC	AA	TCTCACCATATTCGCTTC	AT	CCTCAACCTACCTCCAACAAGATAAATCTGTTGGCTGCTCCTCAT	GCGTTTTCGAATCTCCCAGTTTTCAATTCTCACCATATTCGCTTC
gir_dor_ovo_pair3	GAACATAATCTATCATCCTTGACGG	TGGCCAAGCATTGAAGGATAATTAT	54,17	28,26	25,92	gir_dor_ovo	LS_HCR_gd	B4	CCTCAACCTACCTCCAAC	AA	TCTCACCATATTCGCTTC	AT	CCTCAACCTACCTCCAACAAGAACATAATCTATCATCCTTGACGG	TGGCCAAGCATTGAAGGATAATTATATTCTCACCATATTCGCTTC
gir_dor_ovo_pair4	TCGCAGTATTAATGAATGGACATCT	AAACGAATACTGATTAAGAAACTGT	52,7	23,42	29,28	gir_dor_ovo	LS_HCR_gd	B4	CCTCAACCTACCTCCAAC	AA	TCTCACCATATTCGCTTC	AT	CCTCAACCTACCTCCAACAATCGCAGTATTAATGAATGGACATCT	AAACGAATACTGATTAAGAAACTGTATTCTCACCATATTCGCTTC
gir_dor_ovo_pair5	AGAAATCATTGAACATTCATTATTC	AAGTATGGGGTAAAACATTGCCGAA	53,23	34,43	18,8	gir_dor_ovo	LS_HCR_gd	B4	CCTCAACCTACCTCCAAC	AA	TCTCACCATATTCGCTTC	AT	CCTCAACCTACCTCCAACAAAGAAATCATTGAACATTCATTATTC	AAGTATGGGGTAAAACATTGCCGAAATTCTCACCATATTCGCTTC
gir_dor_ovo_pair6	CGATATTTAACGAATTCTTTTGGAA	TGCTGGAGCTCGTCGATACATTTGA	47,7	33,76	13,94	gir_dor_ovo	LS_HCR_gd	B4	CCTCAACCTACCTCCAAC	AA	TCTCACCATATTCGCTTC	AT	CCTCAACCTACCTCCAACAACGATATTTAACGAATTCTTTTGGAA	TGCTGGAGCTCGTCGATACATTTGAATTCTCACCATATTCGCTTC
gir_dor_ovo_pair7	CAAGTTCTTCTGAAGAAGACGTTCC	CTTTAATATTCTCAAAGTGAGTGGT	56,82	23,1	33,72	gir_dor_ovo	LS_HCR_gd	B4	CCTCAACCTACCTCCAAC	AA	TCTCACCATATTCGCTTC	AT	CCTCAACCTACCTCCAACAACAAGTTCTTCTGAAGAAGACGTTCC	CTTTAATATTCTCAAAGTGAGTGGTATTCTCACCATATTCGCTTC
gir_dor_ovo_pair8	ATAAATTATCACTAAGATGAAAGTA	TCTCTTCAATGCTCTCTTCAGATTG	60,83	38,42	22,41	gir_dor_ovo	LS_HCR_gd	B4	CCTCAACCTACCTCCAAC	AA	TCTCACCATATTCGCTTC	AT	CCTCAACCTACCTCCAACAAATAAATTATCACTAAGATGAAAGTA	TCTCTTCAATGCTCTCTTCAGATTGATTCTCACCATATTCGCTTC
gir_dor_ovo_pair9	AATGTGTTCAGCAAAATTTCTTCGT	TCCCGGATCTGTAAGATATAATTTT	62,1	29,88	32,22	gir_dor_ovo	LS_HCR_gd	B4	CCTCAACCTACCTCCAAC	AA	TCTCACCATATTCGCTTC	AT	CCTCAACCTACCTCCAACAAAATGTGTTCAGCAAAATTTCTTCGT	TCCCGGATCTGTAAGATATAATTTTATTCTCACCATATTCGCTTC
gir_dor_ovo_pair10	CCGTCCCCACCGTTTGTCAGTTTAA	AACTGTTTCCTGAAAGGGTTCACAA	42,15	20,86	21,3	gir_dor_ovo	LS_HCR_gd	B4	CCTCAACCTACCTCCAAC	AA	TCTCACCATATTCGCTTC	AT	CCTCAACCTACCTCCAACAACCGTCCCCACCGTTTGTCAGTTTAA	AACTGTTTCCTGAAAGGGTTCACAAATTCTCACCATATTCGCTTC
gir_dor_ovo_pair11	TTTTCCCTAATTCTTTACAAAACTT	TTTTGTTGCAGATCTTACATTGAAA	62,12	34,54	27,58	gir_dor_ovo	LS_HCR_gd	B4	CCTCAACCTACCTCCAAC	AA	TCTCACCATATTCGCTTC	AT	CCTCAACCTACCTCCAACAATTTTCCCTAATTCTTTACAAAACTT	TTTTGTTGCAGATCTTACATTGAAAATTCTCACCATATTCGCTTC
gir_dor_ovo_pair12	CGAATATGACGCTGCATATGCTTCT	AGCCAGTCGAAATTAACCCCATGTG	42,43	24,26	18,17	gir_dor_ovo	LS_HCR_gd	B4	CCTCAACCTACCTCCAAC	AA	TCTCACCATATTCGCTTC	AT	CCTCAACCTACCTCCAACAACGAATATGACGCTGCATATGCTTCT	AGCCAGTCGAAATTAACCCCATGTGATTCTCACCATATTCGCTTC
gir_dor_ovo_pair13	AGTCATAAGAATCAATGAAGGGTTT	CTGTGTGGACCCTTGTGTGCCTTTT	43,52	26,7	16,82	gir_dor_ovo	LS_HCR_gd	B4	CCTCAACCTACCTCCAAC	AA	TCTCACCATATTCGCTTC	AT	CCTCAACCTACCTCCAACAAAGTCATAAGAATCAATGAAGGGTTT	CTGTGTGGACCCTTGTGTGCCTTTTATTCTCACCATATTCGCTTC
gir_dor_ovo_pair14	TTTCTGGTGCAACTTTGACATTTGT	CTTTCTAACGAGCAACGCTGACTAA	41,63	22,26	19,37	gir_dor_ovo	LS_HCR_gd	B4	CCTCAACCTACCTCCAAC	AA	TCTCACCATATTCGCTTC	AT	CCTCAACCTACCTCCAACAATTTCTGGTGCAACTTTGACATTTGT	CTTTCTAACGAGCAACGCTGACTAAATTCTCACCATATTCGCTTC
gir_dor_ovo_pair15	TCGATCCCATGAATCTTCACTTGAT	CGGCGCTCCTTATACAAATAATTCA	46,27	21,53	24,74	gir_dor_ovo	LS_HCR_gd	B4	CCTCAACCTACCTCCAAC	AA	TCTCACCATATTCGCTTC	AT	CCTCAACCTACCTCCAACAATCGATCCCATGAATCTTCACTTGAT	CGGCGCTCCTTATACAAATAATTCAATTCTCACCATATTCGCTTC

gir_dor_ovo_pair16	ACTATAACTGCAAATCTCACAAGGA	GTTTAGCCATGTAGTTTTACAGGAA	48,36	22,67	25,69	gir_dor_ovo	LS_HCR_gd	B4	CCTCAACCTACCTCCAAC	AA	TCTCACCATATTCGCTTC	AT	CCTCAACCTACCTCCAACAAACTATAACTGCAAATCTCACAAGGA	GTTTAGCCATGTAGTTTTACAGGAAATTCTCACCATATTCGCTTC
gir_dor_ovo_pair17	AAGGAATTCGGATGCTCTTGAACAA	CCTTTTATAACTTCTTCTTTGGCAT	47,21	19,67	27,54	gir_dor_ovo	LS_HCR_gd	B4	CCTCAACCTACCTCCAAC	AA	TCTCACCATATTCGCTTC	AT	CCTCAACCTACCTCCAACAAAAGGAATTCGGATGCTCTTGAACAA	CCTTTTATAACTTCTTCTTTGGCATATTCTCACCATATTCGCTTC
gir_dor_ovo_pair18	AATAATCAAAATCTTTTCAATTGTC	GATGATGAAGAATAAAATATACATA	78	39,06	38,95	gir_dor_ovo	LS_HCR_gd	B4	CCTCAACCTACCTCCAAC	AA	TCTCACCATATTCGCTTC	AT	CCTCAACCTACCTCCAACAAAATAATCAAAATCTTTTCAATTGTC	GATGATGAAGAATAAAATATACATAATTCTCACCATATTCGCTTC
gir_dor_ovo_pair19	ACTTTTGACTTTTACAAATGACACG	TGGAAATTTATGATCAAATCAACAT	60,2	27,86	32,34	gir_dor_ovo	LS_HCR_gd	B4	CCTCAACCTACCTCCAAC	AA	TCTCACCATATTCGCTTC	AT	CCTCAACCTACCTCCAACAAACTTTTGACTTTTACAAATGACACG	TGGAAATTTATGATCAAATCAACATATTCTCACCATATTCGCTTC
gir_dor_six-1-2_pair1	GCAAATTCGACGTTTGGTCTGTGGA	TGGAGGTGTCATATGCAGGAGGGTT	31,15	15,71	15,44	gir_dor_six-1-2	LS_HCR_gd	B1	GAGGAGGGCAGCAAACG	AA	GAAGAGTCTTCCTTTACG	TA	GAGGAGGGCAGCAAACGGAAGCAAATTCGACGTTTGGTCTGTG	TGGAGGTGTCATATGCAGGAGGGTTTAGAAGAGTCTTCCTTTACG
gir_dor_six-1-2_pair2	TCTGTTCATAGGGTACCATCCCGAA	AATCTAAGGTTGGTGCGGCGAGACA	32,41	18,72	13,69	gir_dor_six-1-2	LS_HCR_gd	B1	GAGGAGGGCAGCAAACG	AA	GAAGAGTCTTCCTTTACG	TA	GAGGAGGGCAGCAAACGGAATCTGTTCATAGGGTACCATCCCGA	AATCTAAGGTTGGTGCGGCGAGACATAGAAGAGTCTTCCTTTACG
gir_dor_six-1-2_pair3	CGGAATCGGACGAGATCACTGAGTC	CCTGTTCTTGTGTGAAACCCATCGT	32,65	14,92	17,73	gir_dor_six-1-2	LS_HCR_gd	B1	GAGGAGGGCAGCAAACG	AA	GAAGAGTCTTCCTTTACG	TA	GAGGAGGGCAGCAAACGGAACGGAATCGGACGAGATCACTGAG	CCTGTTCTTGTGTGAAACCCATCGTTAGAAGAGTCTTCCTTTACG
gir_dor_six-1-2_pair4	TTGGACACTTGGGTCGTCGTTAAAC	TCTCGTTGTCTTCGGTTCTTGAACC	34,11	15,95	18,16	gir_dor_six-1-2	LS_HCR_gd	B1	GAGGAGGGCAGCAAACG	AA	GAAGAGTCTTCCTTTACG	TA	GAGGAGGGCAGCAAACGGAATTGGACACTTGGGTCGTCGTTAA	TCTCGTTGTCTTCGGTTCTTGAACCTAGAAGAGTCTTCCTTTACG
gir_dor_six-1-2_pair5	GGCCATCGAGTGATTTGAAGTCACC	CCGGTTCGGACGGAGACGAAACAAC	34,56	15,4	19,16	gir_dor_six-1-2	LS_HCR_gd	B1	GAGGAGGGCAGCAAACG	AA	GAAGAGTCTTCCTTTACG	TA	GAGGAGGGCAGCAAACGGAAGGCCATCGAGTGATTTGAAGTCA	CCGGTTCGGACGGAGACGAAACAACTAGAAGAGTCTTCCTTTACG
gir_dor_six-1-2_pair6	TCGATCTCATAGTCAAACAAGTAAT	GAATGTCTGTTGCTTGGCTCGTGGA	35,17	25,03	10,13	gir_dor_six-1-2	LS_HCR_gd	B1	GAGGAGGGCAGCAAACG	AA	GAAGAGTCTTCCTTTACG	TA	GAGGAGGGCAGCAAACGGAATCGATCTCATAGTCAAACAAGTA	GAATGTCTGTTGCTTGGCTCGTGGATAGAAGAGTCTTCCTTTACG
gir_dor_six-1-2_pair7	ATGTTTGTCTTTATTTTCAGCGGCT	GTCGGCATCAGAAGCGGAAAGATCA	35,59	21,64	13,95	gir_dor_six-1-2	LS_HCR_gd	B1	GAGGAGGGCAGCAAACG	AA	GAAGAGTCTTCCTTTACG	TA	GAGGAGGGCAGCAAACGGAAATGTTTGTCTTTATTTTCAGCGGC	GTCGGCATCAGAAGCGGAAAGATCATAGAAGAGTCTTCCTTTACG
gir_dor_six-1-2_pair8	CATTGTCGAAGAACGGCTCGGGATT	GGTGACGGATAGGGATTATGCAAAT	36,79	15,38	21,41	gir_dor_six-1-2	LS_HCR_gd	B1	GAGGAGGGCAGCAAACG	AA	GAAGAGTCTTCCTTTACG	TA	GAGGAGGGCAGCAAACGGAACATTGTCGAAGAACGGCTCGGGA	GGTGACGGATAGGGATTATGCAAATTAGAAGAGTCTTCCTTTACG
gir_dor_six-1-2_pair9	TGTATTGAAGGGACTCACGGTTTCA	ATAACCTCCTTGAATACCAGTGGCA	39,61	19,93	19,68	gir_dor_six-1-2	LS_HCR_gd	B1	GAGGAGGGCAGCAAACG	AA	GAAGAGTCTTCCTTTACG	TA	GAGGAGGGCAGCAAACGGAATGTATTGAAGGGACTCACGGTTT	ATAACCTCCTTGAATACCAGTGGCATAGAAGAGTCTTCCTTTACG
gir_dor_six-1-2_pair10	CCTCCGTTTTCATGATTGCCTTCCT	GGGTACGATTGTAGTTTCTTCAACG	40,44	20,17	20,27	gir_dor_six-1-2	LS_HCR_gd	B1	GAGGAGGGCAGCAAACG	AA	GAAGAGTCTTCCTTTACG	TA	GAGGAGGGCAGCAAACGGAACCTCCGTTTTCATGATTGCCTTCC	GGGTACGATTGTAGTTTCTTCAACGTAGAAGAGTCTTCCTTTACG
gir_dor_six-1-2_pair11	ATTGCCATAGGTATTCTGAGAGAAA	AGGCGTATCATAGTCTGGGTTATGG	41,44	23,87	17,57	gir_dor_six-1-2	LS_HCR_gd	B1	GAGGAGGGCAGCAAACG	AA	GAAGAGTCTTCCTTTACG	TA	GAGGAGGGCAGCAAACGGAAATTGCCATAGGTATTCTGAGAGA	AGGCGTATCATAGTCTGGGTTATGGTAGAAGAGTCTTCCTTTACG
gir_dor_six-1-2_pair12	AATGCTAATCTGTCAATATTACCCC	TGGCAAGGTGGAAGAGACCATATGA	42,5	25,91	16,59	gir_dor_six-1-2	LS_HCR_gd	B1	GAGGAGGGCAGCAAACG	AA	GAAGAGTCTTCCTTTACG	TA	GAGGAGGGCAGCAAACGGAAAATGCTAATCTGTCAATATTACCC	TGGCAAGGTGGAAGAGACCATATGATAGAAGAGTCTTCCTTTACG
gir_dor_six-1-2_pair13	AGTAAGAAATATAATTGGCTGCGGG	TGGGAAGGTTATGTAACCCGGAACT	42,59	21,78	20,81	gir_dor_six-1-2	LS_HCR_gd	B1	GAGGAGGGCAGCAAACG	AA	GAAGAGTCTTCCTTTACG	TA	GAGGAGGGCAGCAAACGGAAAGTAAGAAATATAATTGGCTGCG	TGGGAAGGTTATGTAACCCGGAACTTAGAAGAGTCTTCCTTTACG
gir_dor_six-1-2_pair14	TGTCTATGAAACGCGACAGCTGCTT	AAGATTCGATAAAGCTCTTTGAAAT	43,18	13,89	29,28	gir_dor_six-1-2	LS_HCR_gd	B1	GAGGAGGGCAGCAAACG	AA	GAAGAGTCTTCCTTTACG	TA	GAGGAGGGCAGCAAACGGAATGTCTATGAAACGCGACAGCTGC	AAGATTCGATAAAGCTCTTTGAAATTAGAAGAGTCTTCCTTTACG
gir_dor_six-1-2_pair15	CTCGGTAAAGGATATTTTCGGCGAA	CTCGTTTCTTCACCATCCCAAATCG	43,83	25,57	18,27	gir_dor_six-1-2	LS_HCR_gd	B1	GAGGAGGGCAGCAAACG	AA	GAAGAGTCTTCCTTTACG	TA	GAGGAGGGCAGCAAACGGAACTCGGTAAAGGATATTTTCGGCG	CTCGTTTCTTCACCATCCCAAATCGTAGAAGAGTCTTCCTTTACG
gir_dor_six-1-2_pair16	TATTGGTATTCGTACGGGAAAGAGG	GGTTGTAGGAGCTGCTATTAGTTGA	45,35	24,69	20,66	gir_dor_six-1-2	LS_HCR_gd	B1	GAGGAGGGCAGCAAACG	AA	GAAGAGTCTTCCTTTACG	TA	GAGGAGGGCAGCAAACGGAATATTGGTATTCGTACGGGAAAGA	GGTTGTAGGAGCTGCTATTAGTTGATAGAAGAGTCTTCCTTTACG
gir_dor_six-1-2_pair17	AAGTTTATAGTGATTATGCGGTGAG	ATAATGAGCTTGTAACCAGAGTGCT	48,27	26,6	21,67	gir_dor_six-1-2	LS_HCR_gd	B1	GAGGAGGGCAGCAAACG	AA	GAAGAGTCTTCCTTTACG	TA	GAGGAGGGCAGCAAACGGAAAAGTTTATAGTGATTATGCGGTG	ATAATGAGCTTGTAACCAGAGTGCTTAGAAGAGTCTTCCTTTACG
gir_dor_six-1-2_pair18	GCTAAGAATATTGGGATATTGTCCG	AGTATCTGTATTAACGAGAGATTCT	60,05	29,56	30,48	gir_dor_six-1-2	LS_HCR_gd	B1	GAGGAGGGCAGCAAACG	AA	GAAGAGTCTTCCTTTACG	TA	GAGGAGGGCAGCAAACGGAAGCTAAGAATATTGGGATATTGTC	AGTATCTGTATTAACGAGAGATTCTTAGAAGAGTCTTCCTTTACG
gir_dor_six-1-2_pair19	GTACTCGACATGGAATGATAATCAC	TTATCATAATTCAGATAAGTGGAAT	60,16	23,47	36,7	gir_dor_six-1-2	LS_HCR_gd	B1	GAGGAGGGCAGCAAACG	AA	GAAGAGTCTTCCTTTACG	TA	GAGGAGGGCAGCAAACGGAAGTACTCGACATGGAATGATAATC	TTATCATAATTCAGATAAGTGGAATTAGAAGAGTCTTCCTTTACG
gir_dor_sp6-9_pair1	AGAACAACTTATGGAAACTTATGTT	TGCAAATTGACCAATGAGAACAACA	50,14	30,56	19,58	gir_dor_sp6-9	LS_HCR_gd	B3	GTCCCTGCCTCTATATCT	TT	CCACTCAACTTTAACCCG	TT	GTCCCTGCCTCTATATCTTTAGAACAACTTATGGAAACTTATGTT	TGCAAATTGACCAATGAGAACAACATTCCACTCAACTTTAACCCG
gir_dor_sp6-9_pair2	CATTTGGTAACCAATGATATTCATT	ATTCGTTTGATCAATGTATTAAATT	70,09	32,69	37,4	gir_dor_sp6-9	LS_HCR_gd	B3	GTCCCTGCCTCTATATCT	TT	CCACTCAACTTTAACCCG	TT	GTCCCTGCCTCTATATCTTTCATTTGGTAACCAATGATATTCATT	ATTCGTTTGATCAATGTATTAAATTTTCCACTCAACTTTAACCCG
gir_dor_sp6-9_pair3	AGTCCATATTTGATTGATTACGAAA	TGTTCTCGACATTGTTTAGAGAAGT	53,93	31,43	22,5	gir_dor_sp6-9	LS_HCR_gd	B3	GTCCCTGCCTCTATATCT	TT	CCACTCAACTTTAACCCG	TT	GTCCCTGCCTCTATATCTTTAGTCCATATTTGATTGATTACGAAA	TGTTCTCGACATTGTTTAGAGAAGTTTCCACTCAACTTTAACCCG
gir_dor_sp6-9_pair4	TAGCTTTCCATCAACAGTGACTGAT	ACATCATTTATTTTCCTGACAGACT	47,95	22,28	25,67	gir_dor_sp6-9	LS_HCR_gd	B3	GTCCCTGCCTCTATATCT	TT	CCACTCAACTTTAACCCG	TT	GTCCCTGCCTCTATATCTTTTAGCTTTCCATCAACAGTGACTGAT	ACATCATTTATTTTCCTGACAGACTTTCCACTCAACTTTAACCCG
gir_dor_sp6-9_pair5	AGCGGAATTGTTAAATTCGGTTTCA	TATATTAAATCTCCATAAATTGTGA	65,03	24,92	40,11	gir_dor_sp6-9	LS_HCR_gd	B3	GTCCCTGCCTCTATATCT	TT	CCACTCAACTTTAACCCG	TT	GTCCCTGCCTCTATATCTTTAGCGGAATTGTTAAATTCGGTTTCA	TATATTAAATCTCCATAAATTGTGATTCCACTCAACTTTAACCCG
gir_dor_sp6-9_pair6	CTGAACTTTGAGGAACTTGGTTGGA	GCGGATGAAACAAATTATCATTCAT	50,86	21,13	29,73	gir_dor_sp6-9	LS_HCR_gd	B3	GTCCCTGCCTCTATATCT	TT	CCACTCAACTTTAACCCG	TT	GTCCCTGCCTCTATATCTTTCTGAACTTTGAGGAACTTGGTTGGA	GCGGATGAAACAAATTATCATTCATTTCCACTCAACTTTAACCCG
gir_dor_sp6-9_pair7	AGTTTTGGTAGTTTGGTATGGAAAG	GAAGTGGGTATTGATTTTGAGTTGA	50,34	25,88	24,45	gir_dor_sp6-9	LS_HCR_gd	B3	GTCCCTGCCTCTATATCT	TT	CCACTCAACTTTAACCCG	TT	GTCCCTGCCTCTATATCTTTAGTTTTGGTAGTTTGGTATGGAAAG	GAAGTGGGTATTGATTTTGAGTTGATTCCACTCAACTTTAACCCG
gir_dor_sp6-9_pair8	GATTCTGGTAAACTCAGTGATATAT	TGAGACTGGGGTAAATTGAAGAGGT	48,77	31,47	17,3	gir_dor_sp6-9	LS_HCR_gd	B3	GTCCCTGCCTCTATATCT	TT	CCACTCAACTTTAACCCG	TT	GTCCCTGCCTCTATATCTTTGATTCTGGTAAACTCAGTGATATAT	TGAGACTGGGGTAAATTGAAGAGGTTTCCACTCAACTTTAACCCG
gir_dor_sp6-9_pair9	TTTTGGAAGAGTTTGAAAATTATAT	TGATGAAATGAAGGGGTTTAAGTAA	65,88	39	26,88	gir_dor_sp6-9	LS_HCR_gd	B3	GTCCCTGCCTCTATATCT	TT	CCACTCAACTTTAACCCG	TT	GTCCCTGCCTCTATATCTTTTTTTTGGAAGAGTTTGAAAATTATAT	TGATGAAATGAAGGGGTTTAAGTAATTCCACTCAACTTTAACCCG
gir_dor_sp6-9_pair10	TGGATATCTTGGAAGAGAACTATTT	ATTTGGACAGGTGCAAGCGGCACGA	50,39	31,17	19,22	gir_dor_sp6-9	LS_HCR_gd	B3	GTCCCTGCCTCTATATCT	TT	CCACTCAACTTTAACCCG	TT	GTCCCTGCCTCTATATCTTTTGGATATCTTGGAAGAGAACTATTT	ATTTGGACAGGTGCAAGCGGCACGATTCCACTCAACTTTAACCCG
gir_dor_sp6-9_pair11	TGACAGAAAGCCAGAAACCTGTTCT	AATGTGGCAATTATGGGTAGAATTC	44,44	18,56	25,88	gir_dor_sp6-9	LS_HCR_gd	B3	GTCCCTGCCTCTATATCT	TT	CCACTCAACTTTAACCCG	TT	GTCCCTGCCTCTATATCTTTTGACAGAAAGCCAGAAACCTGTTCT	AATGTGGCAATTATGGGTAGAATTCTTCCACTCAACTTTAACCCG
gir_dor_sp6-9_pair12	TGGGTTTCGAATATAGTTTTCCACA	GCCATCTTAAATGTGCTTTCAAATG	56,22	25,42	30,81	gir_dor_sp6-9	LS_HCR_gd	B3	GTCCCTGCCTCTATATCT	TT	CCACTCAACTTTAACCCG	TT	GTCCCTGCCTCTATATCTTTTGGGTTTCGAATATAGTTTTCCACA	GCCATCTTAAATGTGCTTTCAAATGTTCCACTCAACTTTAACCCG
gir_dor_sp6-9_pair13	ATAGCCAATTGCAAACGAATGGTCT	CTGATCTGTTGAAACGTTTGCCACA	45,29	23,87	21,42	gir_dor_sp6-9	LS_HCR_gd	B3	GTCCCTGCCTCTATATCT	TT	CCACTCAACTTTAACCCG	TT	GTCCCTGCCTCTATATCTTTATAGCCAATTGCAAACGAATGGTCT	CTGATCTGTTGAAACGTTTGCCACATTCCACTCAACTTTAACCCG
gir_dor_sp6-9_pair14	TCGTCGGTATGAGTTTTCAGGTGCT	CCACATTTCGGGCAATTTATTTTCT	38,88	12,84	26,04	gir_dor_sp6-9	LS_HCR_gd	B3	GTCCCTGCCTCTATATCT	TT	CCACTCAACTTTAACCCG	TT	GTCCCTGCCTCTATATCTTTTCGTCGGTATGAGTTTTCAGGTGCT	CCACATTTCGGGCAATTTATTTTCTTTCCACTCAACTTTAACCCG
gir_dor_sp6-9_pair15	TTTAACTGCTCATTTCGACTAAAGT	ACAGTGTTATGAGTATAAATATGCT	55,33	26,67	28,66	gir_dor_sp6-9	LS_HCR_gd	B3	GTCCCTGCCTCTATATCT	TT	CCACTCAACTTTAACCCG	TT	GTCCCTGCCTCTATATCTTTTTTAACTGCTCATTTCGACTAAAGT	ACAGTGTTATGAGTATAAATATGCTTTCCACTCAACTTTAACCCG
gir_dor_sp6-9_pair16	AGGAACCATTATTTATTGTAACAAA	AACAAATAAAATCCACCAAAACTAA	65,66	31,8	33,86	gir_dor_sp6-9	LS_HCR_gd	B3	GTCCCTGCCTCTATATCT	TT	CCACTCAACTTTAACCCG	TT	GTCCCTGCCTCTATATCTTTAGGAACCATTATTTATTGTAACAAA	AACAAATAAAATCCACCAAAACTAATTCCACTCAACTTTAACCCG
gir_mul_ovo_pair1	TGTAAAGTGAGAAAATGTGACCCCA	GAGTTAATAAGAAAAGTGCTCATAA	59,06	21,26	37,8	gir_mul_ovo	LS_HCR_gm	B4	CCTCAACCTACCTCCAAC	AA	TCTCACCATATTCGCTTC	AT	CCTCAACCTACCTCCAACAATGTAAAGTGAGAAAATGTGACCCCA	GAGTTAATAAGAAAAGTGCTCATAAATTCTCACCATATTCGCTTC
gir_mul_ovo_pair2	CGGCTGTTCCTCATTATTGGCCAGC	TTTCCCAGTTTTCATTGATAAATCT	47,41	16,89	30,52	gir_mul_ovo	LS_HCR_gm	B4	CCTCAACCTACCTCCAAC	AA	TCTCACCATATTCGCTTC	AT	CCTCAACCTACCTCCAACAACGGCTGTTCCTCATTATTGGCCAGC	TTTCCCAGTTTTCATTGATAAATCTATTCTCACCATATTCGCTTC
gir_mul_ovo_pair3	ATCTATCATCCTTGACTTGAGCGTT	GAATGCAAGGATAATTATACGAACA	50,93	22,66	28,27	gir_mul_ovo	LS_HCR_gm	B4	CCTCAACCTACCTCCAAC	AA	TCTCACCATATTCGCTTC	AT	CCTCAACCTACCTCCAACAAATCTATCATCCTTGACTTGAGCGTT	GAATGCAAGGATAATTATACGAACAATTCTCACCATATTCGCTTC
gir_mul_ovo_pair4	TGAATGTACATCTTCATTGGAATGG	ATTAAGAAACTGTTTTCGTGGTATT	59,77	29,32	30,46	gir_mul_ovo	LS_HCR_gm	B4	CCTCAACCTACCTCCAAC	AA	TCTCACCATATTCGCTTC	AT	CCTCAACCTACCTCCAACAATGAATGTACATCTTCATTGGAATGG	ATTAAGAAACTGTTTTCGTGGTATTATTCTCACCATATTCGCTTC
gir_mul_ovo_pair5	AGAAATTGAGGAAAACCCGACAAAA	TTCAAAGTTGGCTTGATGAAACCAT	50,87	25,73	25,14	gir_mul_ovo	LS_HCR_gm	B4	CCTCAACCTACCTCCAAC	AA	TCTCACCATATTCGCTTC	AT	CCTCAACCTACCTCCAACAAAGAAATTGAGGAAAACCCGACAAA	TTCAAAGTTGGCTTGATGAAACCATATTCTCACCATATTCGCTTC
gir_mul_ovo_pair6	ATGTTGCTGAATTAGAAATCATTGA	TCTTTTGGAAATGAAGTATGGGATA	58,43	29,78	28,65	gir_mul_ovo	LS_HCR_gm	B4	CCTCAACCTACCTCCAAC	AA	TCTCACCATATTCGCTTC	AT	CCTCAACCTACCTCCAACAAATGTTGCTGAATTAGAAATCATTGA	TCTTTTGGAAATGAAGTATGGGATAATTCTCACCATATTCGCTTC
gir_mul_ovo_pair7	AGCTCGTCGATACATTTGATTCGAT	CTTCTGAAGAAGAAGTTCCAGTACT	48,7	21,13	27,57	gir_mul_ovo	LS_HCR_gm	B4	CCTCAACCTACCTCCAAC	AA	TCTCACCATATTCGCTTC	AT	CCTCAACCTACCTCCAACAAAGCTCGTCGATACATTTGATTCGAT	CTTCTGAAGAAGAAGTTCCAGTACTATTCTCACCATATTCGCTTC
gir_mul_ovo_pair8	CTAATATTCTCAAAATGAGTAGTCT	TCACTAAGATGAAAGTATTTCTCAA	66,75	35,82	30,93	gir_mul_ovo	LS_HCR_gm	B4	CCTCAACCTACCTCCAAC	AA	TCTCACCATATTCGCTTC	AT	CCTCAACCTACCTCCAACAACTAATATTCTCAAAATGAGTAGTCT	TCACTAAGATGAAAGTATTTCTCAAATTCTCACCATATTCGCTTC
gir_mul_ovo_pair9	CTCTTCAACACTCTCTTCAGATTGA	TCGTTTTATTTTAATGGTTTTAACA	58,18	23,96	34,23	gir_mul_ovo	LS_HCR_gm	B4	CCTCAACCTACCTCCAAC	AA	TCTCACCATATTCGCTTC	AT	CCTCAACCTACCTCCAACAACTCTTCAACACTCTCTTCAGATTGA	TCGTTTTATTTTAATGGTTTTAACAATTCTCACCATATTCGCTTC
gir_mul_ovo_pair10	TTTAAACTTTCCCGGATCTGTAAGA	CACAATTCCGTCCCCACCGTTTGTC	49,59	27,44	22,16	gir_mul_ovo	LS_HCR_gm	B4	CCTCAACCTACCTCCAAC	AA	TCTCACCATATTCGCTTC	AT	CCTCAACCTACCTCCAACAATTTAAACTTTCCCGGATCTGTAAGA	CACAATTCCGTCCCCACCGTTTGTCATTCTCACCATATTCGCTTC
gir_mul_ovo_pair11	TCTTTGAGGGAAATATTTTGTTGCA	GGGATCGAATGTGACGTTGCATATG	44,77	29,01	15,76	gir_mul_ovo	LS_HCR_gm	B4	CCTCAACCTACCTCCAAC	AA	TCTCACCATATTCGCTTC	AT	CCTCAACCTACCTCCAACAATCTTTGAGGGAAATATTTTGTTGCA	GGGATCGAATGTGACGTTGCATATGATTCTCACCATATTCGCTTC
gir_mul_ovo_pair12	TGCAAAGCCAGTCAAAATTGACCCC	AATCAATAAAGGGTTTGAAACAATA	48,39	15,66	32,73	gir_mul_ovo	LS_HCR_gm	B4	CCTCAACCTACCTCCAAC	AA	TCTCACCATATTCGCTTC	AT	CCTCAACCTACCTCCAACAATGCAAAGCCAGTCAAAATTGACCCC	AATCAATAAAGGGTTTGAAACAATAATTCTCACCATATTCGCTTC
gir_mul_ovo_pair13	CCTGTGTGGACTCTTGTATGCCTTT	CAACTTTGACATTTGTAAGGTTGAA	45,83	16,29	29,53	gir_mul_ovo	LS_HCR_gm	B4	CCTCAACCTACCTCCAAC	AA	TCTCACCATATTCGCTTC	AT	CCTCAACCTACCTCCAACAACCTGTGTGGACTCTTGTATGCCTTT	CAACTTTGACATTTGTAAGGTTGAAATTCTCACCATATTCGCTTC
gir_mul_ovo_pair14	GCTTTCTAACGAGCAACGCTGACTA	TTCGATCCCATGAATCTTCACTTGA	37,23	16,3	20,94	gir_mul_ovo	LS_HCR_gm	B4	CCTCAACCTACCTCCAAC	AA	TCTCACCATATTCGCTTC	AT	CCTCAACCTACCTCCAACAAGCTTTCTAACGAGCAACGCTGACTA	TTCGATCCCATGAATCTTCACTTGAATTCTCACCATATTCGCTTC
gir_mul_ovo_pair15	AGCTTATTTCGGCGCTCCTTATACA	CTATAACTGCAAATCTCACAAGGAA	47,11	21,76	25,35	gir_mul_ovo	LS_HCR_gm	B4	CCTCAACCTACCTCCAAC	AA	TCTCACCATATTCGCTTC	AT	CCTCAACCTACCTCCAACAAAGCTTATTTCGGCGCTCCTTATACA	CTATAACTGCAAATCTCACAAGGAAATTCTCACCATATTCGCTTC
gir_mul_ovo_pair16	GGTTTAGCCATGTAGTTTTACAGGA	AGGAATTGGGATGTTCTTGACTAAT	50,17	23,52	26,65	gir_mul_ovo	LS_HCR_gm	B4	CCTCAACCTACCTCCAAC	AA	TCTCACCATATTCGCTTC	AT	CCTCAACCTACCTCCAACAAGGTTTAGCCATGTAGTTTTACAGGA	AGGAATTGGGATGTTCTTGACTAATATTCTCACCATATTCGCTTC
gir_mul_ovo_pair17	TCCTTTTATAAGTTCTTCTTTGGCA	CTGATTGGGATACATTCAATTGTAT	64,75	29,26	35,48	gir_mul_ovo	LS_HCR_gm	B4	CCTCAACCTACCTCCAAC	AA	TCTCACCATATTCGCTTC	AT	CCTCAACCTACCTCCAACAATCCTTTTATAAGTTCTTCTTTGGCA	CTGATTGGGATACATTCAATTGTATATTCTCACCATATTCGCTTC
gir_mul_ovo_pair18	AATATACAGAATAATAGTCAAAATC	TGACGCAAAATGATGATGAACAATA	62,05	37,56	24,49	gir_mul_ovo	LS_HCR_gm	B4	CCTCAACCTACCTCCAAC	AA	TCTCACCATATTCGCTTC	AT	CCTCAACCTACCTCCAACAAAATATACAGAATAATAGTCAAAATC	TGACGCAAAATGATGATGAACAATAATTCTCACCATATTCGCTTC
gir_mul_six-1-2_pair1	CACGAGCCAAGCAACAGACATTCAA	GGCGAATTGGGTACCGGGATCGATA	26,97	12,15	14,82	gir_mul_six-1-2	LS_HCR_gm	B1	GAGGAGGGCAGCAAACG	AA	GAAGAGTCTTCCTTTACG	TA	GAGGAGGGCAGCAAACGGAACACGAGCCAAGCAACAGACATTC	GGCGAATTGGGTACCGGGATCGATATAGAAGAGTCTTCCTTTACG

gir_mul_six-1-2_pair2	AGGTTGCTTGTGTTTGTGAGGTCCT	CCGGTACGATGGGTTTCACACAAGA	27,05	13,58	13,47	gir_mul_six-1-2	LS_HCR_gm B1	GAGGAGGGCAGCAAACG	AA	GAAGAGTCTTCCTTTACG	TA	GAGGAGGGCAGCAAACGGAAAGGTTGCTTGTGTTTGTGAGGTC	CCGGTACGATGGGTTTCACACAAGATAGAAGAGTCTTCCTTTACG
gir_mul_six-1-2_pair3	CGTCGAATTTGCTCAACCCTCCTGC	GTGACGACTTAGATTCCACAGACCA	32,67	15,2	17,48	gir_mul_six-1-2	LS_HCR_gm B1	GAGGAGGGCAGCAAACG	AA	GAAGAGTCTTCCTTTACG	TA	GAGGAGGGCAGCAAACGGAACGTCGAATTTGCTCAACCCTCCTG	GTGACGACTTAGATTCCACAGACCATAGAAGAGTCTTCCTTTACG
gir_mul_six-1-2_pair4	TTCAAACCAACGAAAGCGTACTGAC	TATGGTCTCTTCCACCTTGCCAACA	36,17	20,18	15,98	gir_mul_six-1-2	LS_HCR_gm B1	GAGGAGGGCAGCAAACG	AA	GAAGAGTCTTCCTTTACG	TA	GAGGAGGGCAGCAAACGGAATTCAAACCAACGAAAGCGTACTG	TATGGTCTCTTCCACCTTGCCAACATAGAAGAGTCTTCCTTTACG
gir_mul_six-1-2_pair5	CTGAAATGACGAGTTTGACGACGAC	CGTCACCACGGGAAAAGAAAGATCT	37,08	14,8	22,28	gir_mul_six-1-2	LS_HCR_gm B1	GAGGAGGGCAGCAAACG	AA	GAAGAGTCTTCCTTTACG	TA	GAGGAGGGCAGCAAACGGAACTGAAATGACGAGTTTGACGACG	CGTCACCACGGGAAAAGAAAGATCTTAGAAGAGTCTTCCTTTACG
gir_mul_six-1-2_pair6	ACTCCTCAGGACTCAGTGATCTCGT	CTCGCAGCACCGACTTTAGATTATA	38,76	16,8	21,96	gir_mul_six-1-2	LS_HCR_gm B1	GAGGAGGGCAGCAAACG	AA	GAAGAGTCTTCCTTTACG	TA	GAGGAGGGCAGCAAACGGAAACTCCTCAGGACTCAGTGATCTCG	CTCGCAGCACCGACTTTAGATTATATAGAAGAGTCTTCCTTTACG
gir_mul_six-1-2_pair7	GATCTCTCCGCTTCTGATGCCGACA	GCTGCTGAAAATAAAGACAAACATG	40,27	15,16	25,12	gir_mul_six-1-2	LS_HCR_gm B1	GAGGAGGGCAGCAAACG	AA	GAAGAGTCTTCCTTTACG	TA	GAGGAGGGCAGCAAACGGAAGATCTCTCCGCTTCTGATGCCGAC	GCTGCTGAAAATAAAGACAAACATGTAGAAGAGTCTTCCTTTACG
gir_mul_six-1-2_pair8	TCGACGAAAATATCCCTTACCGAGA	ACTAGGAGCTGTCGCGAAATATCGA	40,5	20,64	19,86	gir_mul_six-1-2	LS_HCR_gm B1	GAGGAGGGCAGCAAACG	AA	GAAGAGTCTTCCTTTACG	TA	GAGGAGGGCAGCAAACGGAATCGACGAAAATATCCCTTACCGA	ACTAGGAGCTGTCGCGAAATATCGATAGAAGAGTCTTCCTTTACG
gir_mul_six-1-2_pair9	CCATAACCCAGACTATGATACGCCT	TTTCTCTCAGAATACCTATGGCAAT	41,92	17,55	24,37	gir_mul_six-1-2	LS_HCR_gm B1	GAGGAGGGCAGCAAACG	AA	GAAGAGTCTTCCTTTACG	TA	GAGGAGGGCAGCAAACGGAACCATAACCCAGACTATGATACGC	TTTCTCTCAGAATACCTATGGCAATTAGAAGAGTCTTCCTTTACG
gir_mul_six-1-2_pair10	CTGGTATTCAAGGAGGTTATTGTAC	CCGTGAGTCCGTTCAATACAAGTGC	42,11	26,01	16,1	gir_mul_six-1-2	LS_HCR_gm B1	GAGGAGGGCAGCAAACG	AA	GAAGAGTCTTCCTTTACG	TA	GAGGAGGGCAGCAAACGGAACTGGTATTCAAGGAGGTTATTGT	CCGTGAGTCCGTTCAATACAAGTGCTAGAAGAGTCTTCCTTTACG
gir_mul_six-1-2_pair11	CCTTCCCATAACCGGACAATATCCC	TTCTTACTTTAGTTCGGGGTTACAT	43,93	18,41	25,52	gir_mul_six-1-2	LS_HCR_gm B1	GAGGAGGGCAGCAAACG	AA	GAAGAGTCTTCCTTTACG	TA	GAGGAGGGCAGCAAACGGAACCTTCCCATAACCGGACAATATCC	TTCTTACTTTAGTTCGGGGTTACATTAGAAGAGTCTTCCTTTACG
gir_mul_six-1-2_pair12	TTCAAGCGCTCTGGTTACAAGCTCA	CGTTCTCACCGCATAATCATTATAA	45,27	17,73	27,54	gir_mul_six-1-2	LS_HCR_gm B1	GAGGAGGGCAGCAAACG	AA	GAAGAGTCTTCCTTTACG	TA	GAGGAGGGCAGCAAACGGAATTCAAGCGCTCTGGTTACAAGCTC	CGTTCTCACCGCATAATCATTATAATAGAAGAGTCTTCCTTTACG
gir_mul_six-1-2_pair13	CACTCGATGGCCCGGTTGTTTCGTC	AATTTTCTAATTCAGGTGACTACAA	45,39	15,4	30	gir_mul_six-1-2	LS_HCR_gm B1	GAGGAGGGCAGCAAACG	AA	GAAGAGTCTTCCTTTACG	TA	GAGGAGGGCAGCAAACGGAACACTCGATGGCCCGGTTGTTTCGT	AATTTTCTAATTCAGGTGACTACAATAGAAGAGTCTTCCTTTACG
gir_mul_six-1-2_pair14	CGATGAAGAAACTACAATCGTACCC	AAGAAGGAAACCATGAAAATGGAGG	45,58	21,04	24,54	gir_mul_six-1-2	LS_HCR_gm B1	GAGGAGGGCAGCAAACG	AA	GAAGAGTCTTCCTTTACG	TA	GAGGAGGGCAGCAAACGGAACGATGAAGAAACTACAATCGTAC	AAGAAGGAAACCATGAAAATGGAGGTAGAAGAGTCTTCCTTTACG
gir_mul_six-1-2_pair15	ATTTCAAAGAGCTTTATCGAATATT	AAGCAGCTGTCGCATTTCATAGACA	52,38	34,68	17,69	gir_mul_six-1-2	LS_HCR_gm B1	GAGGAGGGCAGCAAACG	AA	GAAGAGTCTTCCTTTACG	TA	GAGGAGGGCAGCAAACGGAAATTTCAAAGAGCTTTATCGAATAT	AAGCAGCTGTCGCATTTCATAGACATAGAAGAGTCTTCCTTTACG
gir_mul_six-1-2_pair16	TCTGAATTACGATAAAATTTTCACC	TTCCATGTCGAGTACAAATTCCACT	56,23	35,99	20,25	gir_mul_six-1-2	LS_HCR_gm B1	GAGGAGGGCAGCAAACG	AA	GAAGAGTCTTCCTTTACG	TA	GAGGAGGGCAGCAAACGGAATCTGAATTACGATAAAATTTTCAC	TTCCATGTCGAGTACAAATTCCACTTAGAAGAGTCTTCCTTTACG
gir_mul_six-1-2_pair17	TGATCATTCAACAAAATTATTAAAG	CACTAGTTATATGAATTCAGGAAAA	73,25	37,44	35,81	gir_mul_six-1-2	LS_HCR_gm B1	GAGGAGGGCAGCAAACG	AA	GAAGAGTCTTCCTTTACG	TA	GAGGAGGGCAGCAAACGGAATGATCATTCAACAAAATTATTAAA	CACTAGTTATATGAATTCAGGAAAATAGAAGAGTCTTCCTTTACG
gir_mul_sp6-9_pair1	CAGTGAAGTGAAATCCATATTTGAT	TGACTGATAACTGTTCTCGACATTG	53,63	31,43	22,2	gir_mul_sp6-9	LS_HCR_gm B3	GTCCCTGCCTCTATATCT	TT	CCACTCAACTTTAACCCG	TT	GTCCCTGCCTCTATATCTTTCAGTGAAGTGAAATCCATATTTGAT	TGACTGATAACTGTTCTCGACATTGTTCCACTCAACTTTAACCCG
gir_mul_sp6-9_pair2	GAAACATCATTTATTTTCCTGACAG	TTGTTAAATTCAGTTTCATGTAAAA	64,51	30,3	34,22	gir_mul_sp6-9	LS_HCR_gm B3	GTCCCTGCCTCTATATCT	TT	CCACTCAACTTTAACCCG	TT	GTCCCTGCCTCTATATCTTTGAAACATCATTTATTTTCCTGACAG	TTGTTAAATTCAGTTTCATGTAAAATTCCACTCAACTTTAACCCG
gir_mul_sp6-9_pair3	AATCTCCATAAATTGTGAAAAGCAG	TGGTTGGAATCCATTGTTGGCAGAT	50,26	29,68	20,58	gir_mul_sp6-9	LS_HCR_gm B3	GTCCCTGCCTCTATATCT	TT	CCACTCAACTTTAACCCG	TT	GTCCCTGCCTCTATATCTTTAATCTCCATAAATTGTGAAAAGCAG	TGGTTGGAATCCATTGTTGGCAGATTTCCACTCAACTTTAACCCG
gir_mul_sp6-9_pair4	TTATCATTCATAGATGAACTTTGAG	GGTATGGAAAGGAGTGGATGAAACA	54,43	33,8	20,63	gir_mul_sp6-9	LS_HCR_gm B3	GTCCCTGCCTCTATATCT	TT	CCACTCAACTTTAACCCG	TT	GTCCCTGCCTCTATATCTTTTTATCATTCATAGATGAACTTTGAG	GGTATGGAAAGGAGTGGATGAAACATTCCACTCAACTTTAACCCG
gir_mul_sp6-9_pair5	CTTTGAGTTGAAAAGTTTTGATAGT	TCACTAGTAGAATTAGATGGGTATT	59,22	31,16	28,06	gir_mul_sp6-9	LS_HCR_gm B3	GTCCCTGCCTCTATATCT	TT	CCACTCAACTTTAACCCG	TT	GTCCCTGCCTCTATATCTTTCTTTGAGTTGAAAAGTTTTGATAGT	TCACTAGTAGAATTAGATGGGTATTTTCCACTCAACTTTAACCCG
gir_mul_sp6-9_pair6	AGGTTGGATTCTGGCAAATTCAGTG	AAAATATGGGACTGGGGTAAATTGA	44,86	19,84	25,02	gir_mul_sp6-9	LS_HCR_gm B3	GTCCCTGCCTCTATATCT	TT	CCACTCAACTTTAACCCG	TT	GTCCCTGCCTCTATATCTTTAGGTTGGATTCTGGCAAATTCAGTG	AAAATATGGGACTGGGGTAAATTGATTCCACTCAACTTTAACCCG
gir_mul_sp6-9_pair7	TTTTGGAAGAGATTGAAAATTATAT	TGAAGAAATGAATGGGTTTAAGTAA	67,24	38,58	28,66	gir_mul_sp6-9	LS_HCR_gm B3	GTCCCTGCCTCTATATCT	TT	CCACTCAACTTTAACCCG	TT	GTCCCTGCCTCTATATCTTTTTTTGGAAGAGATTGAAAATTATAT	TGAAGAAATGAATGGGTTTAAGTAATTCCACTCAACTTTAACCCG
gir_mul_sp6-9_pair8	GATTCGGATATCTTGGAAGAGAACT	GACAATTTGGACAGGTGCAAGCGGC	38,18	25,86	12,32	gir_mul_sp6-9	LS_HCR_gm B3	GTCCCTGCCTCTATATCT	TT	CCACTCAACTTTAACCCG	TT	GTCCCTGCCTCTATATCTTTGATTCGGATATCTTGGAAGAGAACT	GACAATTTGGACAGGTGCAAGCGGCTTCCACTCAACTTTAACCCG
gir_mul_sp6-9_pair9	TTCTTTGACAGAAAGCCAGAAACCT	CCCGCTATGTGGCAATTATGGGTAG	36,34	20,98	15,35	gir_mul_sp6-9	LS_HCR_gm B3	GTCCCTGCCTCTATATCT	TT	CCACTCAACTTTAACCCG	TT	GTCCCTGCCTCTATATCTTTTTCTTTGACAGAAAGCCAGAAACCT	CCCGCTATGTGGCAATTATGGGTAGTTCCACTCAACTTTAACCCG
gir_mul_sp6-9_pair10	CAAATGACTGGGTTTTGAATACAGT	TCCTGTATGCCATCTTAAATGCGCT	45,06	25,83	19,23	gir_mul_sp6-9	LS_HCR_gm B3	GTCCCTGCCTCTATATCT	TT	CCACTCAACTTTAACCCG	TT	GTCCCTGCCTCTATATCTTTCAAATGACTGGGTTTTGAATACAGT	TCCTGTATGCCATCTTAAATGCGCTTTCCACTCAACTTTAACCCG
gir_mul_sp6-9_pair11	ATAGCCAATTACAAACAAATGGTCT	CTGATCTATTGAAACGCTTTCCACA	48,82	25,39	23,43	gir_mul_sp6-9	LS_HCR_gm B3	GTCCCTGCCTCTATATCT	TT	CCACTCAACTTTAACCCG	TT	GTCCCTGCCTCTATATCTTTATAGCCAATTACAAACAAATGGTCT	CTGATCTATTGAAACGCTTTCCACATTCCACTCAACTTTAACCCG
gir_mul_sp6-9_pair12	TCGTCGGTATGAGTTTTCAGATGCT	CCACATTTCGGGCAATTAATCTTCT	39,08	16,43	22,65	gir_mul_sp6-9	LS_HCR_gm B3	GTCCCTGCCTCTATATCT	TT	CCACTCAACTTTAACCCG	TT	GTCCCTGCCTCTATATCTTTTCGTCGGTATGAGTTTTCAGATGCT	CCACATTTCGGGCAATTAATCTTCTTTCCACTCAACTTTAACCCG
gir_mul_sp6-9_pair13	TGCTTATTTAACTGCTCATTTCGAC	TTATCAACAGTGTTATGAGTGTAAA	54,66	24,39	30,27	gir_mul_sp6-9	LS_HCR_gm B3	GTCCCTGCCTCTATATCT	TT	CCACTCAACTTTAACCCG	TT	GTCCCTGCCTCTATATCTTTTGCTTATTTAACTGCTCATTTCGAC	TTATCAACAGTGTTATGAGTGTAAATTCCACTCAACTTTAACCCG
gm_1329_1_1_4_pair1	TCGGTTGCTCATCGATTGCCATTCC	GTGGCATTTCTCTCCCTCTAATCGT	30,2	13,61	16,59	gm_1329_1_1_4	LS_HCR_gm B2	CCTCGTAAATCCTCATCA	AA	ATCATCCAGTAAACCGCC	AA	CCTCGTAAATCCTCATCAAATCGGTTGCTCATCGATTGCCATTCC	GTGGCATTTCTCTCCCTCTAATCGTAAATCATCCAGTAAACCGCC
gm_1329_1_1_4_pair2	CGTCTTGGTCTTGTTGTTGCTGTGT	ATTGCAAATCGTCGTTGGAGTCCCC	30,35	14,27	16,08	gm_1329_1_1_4	LS_HCR_gm B2	CCTCGTAAATCCTCATCA	AA	ATCATCCAGTAAACCGCC	AA	CCTCGTAAATCCTCATCAAACGTCTTGGTCTTGTTGTTGCTGTGT	ATTGCAAATCGTCGTTGGAGTCCCCAAATCATCCAGTAAACCGCC
gm_1329_1_1_4_pair3	GTCGACCGTCAAAACAAAATGGCGT	CGTGCCACTCGACAACGGTAAATAA	30,72	14,11	16,6	gm_1329_1_1_4	LS_HCR_gm B2	CCTCGTAAATCCTCATCA	AA	ATCATCCAGTAAACCGCC	AA	CCTCGTAAATCCTCATCAAAGTCGACCGTCAAAACAAAATGGCGT	CGTGCCACTCGACAACGGTAAATAAAAATCATCCAGTAAACCGCC
gm_1329_1_1_4_pair4	CGTCTTCATTCGTTCCTCGGGTGAT	CCAAATACAACAAAGTCGACGCCGA	31,85	16,71	15,13	gm_1329_1_1_4	LS_HCR_gm B2	CCTCGTAAATCCTCATCA	AA	ATCATCCAGTAAACCGCC	AA	CCTCGTAAATCCTCATCAAACGTCTTCATTCGTTCCTCGGGTGAT	CCAAATACAACAAAGTCGACGCCGAAAATCATCCAGTAAACCGCC
gm_1329_1_1_4_pair5	CGAAAACACCGACGCCTGCACAGAC	AATGTACTGGGAGAATTTGGCGCTA	32,28	14,69	17,59	gm_1329_1_1_4	LS_HCR_gm B2	CCTCGTAAATCCTCATCA	AA	ATCATCCAGTAAACCGCC	AA	CCTCGTAAATCCTCATCAAACGAAAACACCGACGCCTGCACAGAC	AATGTACTGGGAGAATTTGGCGCTAAAATCATCCAGTAAACCGCC
gm_1329_1_1_4_pair6	TCTCGATTACTTCCATTACGTTGCG	TCGGCCACAACTTTCCTTCGTCGAT	32,46	18,98	13,48	gm_1329_1_1_4	LS_HCR_gm B2	CCTCGTAAATCCTCATCA	AA	ATCATCCAGTAAACCGCC	AA	CCTCGTAAATCCTCATCAAATCTCGATTACTTCCATTACGTTGCG	TCGGCCACAACTTTCCTTCGTCGATAAATCATCCAGTAAACCGCC
gm_1329_1_1_4_pair7	ATCGATGACTTGTGCCCTTGGGAAT	GGTTTCTGTCGACAACCAACACCGC	33,4	17,01	16,39	gm_1329_1_1_4	LS_HCR_gm B2	CCTCGTAAATCCTCATCA	AA	ATCATCCAGTAAACCGCC	AA	CCTCGTAAATCCTCATCAAAATCGATGACTTGTGCCCTTGGGAAT	GGTTTCTGTCGACAACCAACACCGCAAATCATCCAGTAAACCGCC
gm_1329_1_1_4_pair8	GTTGTAAAACACTGGGAGCCATCGC	AGAGGTGCGTCAGAGAATAGGTAAT	34,61	17,41	17,21	gm_1329_1_1_4	LS_HCR_gm B2	CCTCGTAAATCCTCATCA	AA	ATCATCCAGTAAACCGCC	AA	CCTCGTAAATCCTCATCAAAGTTGTAAAACACTGGGAGCCATCGC	AGAGGTGCGTCAGAGAATAGGTAATAAATCATCCAGTAAACCGCC
gm_1329_1_1_4_pair9	CTTTCCCAACGAGTTCTGCCAGTCG	TAGTTGGAGGATTGGCACCCCGATG	34,75	15,12	19,63	gm_1329_1_1_4	LS_HCR_gm B2	CCTCGTAAATCCTCATCA	AA	ATCATCCAGTAAACCGCC	AA	CCTCGTAAATCCTCATCAAACTTTCCCAACGAGTTCTGCCAGTCG	TAGTTGGAGGATTGGCACCCCGATGAAATCATCCAGTAAACCGCC
gm_1329_1_1_4_pair10	CCCACGGATCATAGCCAAGTTTCGA	TCGTCGGTGCCATTTTCAAATTAAT	35,5	13,72	21,77	gm_1329_1_1_4	LS_HCR_gm B2	CCTCGTAAATCCTCATCA	AA	ATCATCCAGTAAACCGCC	AA	CCTCGTAAATCCTCATCAAACCCACGGATCATAGCCAAGTTTCGA	TCGTCGGTGCCATTTTCAAATTAATAAATCATCCAGTAAACCGCC
gm_1329_1_1_4_pair11	CTCGGTAAATAATGAATCTTTGCGG	ACGCCAACTGAGAATCACCGACTCC	36,27	23,75	12,52	gm_1329_1_1_4	LS_HCR_gm B2	CCTCGTAAATCCTCATCA	AA	ATCATCCAGTAAACCGCC	AA	CCTCGTAAATCCTCATCAAACTCGGTAAATAATGAATCTTTGCGG	ACGCCAACTGAGAATCACCGACTCCAAATCATCCAGTAAACCGCC
gm_1329_1_1_4_pair12	ATTGTCGACCGATTTTCTCTTGTCC	CTTGTTCACAGTTCGATGAGAGGAA	36,88	19,24	17,64	gm_1329_1_1_4	LS_HCR_gm B2	CCTCGTAAATCCTCATCA	AA	ATCATCCAGTAAACCGCC	AA	CCTCGTAAATCCTCATCAAAATTGTCGACCGATTTTCTCTTGTCC	CTTGTTCACAGTTCGATGAGAGGAAAAATCATCCAGTAAACCGCC
gm_1329_1_1_4_pair13	CTTTTGGCGCTTCTTCAACTTCCCC	CGGGGATTGATAAACTTTGATCAGG	37	15,53	21,47	gm_1329_1_1_4	LS_HCR_gm B2	CCTCGTAAATCCTCATCA	AA	ATCATCCAGTAAACCGCC	AA	CCTCGTAAATCCTCATCAAACTTTTGGCGCTTCTTCAACTTCCCC	CGGGGATTGATAAACTTTGATCAGGAAATCATCCAGTAAACCGCC
gm_1329_1_1_4_pair14	AAACAGAACTCTCATTCAACACACG	CGATTTGGTCGCGTCGATAAACACT	40,67	23,84	16,83	gm_1329_1_1_4	LS_HCR_gm B2	CCTCGTAAATCCTCATCA	AA	ATCATCCAGTAAACCGCC	AA	CCTCGTAAATCCTCATCAAAAAACAGAACTCTCATTCAACACACG	CGATTTGGTCGCGTCGATAAACACTAAATCATCCAGTAAACCGCC
gm_1329_1_1_4_pair15	ATTGGGATTTCCGCCATCTTTAAAG	AACCTTTTCGTCAACAATCGTACCC	43,67	24,78	18,89	gm_1329_1_1_4	LS_HCR_gm B2	CCTCGTAAATCCTCATCA	AA	ATCATCCAGTAAACCGCC	AA	CCTCGTAAATCCTCATCAAAATTGGGATTTCCGCCATCTTTAAAG	AACCTTTTCGTCAACAATCGTACCCAAATCATCCAGTAAACCGCC
gm_1329_1_1_4_pair16	ATCCCGTCCGATTTTATCGACAACC	ATTGGATTTTAGGTTAACCGCGCGT	44,22	21,4	22,82	gm_1329_1_1_4	LS_HCR_gm B2	CCTCGTAAATCCTCATCA	AA	ATCATCCAGTAAACCGCC	AA	CCTCGTAAATCCTCATCAAAATCCCGTCCGATTTTATCGACAACC	ATTGGATTTTAGGTTAACCGCGCGTAAATCATCCAGTAAACCGCC
gm_1329_1_1_4_pair17	ATTTTCGCGTTGATTTTATTATCCA	ACCTCCCCAAATTTCCAATCGTCGG	44,41	28,84	15,57	gm_1329_1_1_4	LS_HCR_gm B2	CCTCGTAAATCCTCATCA	AA	ATCATCCAGTAAACCGCC	AA	CCTCGTAAATCCTCATCAAAATTTTCGCGTTGATTTTATTATCCA	ACCTCCCCAAATTTCCAATCGTCGGAAATCATCCAGTAAACCGCC
gm_1329_1_1_4_pair18	AAAAGCCGTTCTCTAATCCTGTCGT	GCAGTATAAACAGACGTCTAAAGGT	47,32	21,39	25,94	gm_1329_1_1_4	LS_HCR_gm B2	CCTCGTAAATCCTCATCA	AA	ATCATCCAGTAAACCGCC	AA	CCTCGTAAATCCTCATCAAAAAAAGCCGTTCTCTAATCCTGTCGT	GCAGTATAAACAGACGTCTAAAGGTAAATCATCCAGTAAACCGCC
gm_1329_1_1_4_pair19	AACGGGCCGTCGGTTTTAATATTAA	TTCTCAGAAACAACGTCGTACATAA	47,35	25,69	21,66	gm_1329_1_1_4	LS_HCR_gm B2	CCTCGTAAATCCTCATCA	AA	ATCATCCAGTAAACCGCC	AA	CCTCGTAAATCCTCATCAAAAAACGGGCCGTCGGTTTTAATATTAA	TTCTCAGAAACAACGTCGTACATAAAAATCATCCAGTAAACCGCC
gm_1329_1_1_4_pair20	CGTTTTAATCGAGAAATAACGAACG	CGATTTCTTAGATTCGGAGCGATCG	47,76	26,16	21,6	gm_1329_1_1_4	LS_HCR_gm B2	CCTCGTAAATCCTCATCA	AA	ATCATCCAGTAAACCGCC	AA	CCTCGTAAATCCTCATCAAACGTTTTAATCGAGAAATAACGAACG	CGATTTCTTAGATTCGGAGCGATCGAAATCATCCAGTAAACCGCC
gm_2585_0_1_3_pair1	TGCGATTCCGGACATACTTGCACTC	ACCTTGTCTCATTCCACCGGCCATT	26,7	15,75	10,95	gm_2585_0_1_3	LS_HCR_gm B5	CTCACTCCCAATCTCTAT	AA	CTACCCTACAAATCCAAT	AA	CTCACTCCCAATCTCTATAATGCGATTCCGGACATACTTGCACTC	ACCTTGTCTCATTCCACCGGCCATTAACTACCCTACAAATCCAAT
gm_2585_0_1_3_pair2	GGCAAGTTTAGAGACAATGTGGACA	GGACTAAGAGAATGGGGTCCGATTC	33,47	16,82	16,65	gm_2585_0_1_3	LS_HCR_gm B5	CTCACTCCCAATCTCTAT	AA	CTACCCTACAAATCCAAT	AA	CTCACTCCCAATCTCTATAAGGCAAGTTTAGAGACAATGTGGACA	GGACTAAGAGAATGGGGTCCGATTCAACTACCCTACAAATCCAAT
gm_2585_0_1_3_pair3	ACGGAAAGTGGTCCATGATGAATTG	GCCATCGTTGTTGGTTCTGACGGTA	34,61	18,31	16,3	gm_2585_0_1_3	LS_HCR_gm B5	CTCACTCCCAATCTCTAT	AA	CTACCCTACAAATCCAAT	AA	CTCACTCCCAATCTCTATAAACGGAAAGTGGTCCATGATGAATTG	GCCATCGTTGTTGGTTCTGACGGTAAACTACCCTACAAATCCAAT
gm_2585_0_1_3_pair4	TGCAGCTCTTTGACTCTGTCTTCCT	TTCTGTGGCATTGGATCCAGGACCG	34,9	18,04	16,87	gm_2585_0_1_3	LS_HCR_gm B5	CTCACTCCCAATCTCTAT	AA	CTACCCTACAAATCCAAT	AA	CTCACTCCCAATCTCTATAATGCAGCTCTTTGACTCTGTCTTCCT	TTCTGTGGCATTGGATCCAGGACCGAACTACCCTACAAATCCAAT
gm_2585_0_1_3_pair5	TCCAGGCTTTTCAGGACTTCTACTT	TGGATGAAGCGTCCAATAAGAACCC	36,65	17,91	18,74	gm_2585_0_1_3	LS_HCR_gm B5	CTCACTCCCAATCTCTAT	AA	CTACCCTACAAATCCAAT	AA	CTCACTCCCAATCTCTATAATCCAGGCTTTTCAGGACTTCTACTT	TGGATGAAGCGTCCAATAAGAACCCAACTACCCTACAAATCCAAT
gm_2585_0_1_3_pair6	GGCGGCGTATAGACTATCATGGGGA	CTCGAGATCCATTGATGATCCAGCT	36,77	16,52	20,25	gm_2585_0_1_3	LS_HCR_gm B5	CTCACTCCCAATCTCTAT	AA	CTACCCTACAAATCCAAT	AA	CTCACTCCCAATCTCTATAAGGCGGCGTATAGACTATCATGGGGA	CTCGAGATCCATTGATGATCCAGCTAACTACCCTACAAATCCAAT
gm_2585_0_1_3_pair7	GGCGGGATATTTGAATAGTTTGAGT	TCGTAGGCTGTCATTGTCGATGTGG	37,02	24,51	12,51	gm_2585_0_1_3	LS_HCR_gm B5	CTCACTCCCAATCTCTAT	AA	CTACCCTACAAATCCAAT	AA	CTCACTCCCAATCTCTATAAGGCGGGATATTTGAATAGTTTGAGT	TCGTAGGCTGTCATTGTCGATGTGGAACTACCCTACAAATCCAAT
gm_2585_0_1_3_pair8	AATTGACGGACCGGTTGGCAGAGAA	TGAGATACCCATCGGATTCATACTG	37,99	13,05	24,94	gm_2585_0_1_3	LS_HCR_gm B5	CTCACTCCCAATCTCTAT	AA	CTACCCTACAAATCCAAT	AA	CTCACTCCCAATCTCTATAAAATTGACGGACCGGTTGGCAGAGAA	TGAGATACCCATCGGATTCATACTGAACTACCCTACAAATCCAAT
gm_2585_0_1_3_pair9	TCATTTCTACCGGTGAGCAGTTTTC	GGGGTTGTGGAGGTTGACTGGACAT	38	23,5	14,5	gm_2585_0_1_3	LS_HCR_gm B5	CTCACTCCCAATCTCTAT	AA	CTACCCTACAAATCCAAT	AA	CTCACTCCCAATCTCTATAATCATTTCTACCGGTGAGCAGTTTTC	GGGGTTGTGGAGGTTGACTGGACATAACTACCCTACAAATCCAAT

gm_2585_0_1_3_pair10	TCGGCATTATCACTTGCTTCTTGAG	TGTTTGATATTGGCCTTTGAATCGC	40,49	21,63	18,86	gm_2585_0_1_3	LS_HCR_gm	B5	CTCACTCCCAATCTCTAT	AA	CTACCCTACAAATCCAAT	AA	CTCACTCCCAATCTCTATAATCGGCATTATCACTTGCTTCTTGAG	TGTTTGATATTGGCCTTTGAATCGCAACTACCCTACAAATCCAAT
gm_2585_0_1_3_pair11	GGTAAGGATACGGAACTTTGTTCGT	TACGGATGACCAGAGAGAGATATGG	40,78	21,03	19,75	gm_2585_0_1_3	LS_HCR_gm	B5	CTCACTCCCAATCTCTAT	AA	CTACCCTACAAATCCAAT	AA	CTCACTCCCAATCTCTATAAGGTAAGGATACGGAACTTTGTTCGT	TACGGATGACCAGAGAGAGATATGGAACTACCCTACAAATCCAAT
gm_2585_0_1_3_pair12	AACTGTAAGGAGGTTTCGCATGGGT	GAATCGCCATTGTTATTAAACTGAT	42,45	14,35	28,1	gm_2585_0_1_3	LS_HCR_gm	B5	CTCACTCCCAATCTCTAT	AA	CTACCCTACAAATCCAAT	AA	CTCACTCCCAATCTCTATAAAACTGTAAGGAGGTTTCGCATGGGT	GAATCGCCATTGTTATTAAACTGATAACTACCCTACAAATCCAAT
gm_2585_0_1_3_pair13	TATCACTGTTACTTGCGAGGGCACT	GGGTCATTTACTGATTCTAAAGGCT	43,86	20,59	23,26	gm_2585_0_1_3	LS_HCR_gm	B5	CTCACTCCCAATCTCTAT	AA	CTACCCTACAAATCCAAT	AA	CTCACTCCCAATCTCTATAATATCACTGTTACTTGCGAGGGCACT	GGGTCATTTACTGATTCTAAAGGCTAACTACCCTACAAATCCAAT
gm_2585_0_1_3_pair14	CGATCGATTGAAAATGGATTACTGG	GCGGCTGCTAAACTAGGATGCATCA	44,56	27,67	16,89	gm_2585_0_1_3	LS_HCR_gm	B5	CTCACTCCCAATCTCTAT	AA	CTACCCTACAAATCCAAT	AA	CTCACTCCCAATCTCTATAACGATCGATTGAAAATGGATTACTGG	GCGGCTGCTAAACTAGGATGCATCAAACTACCCTACAAATCCAAT
gm_2585_0_1_3_pair15	ATATGTCAGAGATCCTGGTAAACTG	TGAATTGTGTTGGGAACCTTCGTAT	46,62	24,11	22,51	gm_2585_0_1_3	LS_HCR_gm	B5	CTCACTCCCAATCTCTAT	AA	CTACCCTACAAATCCAAT	AA	CTCACTCCCAATCTCTATAAATATGTCAGAGATCCTGGTAAACTG	TGAATTGTGTTGGGAACCTTCGTATAACTACCCTACAAATCCAAT
gm_2585_0_1_3_pair16	CTCGTACTTGGTTAACGATCACTCT	TTTTCCCAACATCTCTTTATCAACA	50,59	21,62	28,97	gm_2585_0_1_3	LS_HCR_gm	B5	CTCACTCCCAATCTCTAT	AA	CTACCCTACAAATCCAAT	AA	CTCACTCCCAATCTCTATAACTCGTACTTGGTTAACGATCACTCT	TTTTCCCAACATCTCTTTATCAACAAACTACCCTACAAATCCAAT
gm_2585_0_1_3_pair17	TGGATTATAATGATGTTGAGGTGGT	TTGTTGACTTGAATAAAACTGTTGA	54,99	26,68	28,31	gm_2585_0_1_3	LS_HCR_gm	B5	CTCACTCCCAATCTCTAT	AA	CTACCCTACAAATCCAAT	AA	CTCACTCCCAATCTCTATAATGGATTATAATGATGTTGAGGTGGT	TTGTTGACTTGAATAAAACTGTTGAAACTACCCTACAAATCCAAT
gm_2585_0_1_3_pair18	GAATTCTTATCTCTTGGACTTTCGC	TGATATGGACGATTATTATTGGAAA	55,99	25,65	30,34	gm_2585_0_1_3	LS_HCR_gm	B5	CTCACTCCCAATCTCTAT	AA	CTACCCTACAAATCCAAT	AA	CTCACTCCCAATCTCTATAAGAATTCTTATCTCTTGGACTTTCGC	TGATATGGACGATTATTATTGGAAAAACTACCCTACAAATCCAAT
gm_2585_0_1_3_pair19	TGTCTTCTTAAATAACAACCATTTT	TCTCGATGAGGATCTTTGAATCTCT	61,85	33,17	28,67	gm_2585_0_1_3	LS_HCR_gm	B5	CTCACTCCCAATCTCTAT	AA	CTACCCTACAAATCCAAT	AA	CTCACTCCCAATCTCTATAATGTCTTCTTAAATAACAACCATTTT	TCTCGATGAGGATCTTTGAATCTCTAACTACCCTACAAATCCAAT
