# Supplementary material for: Reduced adult stem cell fate specification led to eye reduction in cave planarians
Source: Nat Commun. 2025 Jan 2;16:304. doi: 10.1038/s41467-024-54478-6 (PMC11696554; doi:10.1038/s41467-024-54478-6)
Supplement: Supplementary file 3 — Description of Additional Supplementary Files [file 41467_2024_54478_MOESM3_ESM.pdf]

Description of Additional Supplementary Files

File Name: Supplementary Data 1
Description: FASTA file with all the *G. multidiverticulata* ortholog eye genes used in this study.

File Name: Supplementary Movie 1
Description: *Girardia dorotocephala* three-dimensional spatial signal visualization of neoblasts (piwi-1 – magenta) and cell nuclei (DAPI – gray) tissues using whole-mount FISH and generated by Imaris. Video highlight segmentation and detection of positive cells.

File Name: Supplementary Movie 2
Description: *Girardia multidiverticulata* three-dimensional spatial signal visualization of neoblasts (*piwi-1* – magenta) and cell nuclei (DAPI – gray) tissues using whole-mount FISH and generated by Imaris. Video highlight segmentation and detection of positive cells.
